# HIRA-mediated loading of histone variant H3.3 controls androgen-induced transcription by regulation of AR/BRD4 complex assembly at enhancers

**DOI:** 10.1093/nar/gkad700

**Published:** 2023-08-28

**Authors:** Viacheslav M Morozov, Alberto Riva, Sadia Sarwar, Wan-Ju Kim, Jianping Li, Lei Zhou, Jonathan D Licht, Yehia Daaka, Alexander M Ishov

**Affiliations:** Department of Anatomy and Cell Biology, University of Florida College of Medicine, Gainesville, FL, USA; Interdisciplinary Center for Biotechnology Research, University of Florida, Gainesville, FL, USA; Department of Anatomy and Cell Biology, University of Florida College of Medicine, Gainesville, FL, USA; Department of Anatomy and Cell Biology, University of Florida College of Medicine, Gainesville, FL, USA; Division of Hematology/Oncology, University of Florida College of Medicine, Gainesville, FL, USA; Department of Molecular Genetics & Microbiology, University of Florida College of Medicine, Gainesville, FL, USA; University of Florida Health Cancer Center, Gainesville, FL, USA; Division of Hematology/Oncology, University of Florida College of Medicine, Gainesville, FL, USA; University of Florida Health Cancer Center, Gainesville, FL, USA; Department of Anatomy and Cell Biology, University of Florida College of Medicine, Gainesville, FL, USA; University of Florida Health Cancer Center, Gainesville, FL, USA; Department of Anatomy and Cell Biology, University of Florida College of Medicine, Gainesville, FL, USA; University of Florida Health Cancer Center, Gainesville, FL, USA

## Abstract

Incorporation of histone variant H3.3 comprises active territories of chromatin. Exploring the function of H3.3 in prostate cancer (PC), we found that knockout (KO) of H3.3 chaperone HIRA suppresses PC growth *in vitro* and in xenograft settings, deregulates androgen-induced gene expression and alters androgen receptor (AR) binding within enhancers of target genes. H3.3 affects transcription in multiple ways, including activation of p300 by phosphorylated H3.3 at Ser-31 (H3.3S31Ph), which results in H3K27 acetylation (H3K27Ac) at enhancers. In turn, H3K27Ac recruits bromodomain protein BRD4 for enhancer-promoter interaction and transcription activation. We observed that HIRA KO reduces H3.3 incorporation, diminishes H3.3S31Ph and H3K27Ac, modifies recruitment of BRD4. These results suggest that H3.3-enriched enhancer chromatin serves as a platform for H3K27Ac-mediated BRD4 recruitment, which interacts with and retains AR at enhancers, resulting in transcription reprogramming. In addition, HIRA KO deregulates glucocorticoid- (GR) driven transcription of genes co-regulated by AR and GR, suggesting a common H3.3/HIRA-dependent mechanism of nuclear receptors function. Expression of HIRA complex proteins is increased in PC compared with normal prostate tissue, especially in high-risk PC groups, and is associated with a negative prognosis. Collectively, our results demonstrate function of HIRA-dependent H3.3 pathway in regulation of nuclear receptors activity.

## Graphical Abstract

**Graphical Abstract:**
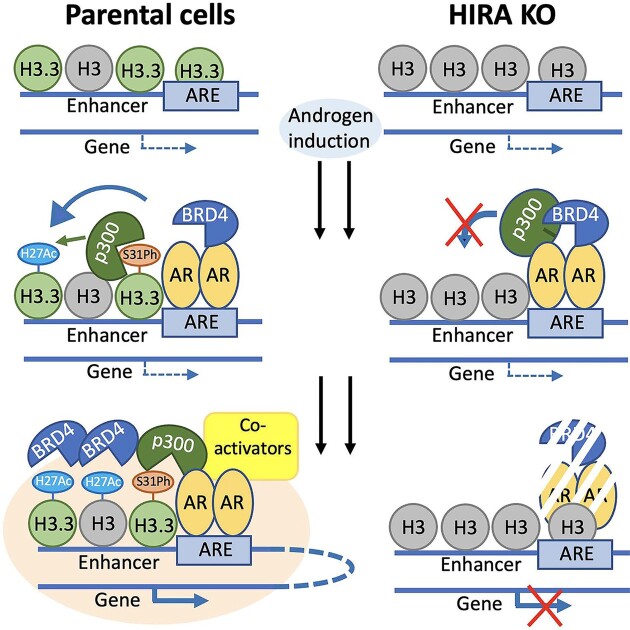

## INTRODUCTION

Prostate cancer (PC) is the second leading cause of cancer mortality in American men (1). PC that relapses after hormonal therapies (castration-resistant PC; CRPC (2)) is the cause of almost all PC-related deaths. Pathologic growth of the prostate is controlled mainly by steroid androgens. Recently diagnosed (both local and metastatic) diseases are treated with androgen ablation therapies that suppress androgen production or block androgen binding to AR ligand-binding domain (LBD) (3), thereby preventing nuclear translocation of AR and its binding to AR Response Elements (AREs). These therapies are effective in majority of patients, yet offer only a temporary relief, and the disease eventually recurs as treatment-resistant CRPC that is characterized by either AR loss-of function (treatment-emergent neuroendocrine PC, t-NEPC (4,5)) or gain-of-function, expressing AR variants (AR-Vs) with deleted LBD (AR-DLBD) (6). Identification of additional mechanisms involved in the transition of androgen-dependent PC to CRPC presents an opportunity to improve disease diagnosis and outcome. Hence, identification of co-regulators of AR may yield targets (and drugs) for effective and sustained management of CRPC (7).

Initiation and progression of PC is determined by, among other factors, transcription reprogramming that may be controlled by epigenetic dysregulation. The main chromatin unit, the nucleosome, can be modulated by post-translational modifications of histones, and by the incorporation of histone variants that together determine transcription activity of chromatin. There are three variants of histone H3: H3.1, H3.2 and H3.3 (8). H3.1 and H3.2 are the predominant forms (9,10) and H3.3 differs from H3.1 by only five amino acids. While canonical H3.1/2 are incorporated into chromatin in S-phase, H3.3 is deposited through interphase, and is referred to as ‘replication-independent histone’ (11,12). Histone variants are deposited by specific chaperones. H3.3 is chaperoned by HUCA (HIRA, UBN1, CABIN1, ASF1a) (13) and Daxx/ATRX complexes (14,15), which deposit H3.3 at enhancers, transcription start sites (TSS), centromeres and telomeres (14–17). Chromatin enriched in H3.3 is associated with elevated transcription activity (18). H3.3-containing nucleosomes are less stable compared to nucleosomes with canonical histones, thereby providing DNA accessibility to the transcription machinery at regulatory elements (19). These observations prompted Hake and Allis to propose the ‘histone variant barcoding’ hypothesis, stating that ‘histone H3 variants exhibit distinct posttranslational ‘signatures’ that influence epigenetic states during differentiation and development’ (9). Concordant with this model, H3.3 deposition coincides with H3K4me3, a mark for transcriptionally active promoters (9,14,20,21); H3.3-enriched chromatin is low with silencing marks (22,23). The role of H3.3 pathways in cancer was recently proposed by the discovery of H3.3 mutations (termed ‘oncohistones’(24–26)) in several malignancies. Multiple lines of evidence pointed to the essential function of H3.3 deposition pathways in initiation and progression of pancreatic and brain tumors (reviewed in (27–30)) and recent studies revealed its role in metastatic breast cancer (31).

H3.3 is enriched at enhancers (32), and recent findings implicated H3.3 in the activation of histone acetyltransferase p300 resulting in acetylation of H3K27 (H3K27Ac) at enhancers (33). This function required phosphorylation of H3.3S31 (H3.3S31Ph), a unique H3.3 residue, by checkpoint kinase CHK1(33,34) and by the main activator of the inflammatory transcription factor NF-κB, kinase IKKα (35). AR binds to AREs in majority of active enhancers and super-enhancers (SE) (36) in distal intergenic or intronic regions of target genes. Together, these data suggest a function of H3.3 in the AR-driven transcription in PC. Investigating function of H3.3 pathway in PC, we observed that HIRA KO strikingly decreases androgen induced transcription. Evaluating enhancers associated with androgen-target genes, we found that HIRA KO reduces H3.3 incorporation, diminishes H3K27Ac and H3.3S31Ph. In addition, it alters dynamics of BRD4 and AR binding at enhancers, first elevating and then reducing levels of these proteins at AREs. These data suggest that H3.3-enriched enhancer chromatin serves as a platform for AR-dependent and H3K27Ac-mediated recruitment of BRD4 to enhancers, implying two-steps in the assembly of transcription complex for AR-driven transcription reprogramming. In addition, HIRA KO deregulates glucocorticoid-driven transcription, suggesting a common H3.3/HIRA-dependent mechanism of nuclear receptors function. Collectively, our data suggest a function of HIRA-dependent H3.3 pathway in PC progression.

## MATERIALS AND METHODS

### Cell culture

Isogenic R1-AD1 (AR-WT) and R1-D567 (ARDLBD) cells (37) (received from Dr Scott Dehm, University of Minnesota, Minneapolis, MN) were cultured in RPMI 1640 medium (Corning, # 10040CV) supplemented with 10% fetal bovine serum (Gibco, #10437036) and 100 U/ml penicillin and 100 μg/ml streptomycin (Gibco BRL, Carlsbad, CA) and grown at 37°C in a humidified 5% CO_2_ incubator. 72 h before experiment cells were transferred to RPMI 1640 phenol-free media (Gibco # 11835030), supplemented with 10% charcoal stripped fetal bovine serum (CSS, Gibco, # 12676029), and 100 U/ml penicillin and 100 μg/ml streptomycin. Synthetic androgen R1881 (Sigma-Aldrich, # R0908) was prepared according to manufacturer recommendations and used in 1 nM concentration. JQ1 (Sigma-Aldrich, # SML1524) was prepared according to manufacturer recommendations and used in concentrations as outlined in specific experiments.

### Antibodies

The following antibodies were used in this study: HA mouse monoclonal antibody (16B12) (BioLegend, # 901502), FLAG M2 mouse monoclonal antibody (Sigma, # F1804), AR rabbit polyclonal antibody (Millipore, # 06–680) for ChIP, AR N-20 rabbit polyclonal antibody (Santa Cruz, # sc-816) for Western blotting, FKBP5 rabbit polyclonal antibody (Cell Signaling, #8245), H3K4Me1 rabbit polyclonal antibody (EpiCypher, # 13–0040), H3K27Ac mouse monoclonal antibody (EpiCypher, # 13-0045), HIRA(38), Daxx rabbit polyclonal antibody (39); BRD4 rabbit polyclonal antibody (Fortis Life Science, # A301-985A100) for Western blotting and mab BL-149-2H5 for ChIP, GR (G-5) mouse monoclonal antibody (Santa Cruz, # sc-393232), H3.3S31Ph rabbit monoclonal antibody (Abcam, # ab92628), Actin (AC-74) mouse monoclonal antibody (Sigma, # A5316).

### Production of FLAG-HA tagged H3F3A cells

Two oligos H3F3AN-1–73F CACCGTCAATGCTGGTAGGTAAGTA and H3F3AN-1-73R AAACTACTTACCTACCAGCATTGAC containing gRNA sequence were annealed and inserted into pSpCas9-2A-Puro vector (PX459, Addgene # 62988), digested with BbsI-HF enzyme (NEB, # R3539S). DNA sequence, 1 KB upstream and downstream from the start codon of the H3F3A gene, was cloned into pUC19 backbone, then sequence coding FLAG-HA tag was introduced into 5′-end of H3.3 ORF by Gibson assembly. Two nucleotide's substitutions were made in sequence recognized by gRNA to prevent repeated targeting of the modified allele. R1-AD1 and R1-D567 cells were transfected with equimolar amount of pSpCas9-2A-Puro H3F3A-1-73 plasmid and pUC19-FLAG-HA-H3F3A plasmid using Lipofectamine 2000 (Invitrogen). After 3 days transfected cells were trypsinized, counted and placed into 96-well plates, ∼1 cell/well, for clonal growth. Three weeks later each 96-well plate was duplicated, and clones were analyzed for insertion of FLAG-HA tag by Restriction Fragment Length Polymorphism and immunofluorescence staining. Correct insertion of FLAG-HA tag was confirmed by sequencing (MGH CCIB DNA Core, Cambridge).

### Production of AR, HIRA and daxx knockout (KO) cell lines.

The Alt-R CRISPR-Cas9 System (Integrated DNA Technologies) was used to produce KO cells. The following protospacer sequences were use in crRNA design for knockout of AR, HIRA and Daxx genes: CTGGGACGCAACCTCTCTCG, CTGGCTAGCCTCATGCAGCG and GCAGACAGCAGACCACCCTG accordingly. crRNA was annealed with tracrRNA and mixed with Cas9 enzyme (Integrated DNA Technologies)). R1-AD1-FLAG-HA-H3F3A cells were electroporated using Neon Transfection system (Invitrogen). After 3–5 days cells were trypsinized, counted and placed into 96-well plates, ∼1 cell/well, for clonal growth. Knock-out of target genes was confirmed immunofluorescence, Western blotting and by DNA sequencing.

### Production of stable knock-down cell lines and siRNA transfection.

pLKO.1-puro shHIRA lentiviruses (31) were produced by co-transfection of 293T cells with p-VSV-G and pCMVΔ8.2 plasmids. Lentivirus was harvested 36, 48 and 60 h after transfection. Medium was filtered through 0.45 μm SFCA filter (Corning, # 431220) and used for infection in the presence of 10 ug/ml polybrene (Sigma Aldrich, # H9268) to produce stable HIRA silencing in R1-AD and R1-D567 cell lines. Selection of resistant cells that express shHIRA was done with 4 μm/ml puromycin.

To induce transient silencing of HIRA, cells were transfected with two rounds of HIRA siGENOME SMARTpool (Dharmacon, # M-013610–01-0020) and DharmaFECT 1 transfection reagent (Dharmacon, # T-2001-01). Cells transfected with siGENOME Non-Targeting siRNA Pool (D-001206-13-20) were used as control.

### RNA-seq

1 × 10^6^ cells were plated on 6 cm dish in RPMI 1640 medium, sans phenol-red (Gibco, #11835-030), 10% CSS (Invitrogen), 1% penicillin/streptomycin solution (Corning, # 30-002-CI) and grown for 72 h. Cells were treated with 1 nM R1881 for 4, 12 and 24 h. RNA was isolated using RNeasy Plus Kit (QIAGENE, # 74134) according to the manual and sequenced at Novogene.

### ChIP-seq

ChIP-seq protocol (40) with some modification was used. 10 × 10^6^ cells were fixed with 1% formaldehyde in PBS for 10 min at room temperature, quenched with 1.25 M glycine to a final concentration of 0.125 M for 5 min. Cells were washed twice with ice-cold PBS, scraped into 6 ml Farnham lysis buffer (5 mM PIPES pH 8.0, 85 mM KCl, 0.5% Igepal CA-630) with protease inhibitors (ThermoFisher, # A32953), transferred to 15 ml Falcon tubes, and centrifuged at 800 rcf for 5 min at 4 °C. The pellet was resuspended in 1 ml fresh Farnham lysis buffer and incubated on ice for 10 min. Nuclei were collected by centrifugation at 500 rcf, 4°C for 5 min, resuspended in 150 μl cold RIPA buffer (1× PBS (Sigma, # D8662), 1% Igepal CA-630, 0.5% sodium deoxycholate), 0.5% SDS and transferred to TPX tube (Diagenode). Chromatin was sheared by Diagenode Bioraptor Pico with peak enrichment at 200 bp. After sonication SDS concentration was adjusted to 0.1% SDS with RIPA buffer, and chromatin was spin at maximum speed in a microfuge for 15 min at 4°C. The supernatant was transferred into new tube and snap frozen in liquid nitrogen until needed. Chromatin was pre-cleared with Pierce Protein A/G Magnetics beads (Pierce # 88802). 2–5 μg primary antibody were added per 5 × 10^6^ cells and incubated overnight at 4°C with rotation. Next day 25 μl Pierce Protein A/G Magnetic beads (pre-blocked with PBS/BSA), were added, and incubated for 2 h at 4°C with rotation. Beads were washed 5 times with 1 ml wash buffer (100 mM Tris–HCl, pH 7.5, 500 mM LiCl, 1% Igepal CA-630, 1% sodium deoxycholate) and 1 time with 1 ml TE buffer (10 mM Tris–HCl pH 7.5, 0.1 mM EDTA), with 3 min rotation in-between for each wash. Chromatin was eluted from beads with 150 μl fresh prepared IP elution buffer (1% SDS, 0.1 M NaHCO_3_) and de-crosslinked overnight at 65°C. DNA was purified using Monarch PCR/DNA Cleanup Kit (New England BioLabs). ChIP-seq libraries were prepared using NEBNext Ultra II DNA Library Prep Kit for Illumina (New England BioLabs) and sequenced on the Illumina NovaSeq 6000 Sequencer (2 × 150) at the University of Florida ICBR.

### ATAC-seq

ATAC-seq libraries were prepared using ATAC-seq kit from Active Motif (Active Motif, CA, # 53150) according to the manual and sequenced on the Illumina NovaSeq 6000 Sequencer (2 × 150) at the University of Florida ICBR.

### Bioinformatic analysis

**RNA-Seq:** Short reads were mapped to the GRCh38 reference transcriptome using STAR (41). Quantification produced tables of FPKM values for each gene in each sample. Differential analysis was performed with DESeq2 (42), generating tables of significantly over- or under-expressed genes in each contrast.

**ChIP-Seq and ATAC-Seq:** Short reads were mapped to the GRCh38 reference genome using Bowtie2 (43). Peak calling was performed with MACS (44) using parameters appropriate for the type of signal being detected. Super-enhancers were identified using ROSE version 0.1 (45). All interval operations were performed with bedtools (https://bedtools.readthedocs.io/en/latest/), and plots were generated with custom scripts. Differential analysis of ChIP-Seq and ATAC-Seq peaks was performed with DASA (https://github.com/uf-icbr-bioinformatics/dasa).

**Profile plots and boxplots**: To generate the profile plot for a given signal in a set of regions, we extracted the normalized coverage values from the short-read alignment file for that signal for each region, and we computed the geometric average of the results to reduce the impact of outliers. Profiles for the different cell lines or timepoints were then superimposed in the same chart. A similar process was used to generate metagene profile plots, in which all gene regions were scaled to the same length, and a fixed flanking region of 1 kb was added upstream and downstream. Boxplots were generated based on the total coverage in each region. All processing was performed using custom scripts.

### Statistical analysis

Statistical analysis was performed with GraphPad Prism 9.2.0 (GraphPad Software, Inc., San Diego, CA). Homogeneity of variance was estimated using Brown–Forsythe test. The comparison of means between different groups was performed by one-way ANOVA with either Tukey or Dunnett multiple comparison test correction.

### Western blotting analysis

Protein samples were separated on 4–20% Mini-Protean TGX gel (Bio-Rad, #4561096) and transferred to nitrocellulose membrane using iBlot 2 system (Invitrogen, Thermo Fisher Scientific). Membranes were blocked with 5% nonfat milk/PBS, 0.1% Tween (PBST). Primary antibodies were diluted in 5% milk/PBST and incubated overnight at 4°C. Membranes were washed two times with PBST and incubated for 1 h at room temperature with appropriate IRDye secondary antibody (Li-COR Biosciences). Membranes were washed three times with PBST and visualized by Odyssey CLx Imaging System (Li-COR Biosciences).

### Colony formation assay

5 × 10^3^ cells were seeded in 12-well plates in complete media, cultured for 7 days, fixed for 10 min with 4% formaldehyde and stained with crystal violet (0.5%). Images were acquired with Epson photo scanner and area of colonies was calculated using ImageJ software. Experiments were repeated at least three times.

### Proliferation assay

Cells (5000 cell/well, 96-well plates) were set in 100 μl of RPMI 1640 phenol-free media supplemented with 10% CSS; 10 μl Alamar® blue reagent (Thermo Scientific # 00-100) was added to each well. Data were collected using Spectra Max M3 plate reader after 4 h of signal development.

### Xenograft experiments

Soft collagen pellets containing parental R1-AD1, AR KO, Daxx KO or HIRA KO cells were implanted subcutaneously to establish xenografts in 6 weeks old male Hsd:Athymic Nude-Foxn1nu mice. After 4 weeks, tumor growth was analyzed as we described before (46).

### Immunofluorescence

For characterization of H3.3 in cells, immunofluorescence was done as described (47). Briefly, 75 × 10^4^ cells were plated on microscope coverslip glass (Fisher Scientific) in RPMI1640/ 10% FBS media. Сells were fixed with 1% formaldehyde for 10 min, permeabilized with 0.5% Triton X-100, and incubated with HA antibodies for 1 h at room temperature. After two washes with PBS, cells were incubated with secondary antibodies conjugated with Alexa Fluor 488 or 594 dye (Invitrogen). DNA was stained with Hoechst 33342 (Sigma). Images were analyzed using either Leica DMI4000 B fluorescent microscope or Leica TCS SP5 confocal microscope.

## RESULTS

**Characterization of CRPC cell models**. H3.3 and H3.1/2 differ by only 5 amino acids, and 4 of these are in the globular part of histone (and, therefore, are not exposed in the context of nucleosome/chromatin) thus making it difficult to produce H3.3-specific ChIP-grade antibody. To overcome this challenge, we used CRISPR-Cas9 genome editing to knock-in FLAG-HA-epitope tags at 5′ of H3F3A gene, thereby producing R1-AD1 (AR-WT) cells (37) expressing endogenous FLAG-HA-H3.3. Cells were characterized by sequencing, immunofluorescence (IF), and Western blot (Supplementary Figure S1A, B). Using CRISPR/Cas9 genome editing on FLAG-HA-H3.3 R1-AD1 cells, we produced AR, HIRA and Daxx knockout (KO) cells. At least three subclones were characterized for each KO by sequencing, IF, and Western blot (Supplementary Figure S1B). Deletion of AR, HIRA or Daxx did not affect levels of remaining two proteins. HIRA-KO reduced H3.3 levels, potentially by a negative compensatory mechanism to reduce toxic effect of unincorporated histone H3.3 in the absence of chaperone HIRA. Thus, we created and characterized unique cell models to selectively test the function of endogenous H3.3 and its pathway in PC.

**Figure 1.**
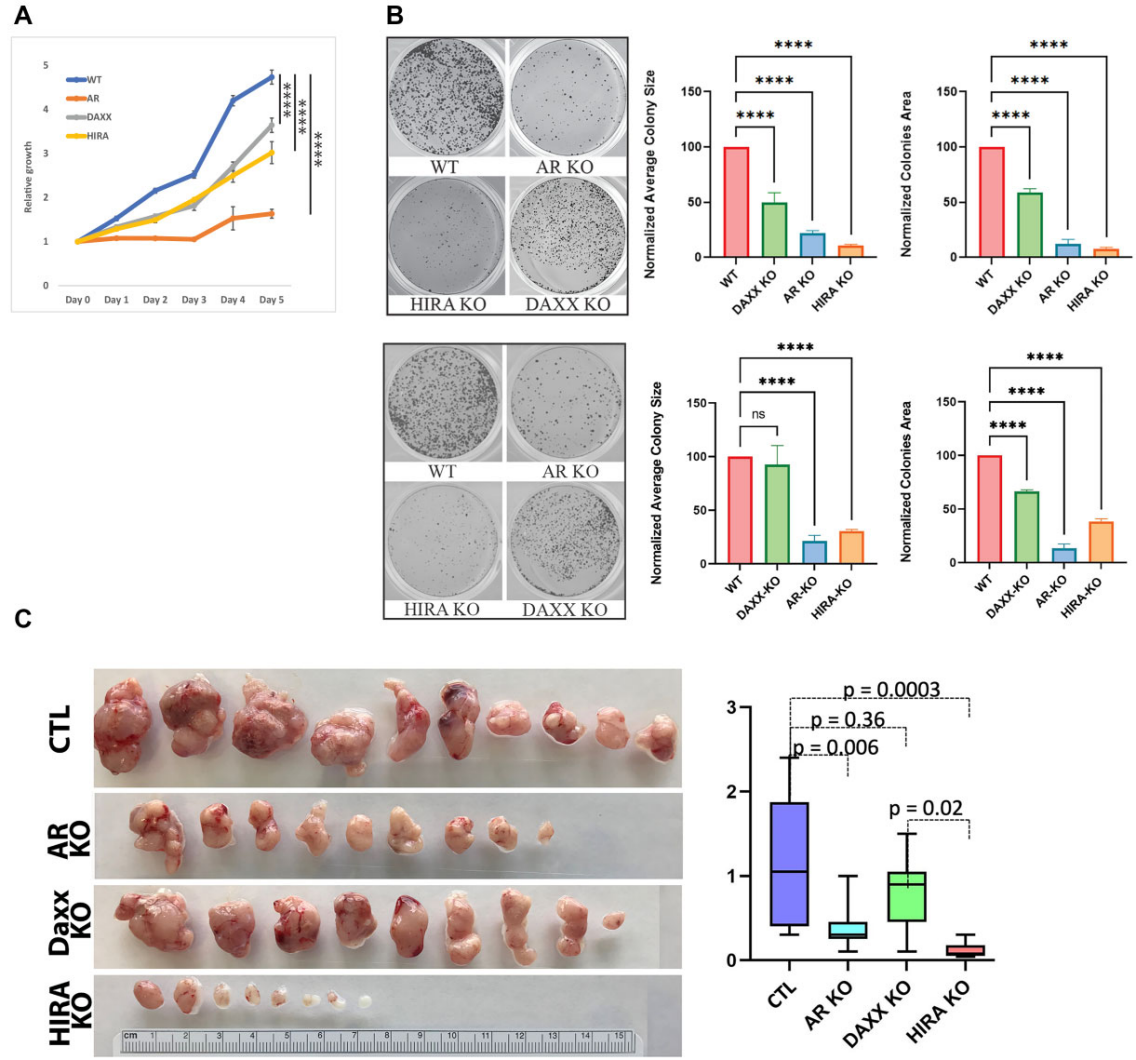
Modification of HIRA affects proliferation of PC cells. (**A**) Proliferation of R1-AD1 and Daxx KO, AR KO, HIRA KO cell lines. (**B**) colony formation assays with these cell lines in androgen deprived (top) and R1881 stimulated conditions (bottom). Left: representative images; middle: average colony size; right: combined colonies area calculated with ImageJ. *P*-values: **** < 0.0001. (**C**) Modification of HIRA affects xenograft growth of PC cells. 10^6^ R1-AD1 cells (CTL), Daxx KO, AR KO and HIRA KO cells were implanted subcutaneously in athymic nude mice, 10 per group. 4 weeks later, tumors were collected and photographed (left). Tumor weight in g (right).

**HIRA affects PC cell proliferation**. Using KO cells, we tested effect of manipulating H3.3 chaperones levels on cell proliferation. KO of AR or HIRA reduced the R1-AD1 cell proliferation rate and colony formation (Figure 1A, B). Daxx KO had a reproducible yet lower effect on cell growth. These data establish HIRA function in proliferation of PC cells.

To further address the function of HIRA in PC, effect of manipulated HIRA expression was analyzed *in vivo*. Soft collagen pellets containing parental R1-AD1, and cells with KO of AR, Daxx or HIRA were implanted subcutaneously to establish xenografts in 6 weeks old male Hsd:Athymic Nude-Foxn1nu mice. After 4 weeks, tumor growth was analyzed as we described before (46). Both AR and HIRA KO significantly reduced the tumor size (Figure 1C). Daxx KO reproducibly decreased the tumor size, albeit to a lesser degree.

**Figure 2.**
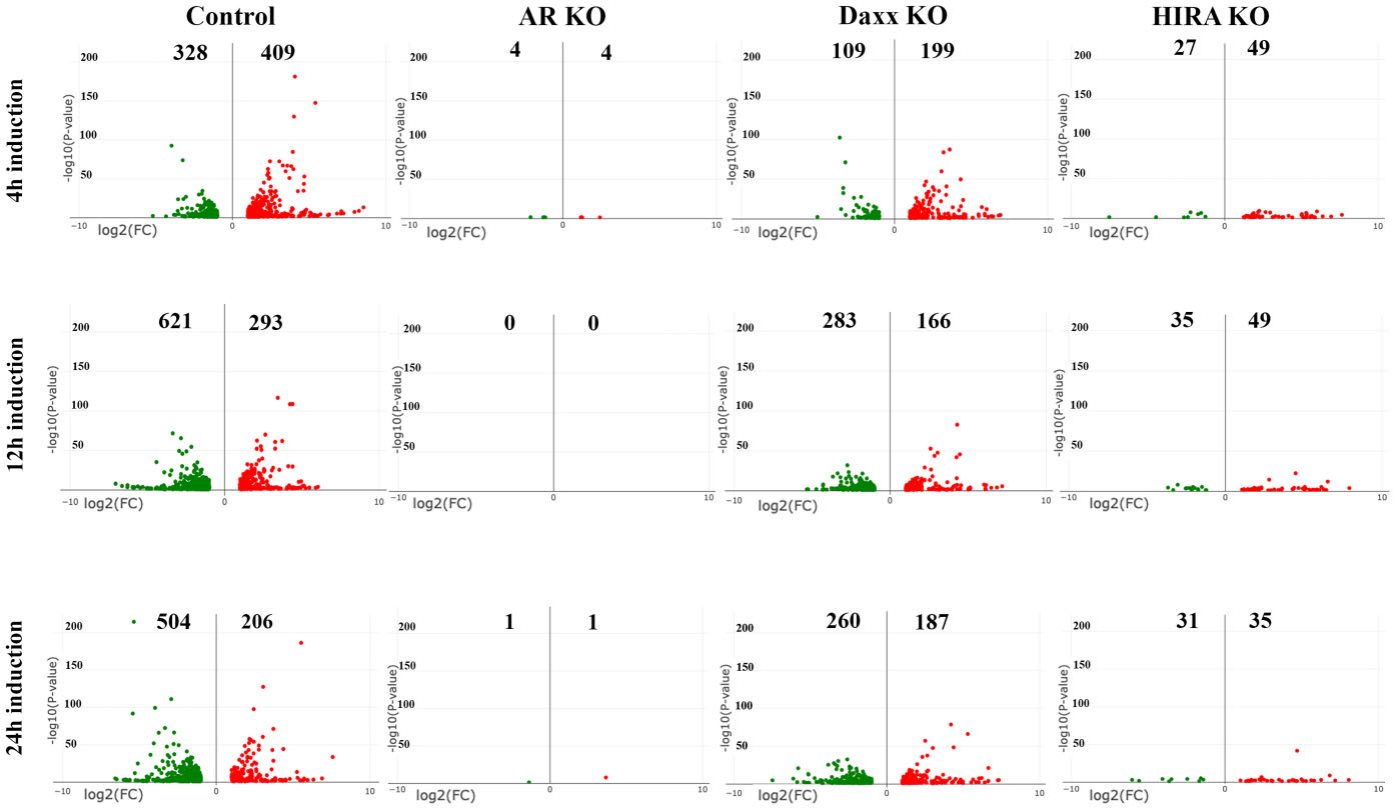
Differential gene expression after androgen stimulation. Expression analysis (RNA-seq, volcano plot) of R1-AD1 cells, parental (Control), AR KO, Daxx KO and HIRA KO. Numbers correspond to genes that are at least 2-fold (*P*-adjusted < 0.05) up- (red) or downregulated (green) by 4 h (top row), 12 h (middle row) or 24 h (bottom row) of R1881 stimulation compared with androgen-deprived (72 h) in parental cells. X: expression, log_2_; Y: *P*-value, log_10_. See Supplementary Figure S2 for Venn diagrams and regulated pathways analysis.

**HIRA affects androgen-induced gene transcription**. To address mechanisms of HIRA-dependent effect on cell proliferation, we evaluated potential influence of HIRA on androgen-dependent transcription activity using RNA-seq analysis. RNA was isolated from R1-AD1 cells (parental cells) and individual AR, HIRA and Daxx KO clones under androgen-deprived (72 h) and androgen (R1881) stimulated (4, 12, 24 h) conditions. Genes with *P*-value <0.05 (adjusted using the Benjamini and Hochberg's approach for controlling the False Discovery Rate (FDR)) found by DESeq2 were considered as differentially expressed. Expression analysis is presented in Figure 2; numbers correspond to genes that are at least 2-fold (*P*-adjusted < 0.05) up- or down-regulated following R1881 stimulation in comparison to androgen-deprived parental cells. Using these parameters, we found that 409 genes were up- and 328 down-regulated at 4 h in parental cells. AR KO abolished this regulation (with only 4 up- and 4 downregulated genes remaining), confirming AR-dependence. Both Daxx and HIRA KO affected androgen-stimulated gene expression, although HIRA KO impacted androgen-dependent gene expression more strongly than Daxx KO, as evident by reduction of upregulated genes to 49 and downregulated genes to 27 (Figure 2; Supplementary Figure S2A for Venn diagram and Supplementary Table S1 for list of genes with androgen-regulated expression affected by both AR and HIRA KO). Using the KEGG database, we identified several androgen-regulated pathways that are affected by both AR and HIRA KO (Supplementary Figure S2B and Supplementary Table S2), including cancer- and metastasis-associated pathways, as FoxO (48), TNF (49), TGF-beta (50), PI3K-Akt (48), MAPK (51) and Wnt (52). Similar results were observed at 12 and 24 h of treatment (Figure 2). We conclude that HIRA is required for androgen-regulated transcription.

**Figure 3.**
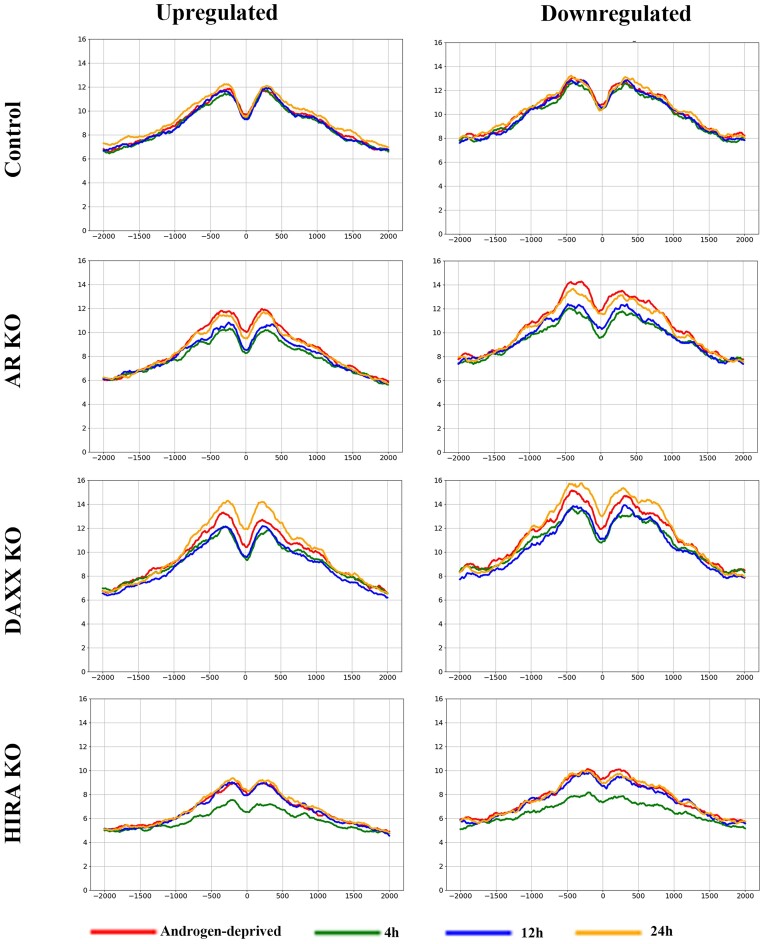
H3.3 association with TSS of androgen-regulated genes; analysis within cell lines. ChIP-seq profiles of H3.3 (endogenous HA-H3.3) at the 409 androgen-up- (left) and 328 androgen-downregulated (right) genes in R1-AD1 parental (Control), AR KO, Daxx KO and HIRA KO cells in the androgen-deprived (0h) and androgen-induced conditions (at 4, 12, 24 h). 0: TSS. See Supplementary Figure S3 for time points comparison.

**Figure 4.**
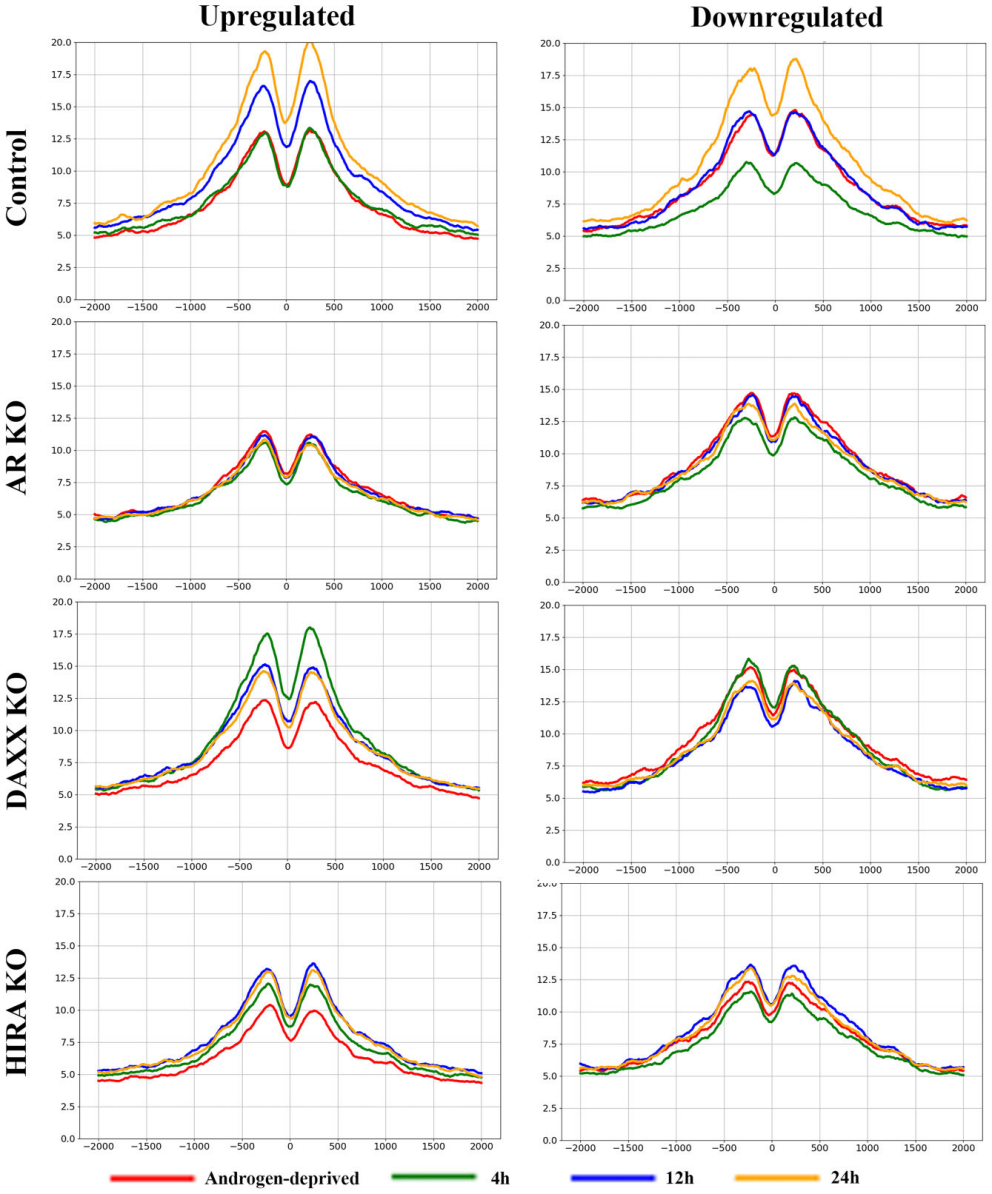
H3K27Ac association with TSS of androgen-regulated genes; analysis within cell lines. ChIP-seq profiles of H3K27Ac at the 409 androgen-up- (left) and 328 androgen-downregulated (right) genes in R1-AD1 parental (Control), AR KO, Daxx KO and HIRA KO cells in the androgen-deprived (0 h) and androgen-induced conditions (at 4, 12, 24 h). 0: TSS. See Supplementary Figure S5 for time points comparison.

**HIRA KO reduces levels of H3.3 and H3K27Ac at up- and downregulated genes**. To address mechanism of transcription deregulation in KO cells, we performed analysis of H3.3 (endogenous FLAG-HA-H3.3 with anti-HA antibody) and transcription-associated marker H3K27Ac at the transcription start sites (TSSs), gene bodies (GB), and transcription end sites (TES) focusing on 409 up- and 328 down-regulated genes (0 h versus 4 h R1881 stimulation, parental cells, Figure 2). We compared ChIP-seq profiles in R1-AD1 parental (Control), AR, Daxx and HIRA KO cells under androgen-deprived (0 h) and androgen-induced conditions (at 4, 12, 24 h; levels of H3.3 were monitored by Western blot analysis, Supplementary Figure S1C). Compared with parental cells, AR KO reduced H3.3 levels at all three elements (TSS, GB, TES) at up-regulated genes during androgen induction, and at down-regulated genes in androgen-deprived conditions (Figure 3, Supplementary Figure S3 for TSS; in this and follow-up ChIP-seq analyses comparison was made between cell lines at the same time points and between time points within each cell line and Supplementary Figure S4 for entire genes). Reduction of H3.3 in AR KO cells was concordant with a transcription-associated H3.3 deposition mechanism (53–55) that is abrogated at androgen-regulated genes in these cells. HIRA KO reduced H3.3 levels in up- and down-regulated genes at all three elements (TSS, GB, TES) at all time points, confirming function of this chaperone in H3.3 loading at coding regions (14,56). Accumulation of H3.3 in Daxx KO cells at 24 h can be explained by the increased availability of H3.3 to HIRA or other H3 chaperones such as CAF-1 (16) and NASP (57) for incorporation into gene coding element and is concordant with prior observations that Daxx KO reduces H3.3 at telomeres (15), centromeres and pericentromeres (58). In parental cells, accumulation of H3K27Ac was observed at TSS, gene bodies, and TES in up-regulated genes at 12 and 24 h (Figure 4, Supplementary Figure S5 for TSS and Supplementary Figure S6), while it was elevated only at 24 h (albeit to a lesser degree compared with up-regulated genes) at down-regulated genes. We observed temporary elevation of H3K27Ac in Daxx KO cells (at 4 h) that may be at least partly explained by a reduction of HDAC1/2 recruitment to chromatin, which is mediated by interaction with Daxx in parental cells (59,60). AR KO completely abolished, and HIRA KO strongly reduced H3K27Ac accumulation, in agreement with a recent publication (56).

**Figure 5.**
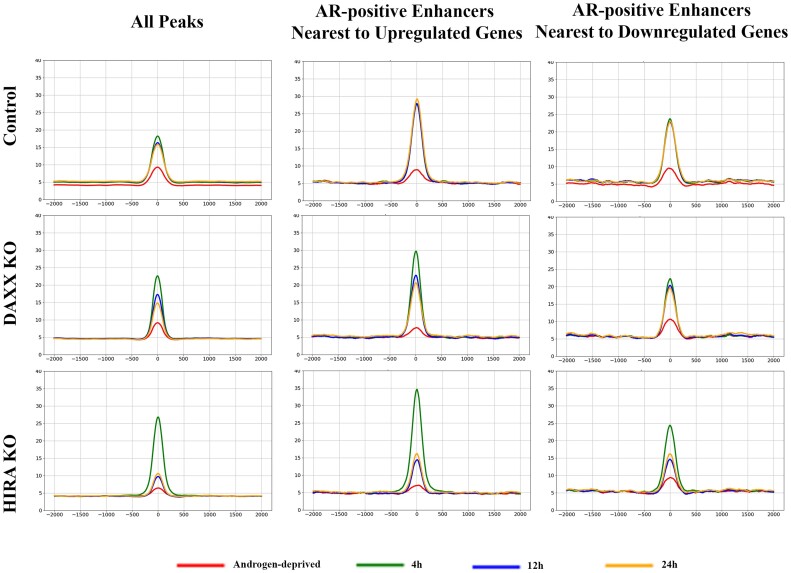
AR association with chromatin is regulated by HIRA. Left: Metaplot of AR peaks (position ‘0’ at 4 h in R1-AD1 parental cells) in R1-AD1 parental, HIRA KO and Daxx KO in androgen-deprived (72 h, red) and R1881 stimulated for 4 h (green), 12 h (blue), 24 h (yellow). In parental cells, AR association with chromatin is elevated at 4 h of stimulation and remains at the same levels at later time points. In HIRA KO, association is reduced in deprived condition, is much higher compared with parental at 4h, and next is substantially reduced at 12 and 24 h. Analysis of AR at enhancers nearest to the 409 up- (middle) and 328 downregulated (right) genes is similar to the overall AR behavior. See Supplementary Figure S7 for time points comparison.

**HIRA KO affects AR association with chromatin**. Massive deregulation of androgen-induced expression in HIRA KO cells (Figure 2, Supplementary Figure S2) suggested altered activity of AR. Hence, we analyzed AR association with chromatin by ChIP-seq in R1-AD1 parental and KO cells (AR KO cells were used as a negative control) under androgen deprived and R1881 stimulated conditions (4, 12, 24 h). At 4h after stimulation, we identified 5920, 5528 and 5412 AR peaks, corresponding to parental, Daxx and HIRA KO cells. In parental cells, AR association with chromatin was elevated at 4 h and remained mostly unchanged at 12 and 24 h (Figure 5, left and Supplementary Figure S7A left). We observed increased AR accumulation in Daxx KO cells at 4 h of stimulation that may be explained by the reported contribution of Daxx/AR interaction to the negative regulation of AR binding to DNA (61), which is abrogated in Daxx deleted cells. In HIRA KO cells, AR association is elevated at 4 h of stimulation, and is substantially reduced to almost pre-induced levels at 12 and 24 h.

AR binds to active enhancers (36). Therefore, we analyzed dynamics of AR at enhancers nearest to the same group of up- (409) and down-regulated (328) genes. To map enhancers, we performed ChIP-seq for enhancer marker H3K4me1 (62) (Supplementary Figure S8). We identified AR-positive enhancers (by AR ChIP-seq at 4h in parental cells) nearest to each up- or down-regulated gene at 4h (median distance to upregulated: 196 819 bp, and downregulated: 542 080 bp) in parental R1-AD1 cells. We observed that AR KO reduced levels of H3K4me1 at AR-positive enhancers while HIRA KO had a similar effect on both AR-positive and AR-negative elements (most obvious at those associated with up-regulated genes, Supplementary Figure S8), implying overlapping functions of AR and HIRA/H3.3 pathway in maintenance of lineage specific enhancers. Methyltransferases KMT2C (MLL3) and KMT2D (MLL4) monomethylate H3K4 (63), and a reported association between H3.3 and KMT2D identified by BioID (64), suggests recruitment of this methyltransferase to H3.3-enriched chromatin areas (as enhancers) for monomethylation of H3K4 and explains the reduction of H3K4me1 in HIRA KO cells. Dynamics of AR at enhancers were similar to the AR behavior at all peaks (Figure 5, middle: for enhancers associated with up- and Figure 5, right: down-regulated genes; Supplementary Figure S7 for time points comparison), with the highest AR amplitude at enhancers associated with up-regulated genes. HIRA KO elevated AR association with enhancers at 4h, and that was substantially reduced at 12 and 24 h. Thus, HIRA KO affected dynamics of AR chromatin binding, including enhancers associated with up- and down-regulated genes. We conclude that HIRA-dependent pathway is important for the proper retention of AR at AREs after androgen induction, potentially explaining changes in transcription deregulation observed in HIRA KO cells.

**Figure 6.**
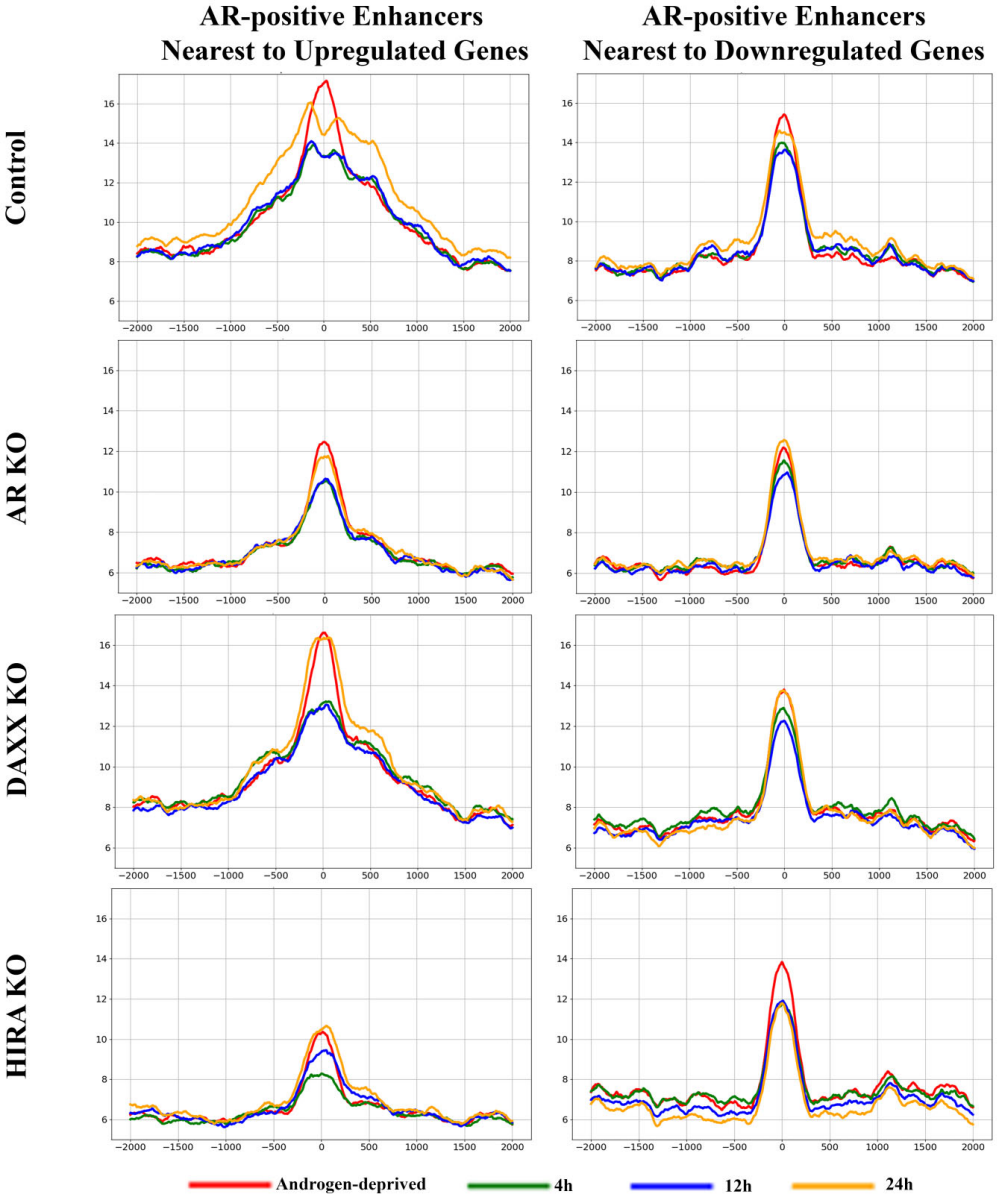
Dynamics of H3.3 at enhancers. Metaplot of H3.3 (endogenous HA-H3.3) at AR peaks (position ‘0’: AR at 4 h in R1-AD1 parental cells) at enhancers associated with up- (left) and down-regulated (right) genes in R1-AD1 parental (Control), AR KO, Daxx KO and HIRA KO in androgen-deprived (72 h, red) and R1881 stimulated for 4 h (green), 12 h (blue), 24 h (yellow). Levels of H3.3 at AR-positive enhancers associated with upregulated genes are elevated at 24 h of induction in parental and Daxx KO cells. H3.3 is reduced at enhancers in AR KO cells, suggesting AR function in maintenance of this transcription associated histone variant, and HIRA KO, confirming HIRA chaperone function. See Supplementary Figure S9 for time points comparison.

**HIRA KO affects epigenetics at AR-positive enhancers associated with AR-regulated genes**. H3.3 is enriched at enhancers (32), and we analyzed its association (endogenous HA–H3.3) with AR-positive enhancers nearest to genes up- and down-regulated by androgen in parental R1-AD1 cells (Figure 6 and Supplementary Figure S9 for time points comparison). In androgen deprived conditions, AR binding site (‘0’ at the graphs) is occupied by H3.3, suggesting H3.3-containing nucleosome accumulation at AREs in the absence of AR. H3.3 levels are reduced during androgen induction at these sites, most visible in the upregulated group, indicating nucleosome displacement by AR. Levels of H3.3 at the AR-positive enhancers associated with upregulated genes are elevated at 24 h of induction (in parental and in Daxx KO cells), confirming transcription-associated loading of H3.3 (53–55). In AR KO cells, H3.3 is reduced at the enhancers, consistent with H3.3 profiling at the regulated genes (Figure 3, Supplementary Figures S3, S4), suggesting role of the AR-regulated enhancer transcription in maintenance of H3.3 at enhancers. We observed a major reduction of H3.3 in HIRA KO, consistent with the chaperone function of HIRA at enhancers.

**Figure 7.**
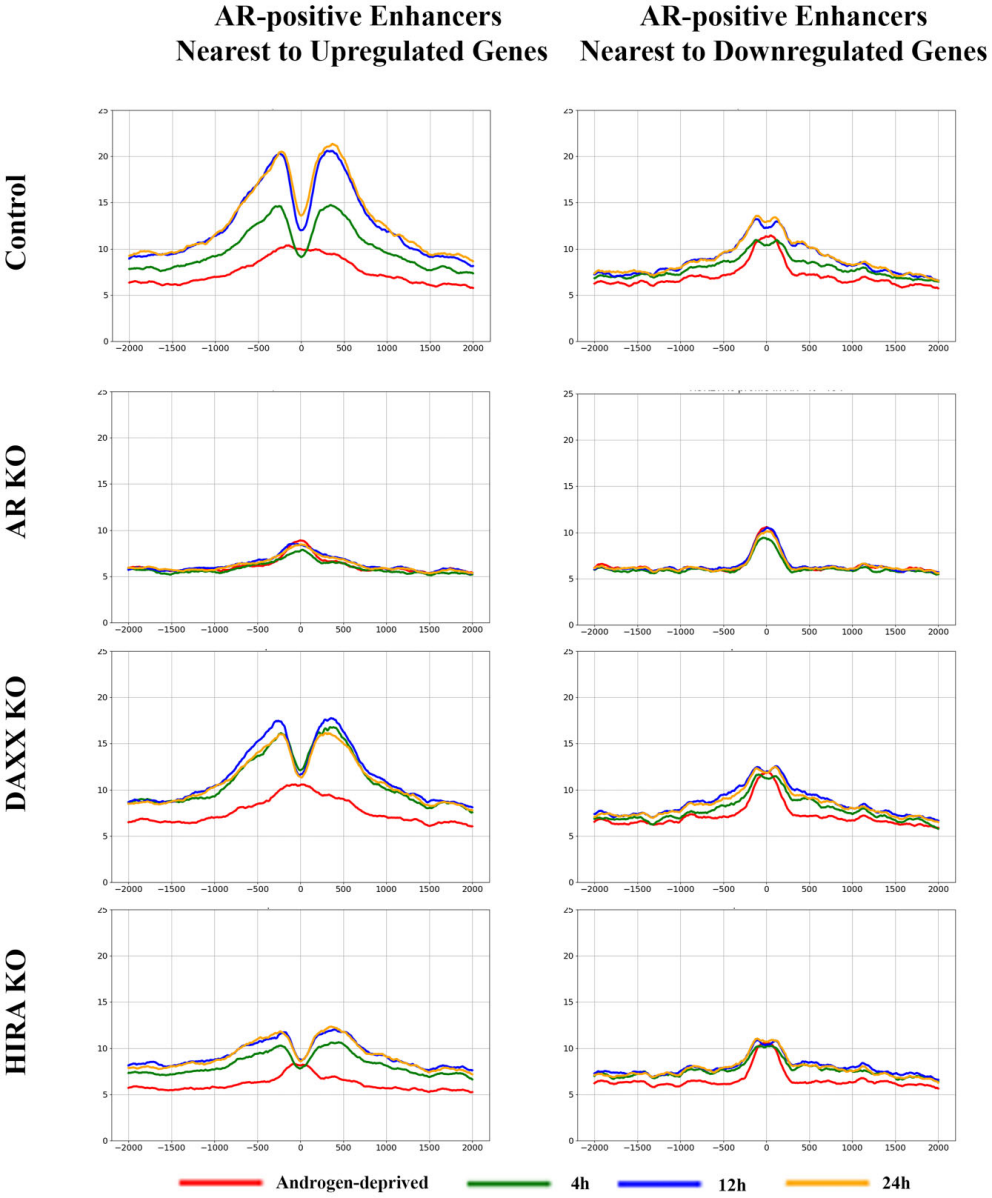
Dynamics of H3K27Ac at enhancers. Metaplot of H3K27Ac ChIP-seq analysis at AR peaks (position ‘0’: AR at 4 h in R1-AD1 parental cells) at enhancers associated with up- (left) and down-regulated (right) genes in R1-AD1 parental (Control), AR KO, Daxx KO and HIRA KO in androgen-deprived (72 h, red) and R1881 stimulated for 4 h (green), 12 h (blue), 24 h (yellow). In parental cells, H3K27Ac is gradually elevated during androgen treatment, to higher levels at enhancers associated with up-regulated genes. AR KO completely abolished, and HIRA KO strongly reduced accumulation of H3K27Ac at enhancers associated with both up- and downregulated genes. See Supplementary Figure S10 for time points comparison.

Next, we analyzed the marker of active enhancers H3K27Ac (62,65–67) at AR-positive enhancers nearest genes regulated by androgen. In parental and Daxx KO cells, H3K27Ac was gradually elevated during all induction timepoints of androgen treatment at enhancers associated with up-regulated genes (Figure 7 and Supplementary Figure S10 for time points comparison), consistent with the function of this modification at active enhancers. Similar tendency, albeit to a lesser degree, was observed at enhancers associated with down-regulated genes. Similar to H3.3, H3K27Ac was reduced at AR binding sites (‘0’ at the graphs) during androgen induction, most clear at enhancers associated with upregulated genes, suggesting nucleosome displacement by AR. Consistent with H3K27Ac profiling at regulated genes (Figure 4, Supplementary Figures S5, S6), we observed a temporary (at 4 h) increase of H3K27Ac in Daxx KO compared to parental cells. AR KO completely abolished, and HIRA KO strongly reduced, accumulation of H3K27Ac at enhancers associated with both up- and down-regulated genes (Figure 7 and Supplementary Figure S10).

**Figure 8.**
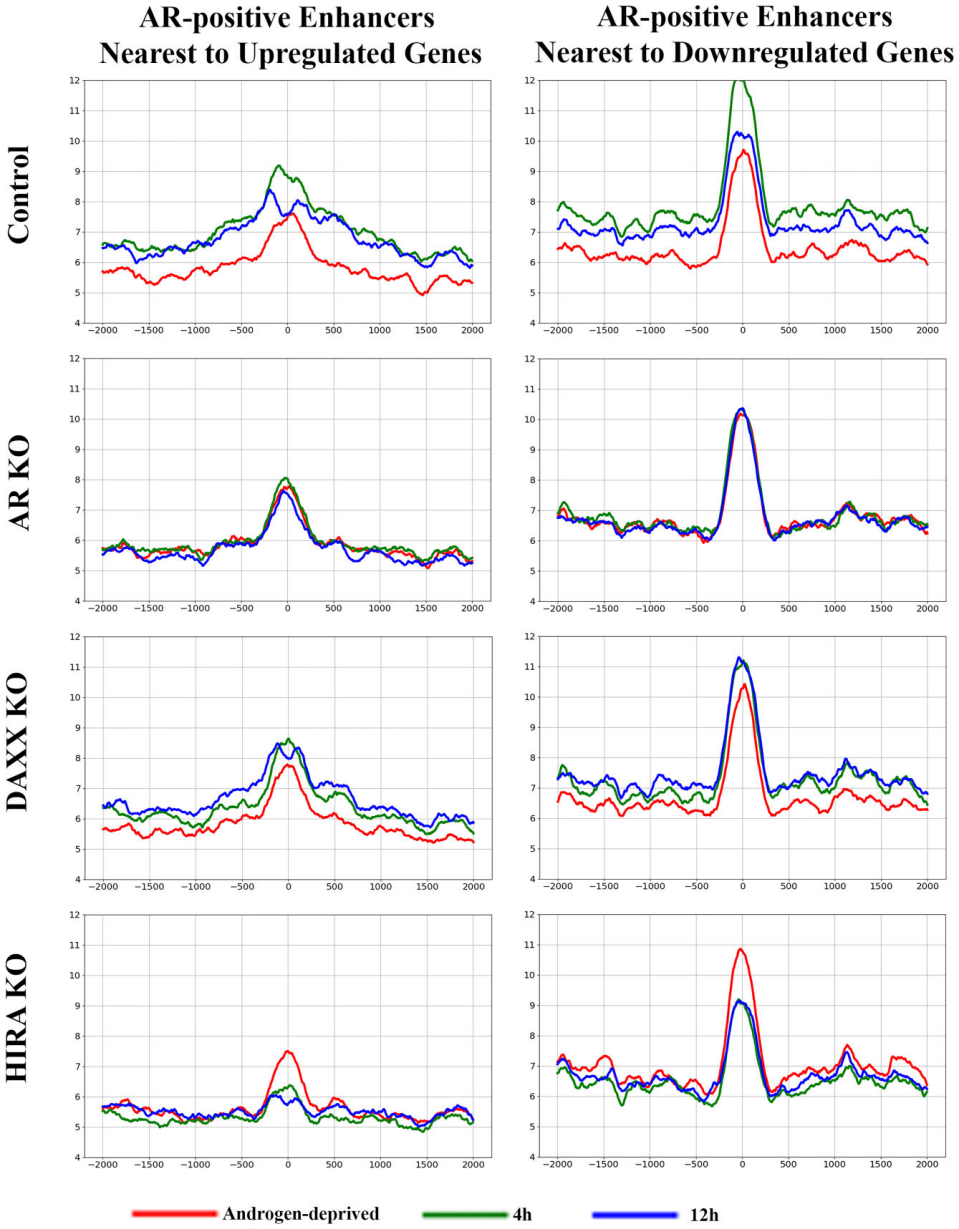
Dynamics of H3.3S31Ph at enhancers; analysis within cell lines. Metaplot of H3.3S31Ph ChIP-seq analysis at AR peaks (position ‘0’: AR at 4 h in R1-AD1 parental cells) at enhancers associated with up- (left) and down-regulated (right) genes in R1-AD1 parental (Control), AR KO, Daxx KO and HIRA KO in androgen-deprived (72 h, red) and R1881 stimulated for 4 h (green), 12 h (blue). Levels of H3.3S31Ph are androgen-induced in parental and Daxx KO cells, no changes in AR KO cells, and are reduced in HIRA KO cells, resembling H3.3 dynamics. See Supplementary Figure S11 for time points comparison.

Our results suggest H3.3 role in H3K27 acetylation at enhancers. Phosphorylation of H3.3S31 (H3.3S31Ph), a unique H3.3 residue, regulates activation of histone acetyltransferase p300 resulting in acetylation of H3K27 at enhancers (33). We observed androgen-induced increase of H3.3S31Ph in parental and Daxx KO cells, no changes in AR KO cells, and a reduction of H3.3S31Ph in HIRA KO cells (Figure 8 and Supplementary Figure S11 for time points comparison), that, together with H3.3 data, suggests function of this histone variant in acetylation of H3K27 at enhancers.

**Figure 9.**
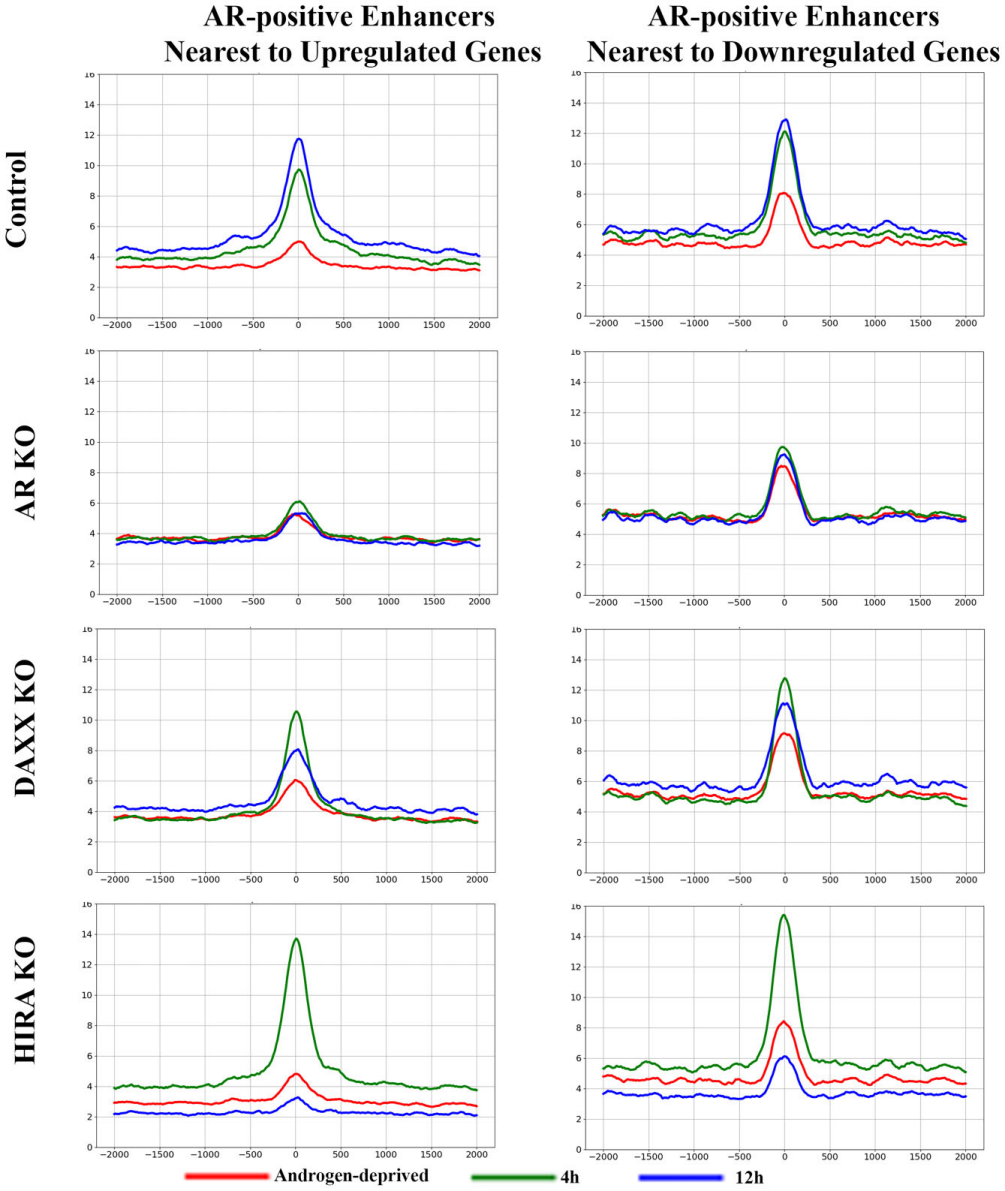
Dynamics of BRD4 at enhancers. Metaplot of BRD4 ChIP-seq analysis at AR peaks (position ‘0’: AR at 4 h in R1-AD1 parental cells) at enhancers associated with up- (left) and down-regulated (right) genes in R1-AD1 parental (Control), AR KO, Daxx KO and HIRA KO in androgen-deprived (72 h, red) and R1881 stimulated for 4 h (green), 12 h (blue). BRD4 accumulated within enhancers at AR binding sites in parental cells, with dynamics mirroring those of AR (Figure 2). AR KO abolished BRD4 accumulation. In HIRA KO cells, BRD4 dynamics is similar to AR, first accumulating at 4h of stimulation and next reducing at 12 h. See Supplementary Figure S12 for time points comparison.

**HIRA KO affects BRD4 binding to enhancers**. Epigenetic reader bromodomain protein BRD4 is associated with chromatin via interaction with acetylated histones, including H4K5, H4K8, H4K12, H3K9, H3K14, H4K16 and H3K27 (68–72); BRD4 is associated with H3K27Ac, marker of active enhancers(65–67), and is indispensable for enhancer activity (73). BRD4 acts as a scaffold protein recruiting transcription factors for transcription activation, including enhancer-promoter interaction (62,74). BRD4 interacts with AR (75) and is required for AR-mediated transcription. Inhibition of BRD4 abrogates AR binding to AREs(75) and enhancers enriched with H3K27Ac recruit BRD4 to chromatin (73). In parental and Daxx KO cells, androgen treatment induced BRD4 accumulation at AR binding sites within enhancers associated with up- and down-regulated genes, with dynamics mirroring those of H3K27Ac and AR (Figure 9 and Supplementary Figure S12 for time points comparison; levels of BRD4 were monitored by Western blot analysis, Supplementary Figure S1C). Increased BRD4 at 4h in Daxx KO can be explained by elevated H3K27Ac at this time point (Supplementary Figure S10). Reduced H3K27Ac by AR KO and HIRA KO (Figure 4 and Supplementary Figure S10) would predict for decreased BRD4 accumulation in these cell lines. Indeed, we observed that AR KO abolished BRD4 accumulation, confirming H3K27Ac function in BRD4 binding to chromatin (compare Figures 7 and 9) and illuminating a role for AR in BRD4 recruitment at enhancers that is reciprocal to reported BRD4-dependent AR recruitment (75). In HIRA KO cells, BRD4 dynamics are similar to AR (compare Figures 9 and 5, Supplementary Figures S7 and S12): it is elevated at 4h of stimulation (potentially by co-recruitment with its interaction partner AR (75)), but is reduced to the androgen deprived levels at 12 h (Figure 9 and Supplementary Figure S12 for time points comparison). Together, these data suggest new H3.3/H3K27Ac function in dynamics of BRD4 and AR at enhancers and imply a two-step model of recruitment and retention of AR transcription complex at enhancers (see Discussion for details).

**Figure 10.**
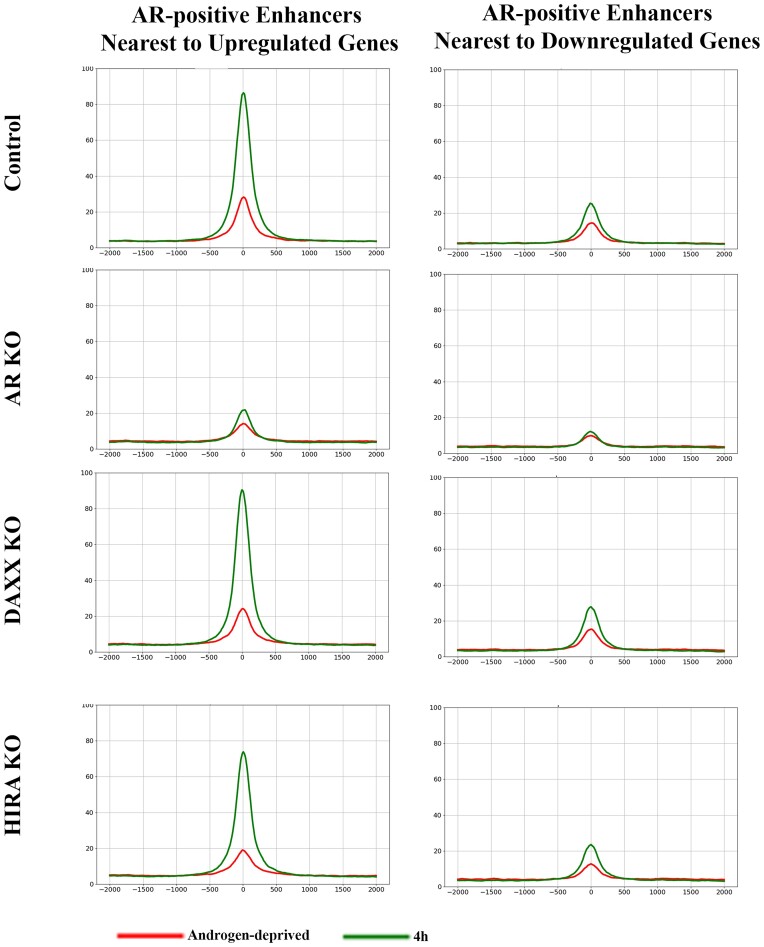
DNA accessibility analyzed of AR-positive enhancers by ATAC-seq. Metaplot of DNA accessibility at AR peaks (position ‘0’: AR at 4 h in R1-AD1 parental cells) at enhancers associated with up- (left) and down-regulated (right) genes in R1-AD1 parental (Control), Daxx KO and HIRA KO in androgen-deprived (72 h, red) and R1881 stimulated for 4 h (green). Stimulation for 4 h increased DNA accessibility at AR binding sites within enhancers associated with up- (to the higher extend) and downregulated genes, in all but AR KO cells; HIRA KO reduces DNA accessibility. See Supplementary Figure S13 for time points comparison.

**HIRA KO reduces DNA accessibility at AREs**. Changes in AR binding in HIRA-KO cells may suggest alteration of DNA accessibility that was tested by ATAC-seq. Androgen stimulation for 4h increased DNA accessibility at AR binding sites within enhancers associated with up- (to the higher extend) and down-regulated genes in all but AR KO cells (Figure 10 and Supplementary Figure S13 for time points comparison), perhaps due to the binding of pioneer transcription factors (76) that results in nucleosome shift/eviction as seen by the loss of H3.3 peaks at this time point (Figure 6, Supplementary Figure S9). H3.3 is associated with elevated DNA accessibility at the regulatory elements (19) that affects the binding of transcription factors as was recently shown at promoters in ES cells (56). We observed that HIRA KO cells, in line with reduction of H3.3, have decreased DNA accessibility under androgen-deprived conditions and at 4 h of induction at AR-binding sites within enhancers (Supplementary Figure S13). To evaluate significance, we performed a paired-sample t-test, comparing ATAC-seq between parental and HIRA KO cells at AR-positive AREs associated with up- or downregulated genes, at 0 and at 4 h of induction. Using a *P*-value threshold of 10e-4 (corresponding to a 5% significance level with Bonferroni correction for multiple testing), we observed that the difference between HIRA KO and Parental cells is significant in all cases. Therefore, elevated AR binding at 4h cannot be explained exclusively by pre-existing DNA accessibility due to reduced H3.3 nucleosomes loading in these cells.

**Figure 11.**
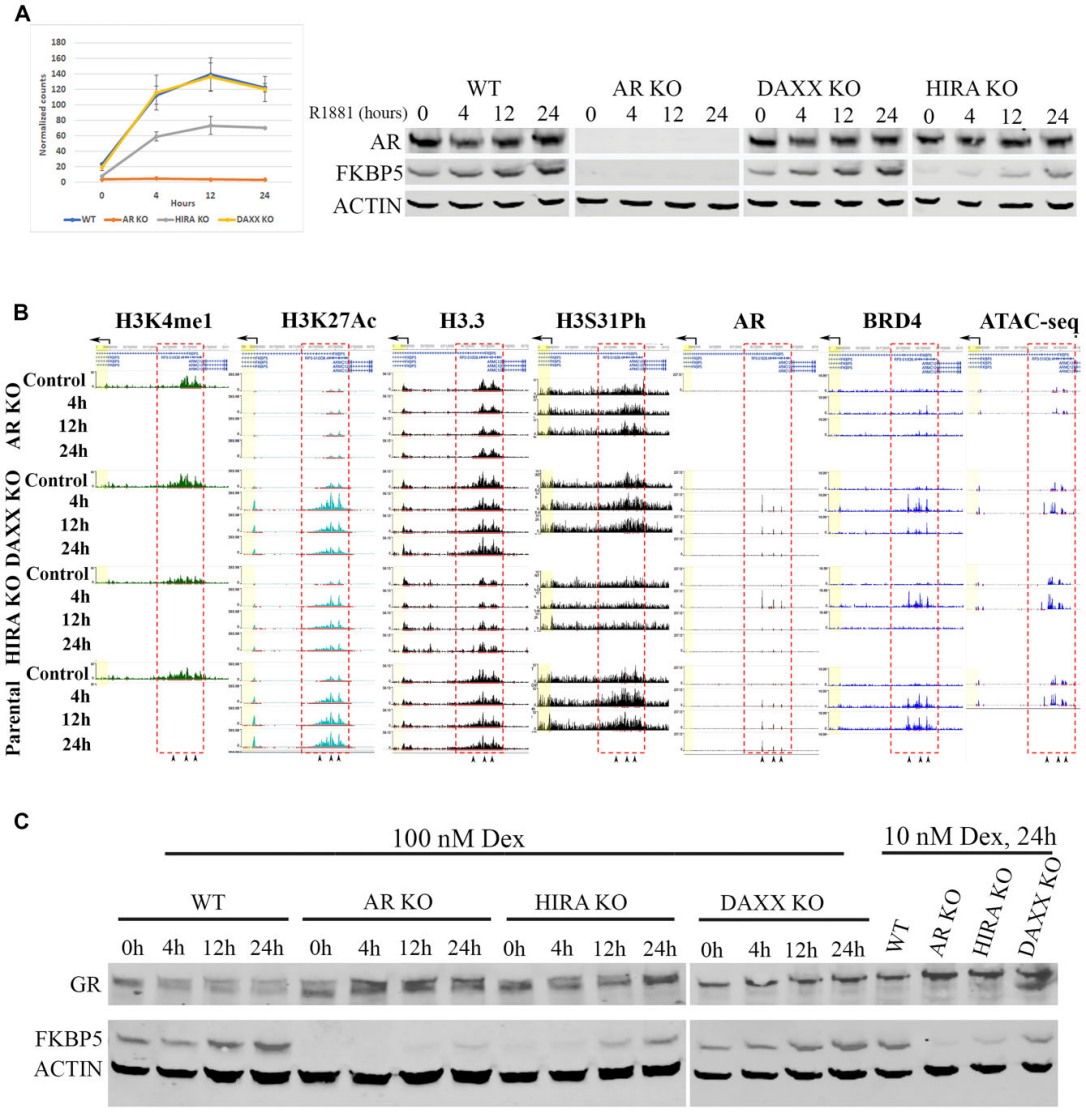
FKBP5 gene and SE are regulated by AR, GR and HIRA. (**A**) Expression of FKBP5. R1-AD1 cells (parental, AR KO, Daxx KO and HIRA KO) were androgen-deprived for 72 h (control), induced with 1 nM of R1881 for 4, 12, 24 h and subjected by FKBP5 RNA analysis at three bioreplicates (left) and western blot analysis with corresponding antibodies (right). AR KO and HIRA KO substantially reduced induction of FKBP5 at RNA and protein levels. (**B**) Epigenetic profiling of FKBP5 SE. R1-AD1 cells (parental, AR KO, Daxx KO, HIRA KO) were androgen-deprived for 72 h (Control) and induced with 1 nM of R1881 for 4, 12, 24 h. H3K4me1 (enhancer), H3K27Ac (active enhancer), H3.3, H3.3S31Ph, AR and BRD4 profiling were analyzed by ChIP-seq. DNA accessibility analyzed by ATAC-seq. (**C**) HIRA KO abolishes induction of FKBP5 gene by glucocorticoid receptor (GR). R1-AD1 cells (parental, AR KO, Daxx KO, HIRA KO) were hormone-deprived for 72 h (0), induced with indicated concentrations of dexamethasone (Dex) for indicated time, and analyzed by western blot with corresponding antibodies. AR KO elevates GR levels; AR and HIRA KO substantially reduced induction of FKBP5 protein levels by Dex. Actin: loading control.

**HIRA KO abolishes induction of FKBP5 gene and affects epigenetics profiling at FKBP5 superenhancer**. FKBP5 is a co-chaperone that, among other functions, regulates AR dimerization (77). Several AREs were identified upstream and downstream from FKBP5 TSS (78,79). FKBP5 gene transcription is induced by androgens (78), which was confirmed in our experimental system (Figure 11A). AR KO abolished androgen induction of FKBP5 at the RNA and protein levels. While Daxx KO had minimal effect on FKBP5 induction, HIRA KO reduced androgen induction of FKBP5. To investigate mechanism of HIRA-dependent regulation, we performed epigenetic analysis of AR-binding region upstream of the main FKBP5 TSS (80). H3K4me1 is elevated in a 20 kB region located 50 kB upstream of the main FKBP5 TSS (Figure 11B and Supplementary Figure S14B for the full-size images) at the position of a putative enhancer that was previously identified using reporter assay in non-small cell lung cancer cells (81). The ROSE (Rank Ordering of Superenhancers) protocol (45,68) analysis identified this region as a superenhancer (SE). HIRA KO reduced the size of H3K4me1-positive region, reproducing a tendency observed on other enhancers (Supplementary Figure S8). H3K27Ac ChIP-seq was used to evaluate status of this SE in androgen-deprived and androgen-induced conditions. In parental cells, H3K27Ac and H3K4me1 peaks overlap, confirming the active status of FKBP5 SE. H3K27Ac accumulated gradually during androgen induction (Figure 11B and Supplementary Figure S14B) thus reproducing H3K27Ac dynamics at AR-positive enhancers associated with upregulated genes (Figure 7 and Supplementary Figure S10). In AR KO cells, H3K27Ac is drastically reduced in androgen-deprived and stimulated conditions, indicating a poised SE. Daxx KO had minor effects on H3K27Ac at FKBP5 SE. Similar to the profile of gene bodies and enhancers, we observed a temporary (at 4 h) elevation of H3K27Ac in Daxx KO compared to parental cells. HIRA KO strongly reduced H3K27Ac in androgen deprived and stimulated conditions. We next profiled H3.3 and H3.3S31Ph at FKBP5 SE. In parental cells, H3.3 was enriched at FKBP5 SE (Figure 11B and Supplementary Figure S14B). Whereas Daxx KO had minor effects on H3.3 deposition, HIRA KO strongly reduced H3.3 at SE. H3.3S31Ph was accumulated at FKBP5 SE during induction in parental cells and, similarly to H3.3, was substantially reduced in HIRA KO cells (Figure 11B).

**HIRA KO affects AR and BRD4 binding to FKBP5 SE**. Three AR peaks that were previously mapped in VCaP and LNCaP cells (80) within FKBP5 SE identified above are conservative in R1-AD1 cells (Figure 11B). In parental cells, AR is chromatin-associated at all induction time points at all three peaks, while in HIRA KO it is induced at 4 h and is diminished at 12 and 24 h (Figure 11B and Supplementary Figure S14B), in concordance with dynamic of AR at enhancers (Figure 5 and Supplementary Figure S7). We also observed that BRD4 accumulates at all three AREs within FKBP5 SE in parental and in Daxx KO cells during induction (Figure 11B and Supplementary Figure S14B). AR KO abolished BRD4 accumulation, confirming AR and H3K27Ac function in BRD4 recruitment. In HIRA KO cells, BRD4 accumulates at 4h of stimulation and disappears at 12h, resembling dynamics of AR. In summary, HIRA/H3.3 dependent epigenetic changes observed at FKBP5 SE confirmed results of metaplot profile analyses. Same tendencies were documented at enhancers of additional genes deregulated by AR and HIRA KO (e.g. androgen up-regulated IL1R1, LONRF1 and down-regulated ID2; Supplementary Figure S14).

**HIRA KO affects DNA accessibility at FKBP5 SE**. Next we analyzed DNA accessibility at three AR binding sites within FKBP5 SE. PWMtools analysis (https://ccg.epfl.ch/pwmtools/) identified half-site ARE (82) at the peak 1 (ARE-1, Supplementary Figure S15) and canonical AREs (82) at peaks 2 and 3 (ARE-2 and -3, Supplementary Figure S15). Androgen stimulation for 4h induced an ATAC peak at the ARE-1 (half-site) in parental, Daxx KO and HIRA KO cells (Figure 11B, Supplementary Figure S14 and Supplementary Figure S15), indicating nucleosome shift/eviction at this half-site ARE. Pioneer transcription factor FOXA1 opens compact chromatin, increasing DNA accessibility (83). It interacts with AR (84), thereby regulating AR binding to DNA, mostly at half-site ARE, that requires FOXA1 for AR deposition to AREs resulting in nucleosomes displacement (85). FOXA1 binding consensus was observed to overlap with ARE-1 (*P*: 1.55e-06), suggesting FOXA1 function for chromatin opening and AR binding to this ARE. Therefore, increased DNA accessibility at this site can be explained by the androgen-induced binding of FOXA1 or another pioneer transcription factor that precluded AR binding. In androgen-deprived conditions, ARE-2 is more accessible in HIRA KO cells compared with parental cells (Supplementary Figure S15), suggesting an H3.3-dependent partial block of AR binding at some ARE, that is abrogated by HIRA KO.

**HIRA KO does not affect CTCF binding**. DNA binding protein CTCF regulates chromatin structure, including formation of topology associated domains (TADs) (86,87) that are important to maintain interchromatin interactions, including those of promoters/enhancers. Potential participation of CTCF in HIRA/H3.3-dependent chromatin structure was addressed by CTCF ChIP-seq. We identified two CTCF peaks at the 3′ end of FKBP5 SE and additional two peaks within FKBP5 gene body. None of these peaks were substantially affected in all KO cells, including HIRA KO (Supplementary Figure S14B), therefore excluding CTCF changes as a mechanism of deregulated FKBP5 expression.

**HIRA KO abolishes expression of genes co-regulated by AR and glucocorticoid receptor (GR)**. AR and GR have diverse physiological functions, yet their activation mechanisms are similar. Both of these nuclear receptors bind to almost identical DNA elements and are associated with a similar set of co-activators and co-repressors (88). In addition, both AR and GR induce expression of FKBP5 (80). Given this similarity, we asked whether H3.3/ HIRA dependent mechanism of AR-driven transcription regulation can be extrapolated to GR. To this end, we tested effect of GR induction by dexamethasone (dex) on expression of FKBP5. Using the same cell lines and induction time point as in Figure 11A, we observed that GR induced FKBP5 similarly to AR in parental and Daxx KO cells (compare Figures 11A and C). AR-KO elevated GR expression (by RNA-seq) and protein accumulation (Figure 11C), confirming a function of AR in suppression of GR expression (89,90). Surprisingly, AR KO, while elevated GR accumulation, almost completely abolished FKBP5 induction by dex, suggesting functional connection between AR and GR. HIRA KO reduced FKBP5 expression in GR-induced conditions (Figure 11C), indicating that H3.3/ HIRA dependent mechanism is common for AR and GR, and, potentially, for other members of nuclear receptors family. Next, we performed RNA-seq analysis in androgen-stimulated and glucocorticoid-stimulated conditions in parental and three KO cell lines, ligand-deprived and 4h of stimulation. Regulated genes were clustered into three groups: [i] regulated by androgen, [ii] regulated by both androgen and glucocorticoid, and [iii], regulated by glucocorticoid only. HIRA KO affected expression of genes that are regulated by AR and co-regulated by AR and GR, and had modest effect on genes that are regulated by GR only (Supplementary Table S3). Therefore, H3.3 pathway may have stronger effect on AR/GR co-regulated genes, potentially those that are regulated by enhancers that are associated with both NRs.

**In AR-V expressing CRPC cells, HIRA KO is lethal, and HIRA KD affects H3.3 levels at AR-positive enhancers associated with AR-regulated genes**. Gain-of-function mutations of AR (91) are implicated in the development of CRPC and in therapy resistance (92). A recently identified molecular mechanism of aberrant AR activation in CRPC is expression of AR variants (AR-Vs) with deleted LBD (AR-DLBD) (6). Clinical data and animal models confirm function of AR-DLBD in CRPC (6). Expression of AR-Vs, including AR-DLBD, was identified in metastatic CRPC (93); it facilitates treatment resistance to anti-androgens (6), and is associated with negative survival prognoses. Identification of co-regulators of AR-Vs may yield novel targets for CRPC treatment. To this end, we compared the effect of manipulation of the H3.3 pathway in isogenic R1-AD1 (AR-WT) and R1-D567 (AR-DLBD) cells (37). Characterizing these cells, we observed that AR-WT is retained in the cytoplasm and relocates to the nucleus upon androgen treatment (Supplementary Figure S16A). Distinctly, AR-DLBD is a nuclear protein under both androgen-deprived and androgen-treated conditions, confirming ligand-independent nuclear localization of AR-V. We modified R1-D567 cells for expression of FLAG-HA-tagged H3.3 (Supplementary Figure S16B) and produced AR KO and Daxx KO cells (Supplementary Figure S16C). In three independent experiments we failed to produce HIRA KO in R1-D567 cells. HIRA gRNA deleted both alleles with similar efficiency in R1-AD1 and R1-D567 cell lines (by sequencing and IF). Nonetheless, R1-D567 (AR-DLBD) cells did not survive HIRA KO during clone expansion. As R1-AD1 and R1-D567 cells are isogenic and differ only in AR (AR-WT vs AR-DLBD), our data suggest that CRPC cell lines are HIRA-dependent and those that express AR-DLBD (and, potentially, other AR-Vs) developed HIRA addiction, suggesting synthetic lethality. We next depleted (KD) HIRA by combination of shRNA and siRNA (Supplementary Figure S16C); while HIRA KO reduces levels of H3.3 in R1-AD1 cells (Supplementary Figure S1), downregulation of HIRA by KD does not have this effect (Supplementary Figure S16C), indicating that complete knockout of HIRA is required for H3.3 compensatory reduction. HIRA KD reduced cell survival (tested by colony formation assay) much stronger in R1-D567 compared with R1-AD1 cells (Supplementary Figure S17). We analyzed effect of HIRA depletion on cell growth in two additional cell lines: C4-2B (AR-WT), and 22Rv1 (AR-WT and AR-DLBD), using proliferation and colony formation assays. Depletion of HIRA reduced proliferation and colony formation in all tested PC cell lines (Supplementary Figure S17).

We next analyzed H3.3 association with AR-positive (using AR binding data in R1-D567 (37)) enhancers (using H3K4me1 ChIP-seq analysis in R1-AD1 cells) nearest to genes up- and down-regulated by AR KO compared to parental R1-D567 cells (identified by RNA-seq). In AR KO cells, H3.3 is reduced at enhancers associated with both up- and down-regulated genes (Supplementary Figure S16D) in a manner similar to H3.3 profiling in R1-AD1 (Figure 6, Supplementary Figure S9), confirming AR-mediated transcription in maintenance of this histone variant. As in R1-AD1 cells, we observed a major reduction of H3.3 in HIRA KD R1-D567 cells (Supplementary Figure S16D), validating H3.3/ HIRA pathway in PC cells expressing AR-V.

**HIRA complex deregulation in PC**. HIRA accumulation induces breast cancer metastasis by H3.3-dependent aggressive transcriptional reprogramming (31). Exploring the function of H3.3 in PC (TCGA and the Human Protein Atlas), we found that expression of two HIRA complex components, proteins HIRA and UBN1, is increased in tumor compared to normal prostate tissue (Supplementary Figure S18A). HIRA and UBN1 expression was also elevated in high/very high-risk PC groups as determined by Gleason scores (Supplementary Figure S18A). Accumulation of HIRA (Supplementary Figure S18B, C) and UBN1 (94) is associated with negative PC survival prognosis. HIRA expression was elevated in glandular epithelial cells, which are the main source of prostate adenocarcinoma (single cell analysis, the Human Protein Atlas https://www.proteinatlas.org;Supplementary Figure S18D). Combined with our results (Figure 1), these data suggest potential oncogenic function of HIRA-dependent H3.3 pathway in PC initiation and progression and point at H3.3/ HIRA pathway as a potential target for therapeutic intervention.

## DISCUSSION

Chromatin enriched in histone H3 variant H3.3 and histone H2A variant H2A.Z (28) is associated with elevated transcription activity (18). According to Jin and Felsenfeld (19), H3.3/H2A.Z-containing nucleosomes are less stable compared to nucleosomes with canonical histones, thereby providing DNA accessibility to the transcription machinery at the regulatory elements. During the last decade, multiple experimental evidences were accumulating that further explain H3.3 function in transcription regulation, including H3.3 association with transcriptionally active markers (H3K9Ac and H3K4me3 (9,14,20,21)), prevention of silencing markers (22,23), and recruitment of p300 activity for H3K27Ac at enhancers (33). These findings emphasize transcription-associated properties of H3.3, and recent results from the Banaszynski group demonstrated H3.3-dependent transcription factors recruitment to regulated elements (mostly TSS) of transcribed genes (56). In the current study, we demonstrated function of H3.3 chaperone HIRA in dynamic regulation of the main PC driver, nuclear receptor AR. Depletion of HIRA prevents PC cells growth *in vitro, in vivo* and deregulates androgen-induced expression. H3.3 is enriched at boundaries of highly expressed genes (14) and at enhancers (32). Investigating mechanisms of this HIRA-dependent deregulation, we found that HIRA KO reduces H3.3 incorporation on androgen regulated genes and on AR-positive enhancers, confirming function of HIRA in deposition of H3.3 at multiple genome elements in PC models with AR-WT and AR-DLBD. Androgen treatment induces accumulation of H3K27Ac at gene bodies, TSS and enhancers, yet to a lesser levels with those associated with repressed genes compared with induced ones. We also observed that H3K27Ac levels first increase at enhancers (4 h) and then at TSS (12 and 24 h for up- and only 24 h for downregulated genes), suggesting potential transition of histone acetyl transferase (HAT) activity from one genome element to another, potentially via enhancer/gene contact. Elevation of H3K27 acetylation at repressed genes at 24 h might be at least partly explained by de-repression of some of these genes at the late timepoint; similar, but relatively minor accumulation of H3K27Ac at AR-positive enhancers associated with repressed genes corroborates this justification. HIRA KO diminishes levels of H3K27Ac in androgen-deprived conditions and reduces accumulation of this modification during androgen induction on regulated genes and nearby enhancers. Recent findings uncovered a function of H3.3 in activation of p300 that results in acetylation of H3K27 at enhancers (33). This function requires phosphorylation of H3.3S31 (H3.3S31Ph), a unique H3.3 residue, by the checkpoint kinase CHK1 (33,34) and the NF-κB kinase IKKα (35). These findings are consistent with our observations that reduction of H3.3 and H3.3S31Ph correlates with diminished levels of H3K27Ac in HIRA-KO cells.

**Figure 12.**
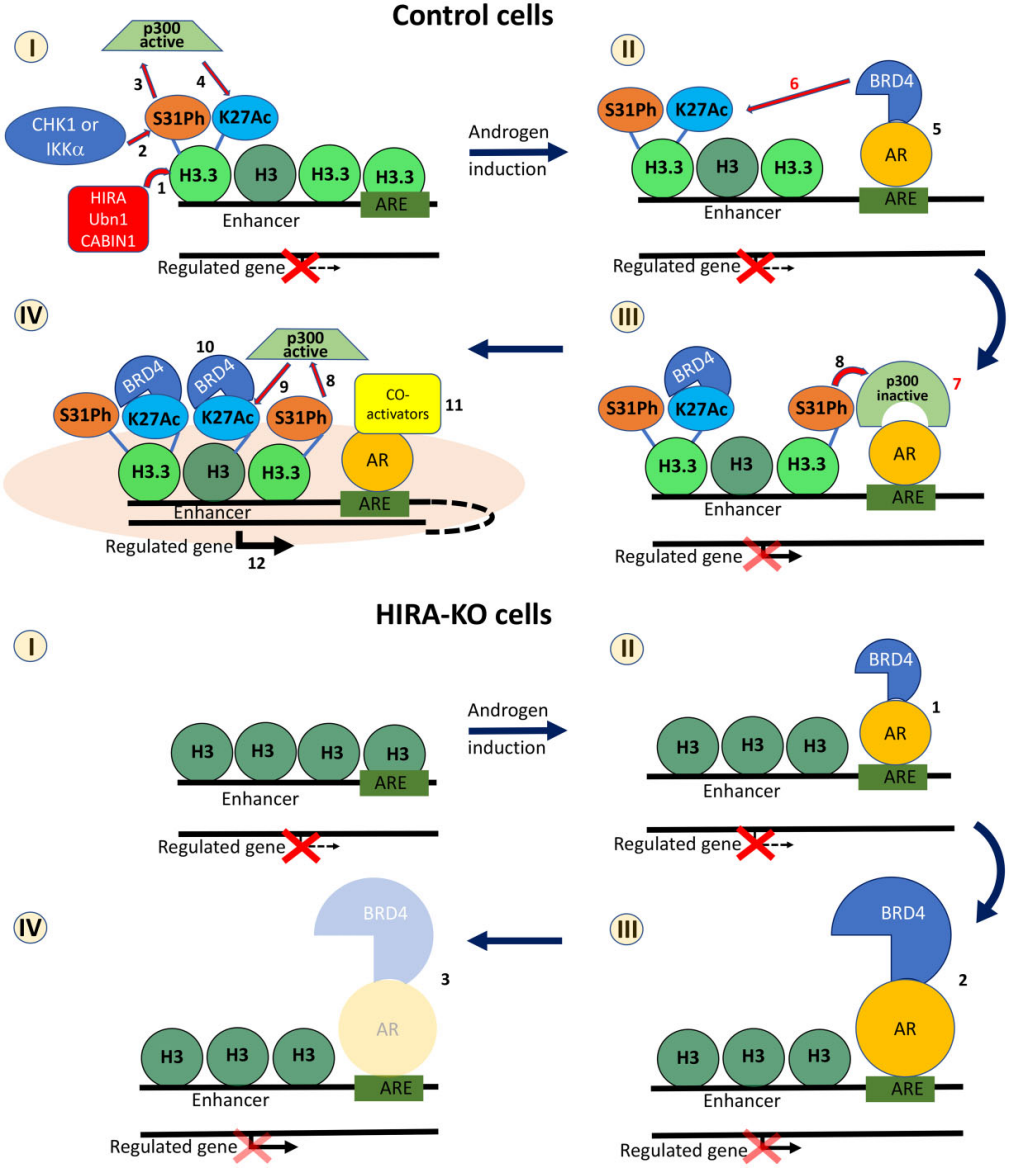
Model of HIRA/H3.3 function in AR transcription regulation. Control cells. (I) HIRA chaperone complex recruits H3.3 at enhancers (1). CHK1 and IKKa phosphorylates H3.3 at S31 (S31ph; 2), that activates p300 (3) for acetylation of H3K27 (4). (II) androgen treatment recruits AR to ARE that interacts with and co-recruits bromodomain protein BRD4 and displaces nucleosome at ARE (5); BRD4 binds to H3K27Ac at enhancer (6). (III) release of BRD4 from AR opens AR-NTD for recruitment of p300 (7) that is activated by increased levels S31ph (8, similar to 3). (IV) active p300 further acetylates H3K27 (9, similar to 4), resulted in additional binding of BRD4 to H3K27Ac at enhancers (10, similar to 6), recruitment of co-activators (11) and formation of completed transcription complex (pink cloud), enhancer-promoter contact and transcription activation (12). Steps 6 and 7 are speculative and thus highlighted in red. Model does not reflect several details and complementary scenarios, including but not limiting: effect of AR-dimer, co-recruitment of p300 and AR co-regulators, transfer of PolII from enhancer to promoter, BRD4-mediated transcription elongation. HIRA-KO cells. (I) No H3.3 recruitment, reduced H3K27Ac. (II) androgen induction recruits AR that interacts with and co-recruits BRD4 at AREs (1). (III) BRD4 cannot bind to chromatin due to reduced H3K27Ac, thus accumulating together with AR at AREs, and blocking recruitment of AR co-regulators (2). (IV) in the absence of co-regulators and reduced BRD4 binding to H3K27Ac at enhancers, AR and BRD4 have decreased retention at AREs (3); AR transcription complex does not form, and transcription regulation is weaker compared to parental cells.

Further analysis of HIRA-dependent deregulation of androgen-induced transcription revealed that HIRA KO changes accumulation dynamics of AR and BRD4 with enhancers associated with target genes. Specifically, AR and BRD4 levels were increased at 4 h after androgen stimulation followed by a reduction to pre-stimulation levels at later time points. Reduction of H3.3 at enhancers in HIRA KO cells did not increase accessibility of most AREs in androgen deprived conditions, therefore excluding the simple explanation of elevated AR binding at 4 h of induction in these cells. Instead, our data suggest a two-step assembly mechanism of functional AR complex at enhancer. At the first step (‘recruitment’), AR interacts with AREs (in some cases, with help of pioneer factors like FOXA1, as, potentially, at ARE-1 in FKBP5 SE) and BRD4 is co-recruited with AR via protein/protein interaction (75). At the next step, (‘retention’), BRD4 is transitioned from AR to H3K27Ac nucleosomes of enhancer. This results in stabilization of complex (including formation of multi-protein phase separation domain (95)) that may prevent dissociation of both BRD4 and AR from AREs, resulting in formation of active AR transactivation complex at enhancers and gene regulation (Figure 12). Confirming this model, it was shown that BRD4 is sufficient for AR-mediated transcription and BRD4 inhibition by JQ1 reduced both BRD4 and AR binding and androgen-dependent transcription (75). Our results documented interdependence between AR and BRD4 recruitment and dynamics of complex assembly, importantly, in the context of H3.3/ HIRA pathway: HIRA KO elevated the ‘recruitment’ and abrogated the ‘retention’, thus separating the first and the second (H3.3-dependent) steps of transactivation complex assembly suggested by our model (Figure 12).

The combination of RNA-seq and ChIP-seq results indicated that at 4h of androgen induction, when both AR and BRD4 are enriched at enhancers in HIRA KO cells, androgen-induced transcription is minimal in comparison with parental cells. Why the highly elevated AR and BRD4 are not able to regulate transcription? Our data suggest that recruitment of both AR and BRD4 in H3.3-reduced environment (e.g. in HIRA KO cells) is insufficient for the proper transcription regulation, potentially due to several (hypothetically interconnecting) possibilities: (i) due to the reduced H3K27Ac, BRD4 cannot bind to/retained at enhancers. BRD4 is required for AR activity (75) and BRD4 inhibition abrogates AR binding to AREs (75) that may reduce AR retention and transcription regulation; (ii) deregulation of BRD4 binding may induce changes in chromatin structure. Enhancers/SE are located distal to regulated genes (up to several megabases (96)) and they activate transcription by loop formation (97). BRD4 is indispensable for enhancer/SE activity, including enhancer/gene contact (68,98). Formation of chromatin loop between TSS and SE was previously proposed in activation of FKBP5 gene (81). Together with our emerging results, it suggests function of HIRA/H3.3/BRD4/AR in chromatin structure regulation, specifically in establishing enhancer/SE contact with regulated genes that may be abrogated in HIRA KO cells; (iii) HIRA KO can deregulate recruitment of AR co-activators or co-repressors. AR binds to BRD4 via NTD (aa 94–186 of AR), while BRD4 binds to AR via N-terminus that contains bromodomains BD1 and BD2 (75). In HIRA KO cells, levels of H3K27Ac are reduced at enhancers in androgen-deprived and androgen-induced conditions. It may diminish BRD4 binding to chromatin via H3K27Ac, thus hampering its dissociation from AR and blocking AR-NTD for recruitment of co-repressors or co-activators, including p300 that, in parental cells, may create a positive loop, further acetylating H3K27 and promoting BRD4 binding to enhancers; (iv) HIRA/H3.3 pathway regulates binding of PolII at TSSs (56) that correlates with reduced DNA accessibility in HIRA/ H3.3 KO ESC (56). We observed that HIRA KO cells reduced DNA accessibility (by ATAC-seq) at TSSs of AR-regulated genes, in androgen deprived and stimulated conditions (e.g. ATAC-seq at FKBP5 TSS, Supplementary Figure S14B), suggesting reduced PolII binding. Our results suggest additional H3.3-dependent mechanism(s) that promotes PolII transition from enhancer to TSS via promoter/enhancer contact within phase separated domains that are induced by BRD4 accumulation ((98); reviewed in (99)), or transition of PolII from initiation to elongation step, that is promoted by BRD4 ((100); reviewed in (101)). Altogether, our model opens several questions that require further investigation.

A recent publication demonstrated that H3.3 is sufficient for H3K27Ac enrichment and binding of BRD4 and transcription factors at promoters in ESCs (56). AR and GR have diverse physiological functions, yet their activation mechanisms are similar. Identification of co-regulators of GR and AR (including AR-Vs) may yield targets for effective and sustained management of CRPC (7). We observed HIRA-KO strongly reduced *FKBP5* induction by dex; HIRA KO deregulates GR-driven transcription of genes co-regulated by AR and GR, indicating that the H3.3/HIRA-dependent mechanism is common for AR and GR. In addition to basic NR biology, these results can be clinically relevant, as the GR pathway is functional in abiraterone- and enzalutamide-resistant CRPC (89).

AR KO reduced GR activation response despite accumulation of GR protein (Figure 11C), suggesting functional interplay between two pathways. One possible explanation is that inactivation of enhancers by AR KO (e.g. reduction of H3K27Ac, Figure 7, Figure 11) diminishes activity of another nuclear receptor, GR, that will bind to the similar DNA elements within these enhancers during activation by dex. Our data indicate that deregulation of enhancer activity by AR disfunction may require several cell cycles to deregulate enhancers, potentially by the negative feedback between reduced enhancer transcription that results in diminished transcription-associated loading of H3.3 (53–55), followed by H3.3-dependent H3K27Ac decrease. This model suggests functional connection between AR and GR and is important in the context of GR role in the enzalutamide resistant CRPC (89).

H3.3 is elevated at the enhancers at the late time points of androgen induction (Figure 6), implying transcription-dependent incorporation of H3.3. H3.3 is deposited through interphase by a transcription-dependent nucleosome replacement mechanism (11). Reciprocally, incorporation of H3.3 can create transcriptionally active chromatin by several mechanisms, including H3K27Ac increase (9,14,20,21). Circadian rhythms regulate production of glucocorticoid (102,103) and androgen (104) NR ligands, implying cycles in the NR-driven transcription. Together with our model (Figure 12), this suggests incorporation of H3.3 at the end of each NR activation cycle into transcribed genome elements, e.g. enhancers, for the maintenance of active chromatin that will prime the next round(s) of transcription by AR, GR, and, potentially, other nuclear receptors.

Advanced PC is a terminal disease and despite decades of intense laboratory- and clinical-based investigations, to date there is no cure or long-lasting effective treatment options. Initiation and progression of PC, including transition to CRPC, is determined by, among other factors, transcription reprogramming that is controlled by epigenetic dysregulation. Multiple lines of evidence point to the essential function of H3.3 deposition pathways in initiation and progression of bone tumors (105), pancreatic (106), brain (107) and breast (31) cancers. Exploring the function of H3.3 in PC, we found that expression of two members of H3.3 chaperone complex HIRA (HIRA and UBN1), is increased in tumor compared with normal prostate tissue, is elevated in high/very high-risk PC groups (concordant with increased Gleason score), and their accumulation is associated with negative prognosis. We observed that HIRA depletion obliterates PC cell growth *in vitro* and *in vivo* and deregulates expression of androgen-induced genes, including those essential for metastatic progression. In addition, we found that CRPC cells expressing gain-of-function AR variants (AR-Vs, implicated in antiandrogen resistance and development of metastatic CRPC (mCRPC)) are HIRA-dependent. Importantly, it was recently shown that HIRA accumulation induces breast cancer metastasis by an H3.3-dependent aggressive transcriptional reprogramming (31), thus emphasizing function of H3.3/ HIRA pathway in cancer versus normal conditions and providing additional scientific premise to our observations. Combined with our results (Figure 1), these data suggest potential oncogenic function of HIRA-dependent H3.3 pathway in PC initiation and progression and point at H3.3/ HIRA pathway as a potential target for therapeutic intervention. Levels of BRD4 (108) and p300 (109,110) are significantly elevated in high-risk PC patients. p300 and BRD4 inhibitors downregulate AR target gene expression and abrogate PC growth (75,111,112). Several inhibitors of CHK1, p300 and BRD4 are currently in clinical trials (phase 1 to 3), including p300 inhibitor CCS1477 (Inobrodib) (113), BRD4 inhibitor AZD5153 (114), and CHK1 inhibitors SRA737 (115). Hence, inhibition of BRD4, p300, CHK1 and IKKα is predicted to impair progression of PC by inactivating BRD4-dependent enhancers and the consequent decline of AR-driven transcription by reduced AR binding to AREs.

In summary, our data suggest that H3.3-enriched enhancer chromatin serves as a platform for H3K27Ac-mediated BRD4 recruitment, which interacts with and retains AR at enhancer AREs, resulting in AR-driven transcription reprogramming, thus identifying H3.3 function in nuclear receptors activity and placing HIRA complex at the epicenter of H3.3 pathways in PC initiation and/or progression.

## Supplementary Material

gkad700_Supplemental_Files

## Data Availability

The datasets generated and analyzed during the current study are available in the NCBI Gene Expression Omnibus repository in superseries GSE230744, which includes the following three series: GSE230529 (ATAC-seq), GSE230741 (RNA-seq), GSE230740 (ChIP-seq).
