# Supplementary material for: HIRA-mediated loading of histone variant H3.3 controls androgen-induced transcription by regulation of AR/BRD4 complex assembly at enhancers
Source: Nucleic Acids Res. 2023 Aug 28;51(19):10194–217. doi: 10.1093/nar/gkad700 (PMC10602887; doi:10.1093/nar/gkad700)
Supplement: gkad700_Supplemental_Files [file gkad700_supplemental_files.zip › Supplementary figures and legends combined.pdf]

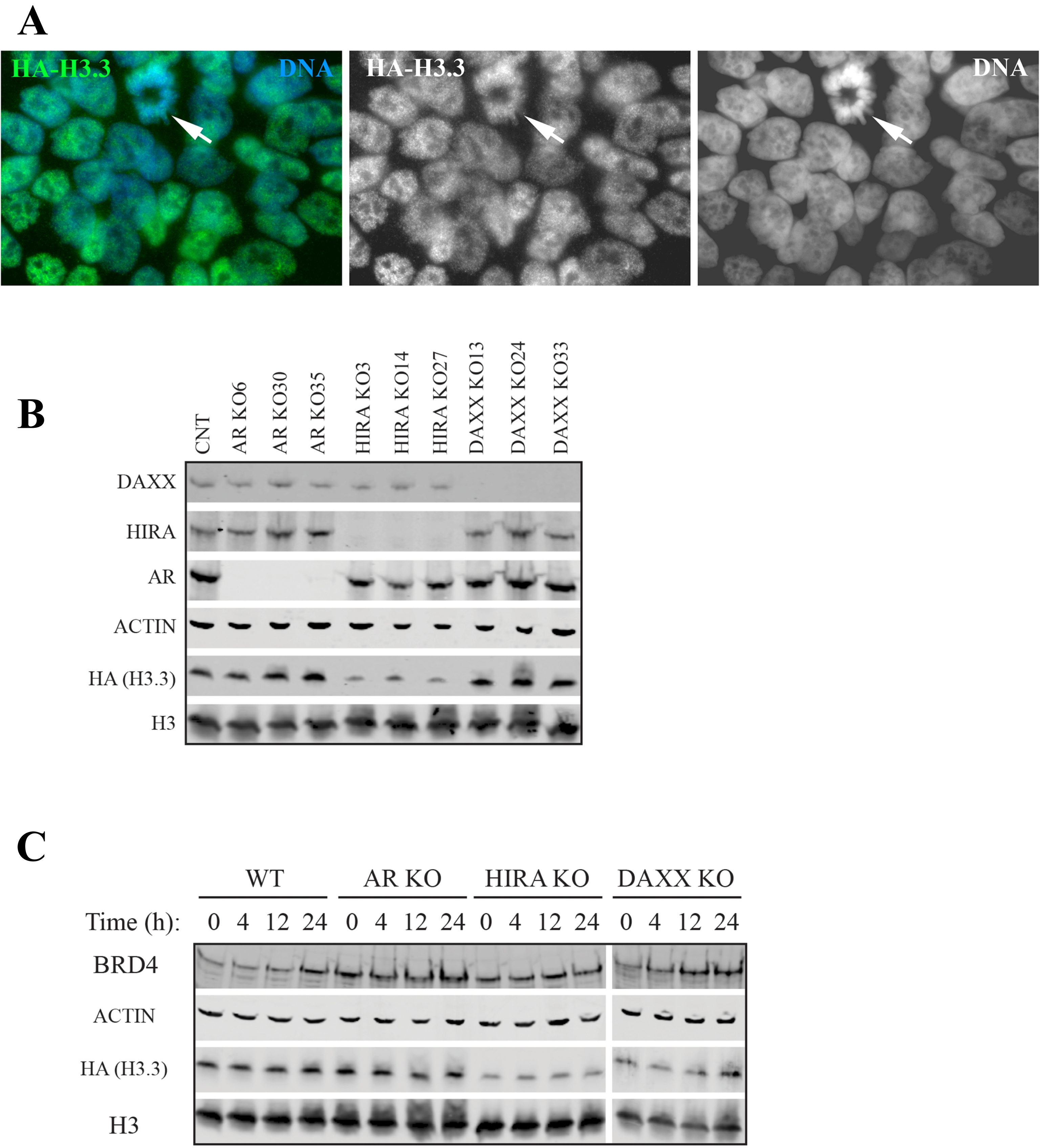

Fig. S1. Production and characterization of R1-AD1 (AR-WT) FLAG-HA-H3.3 knock-in and knockout cells.

Fig. S2. Expression analysis (RNA-seq) of R1-AD1 cells, parental (WT), AR KO, Daxx KO and HIRA KO.

A

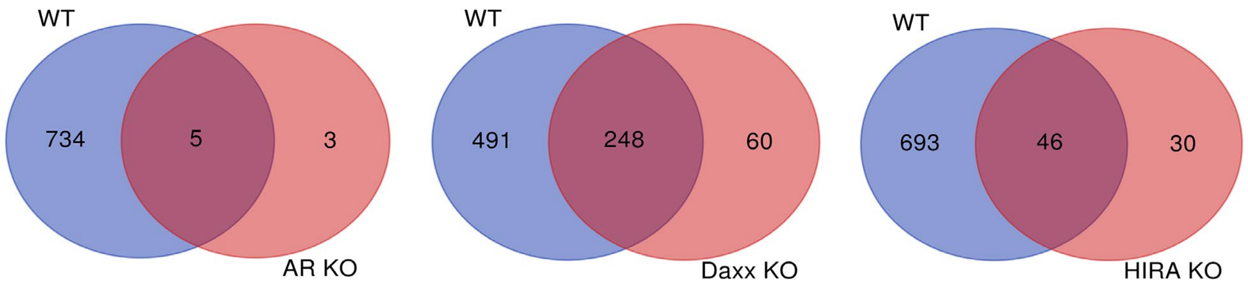

B

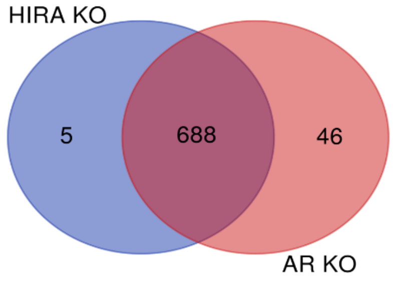

Cancer pathway

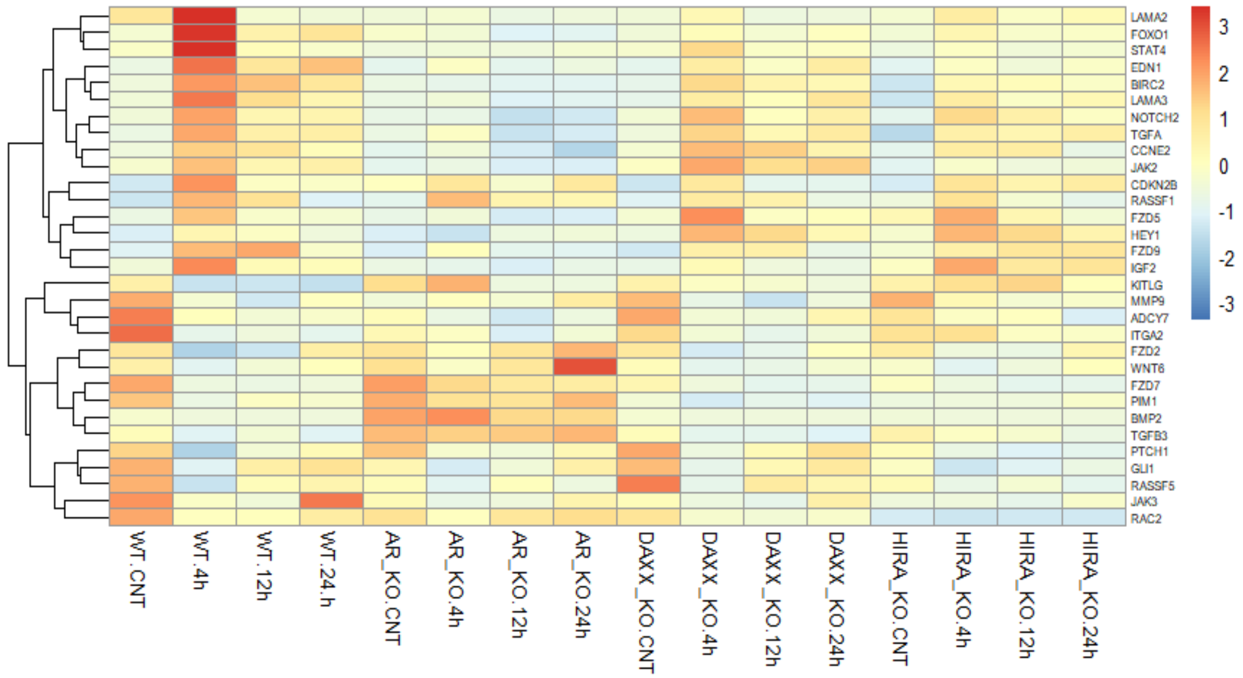

Hippo pathway

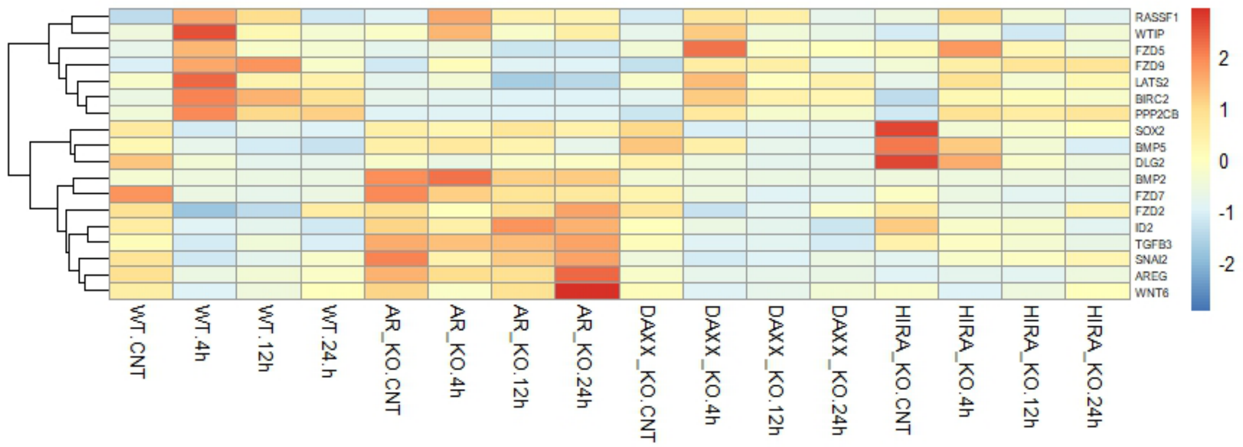

FoxO pathway

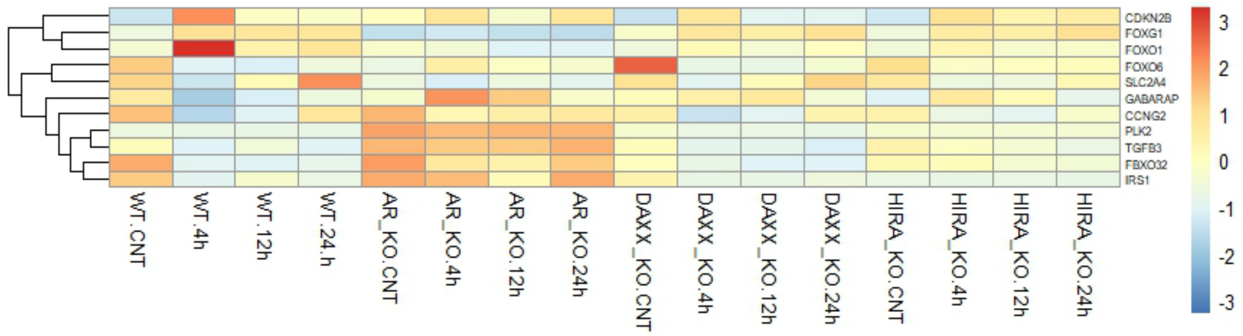

TGF-beta pathway

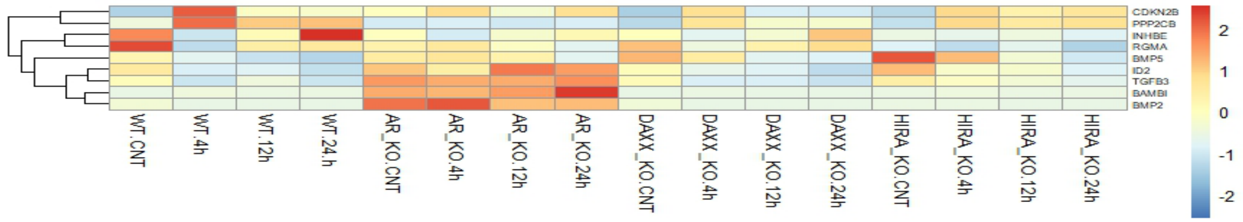

Wnt pathway

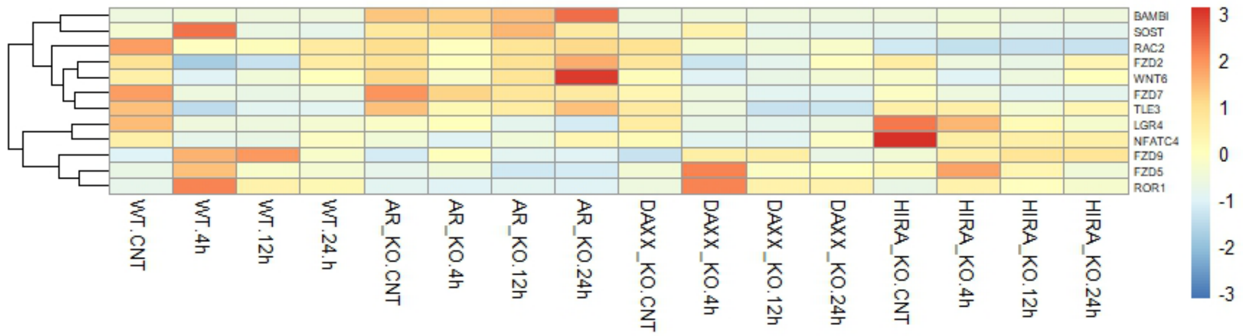

PI3-Akt pathway

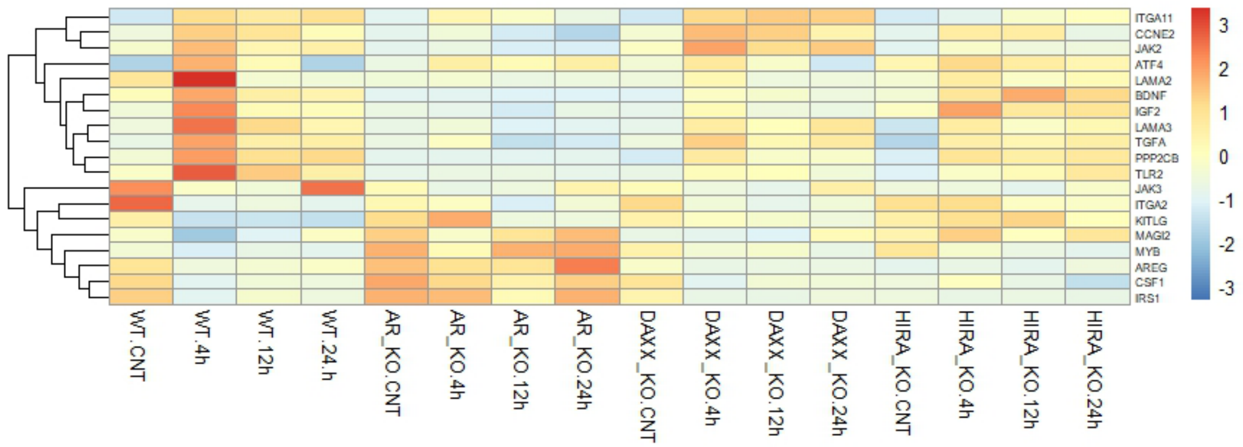

409 upregulated and 328 downregulated genes

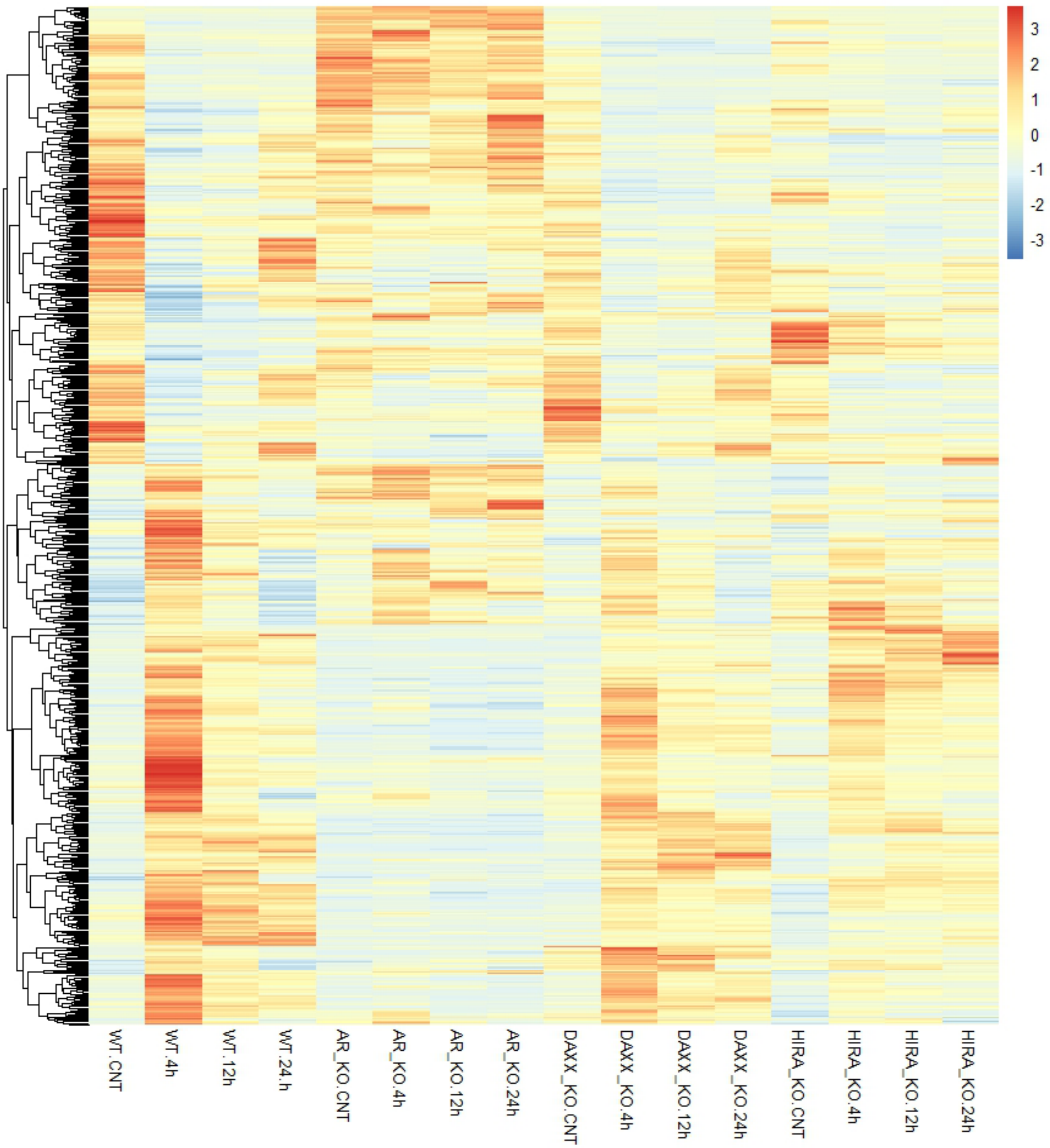

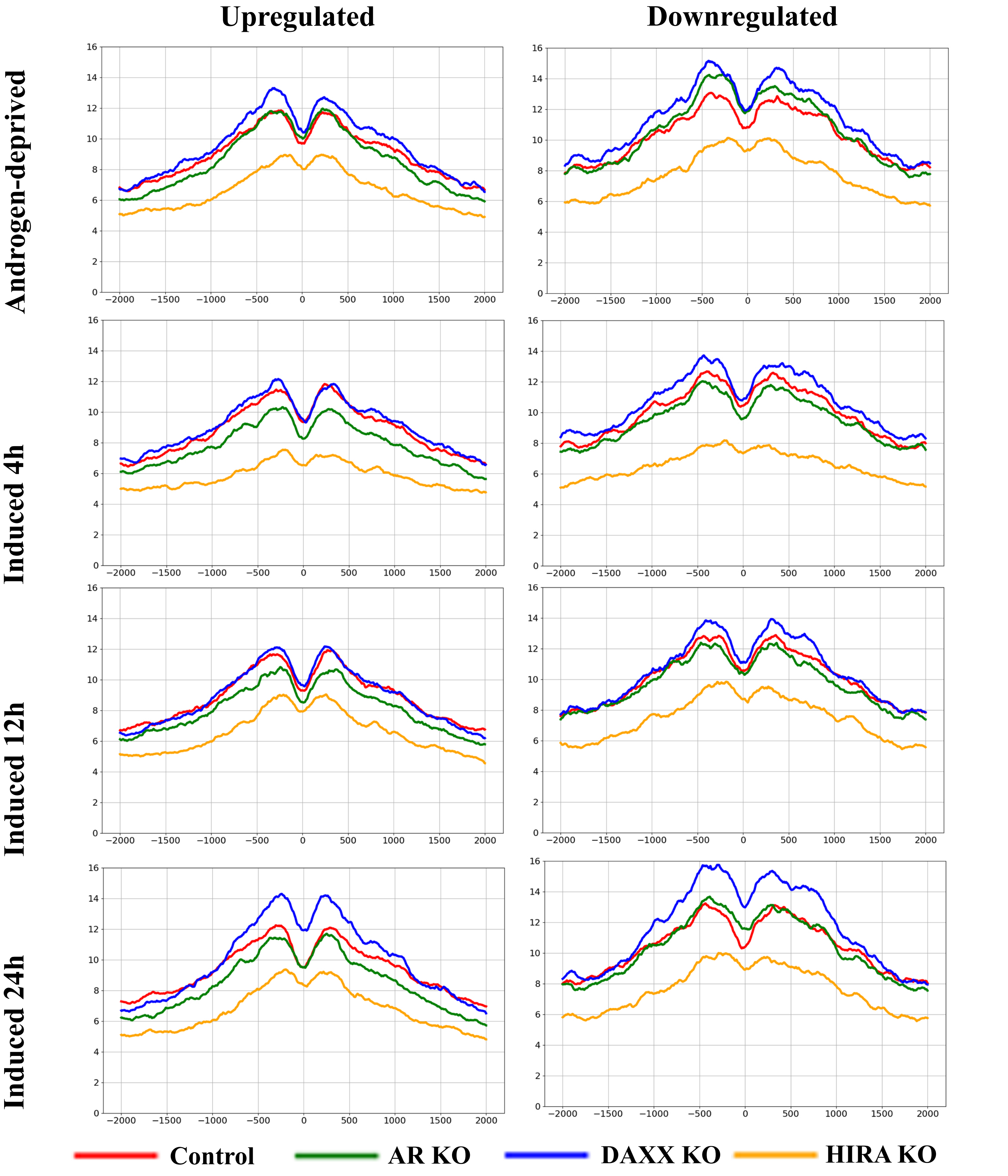

Fig. S3. H3.3 association with TSS of androgen-regulated genes; analysis within time points. H3.3 at transcription start site (TSS) of 409 upregulated genes and 328 downregulated at 4h, 12h, 24h postinduction. Y: arbitrary units.

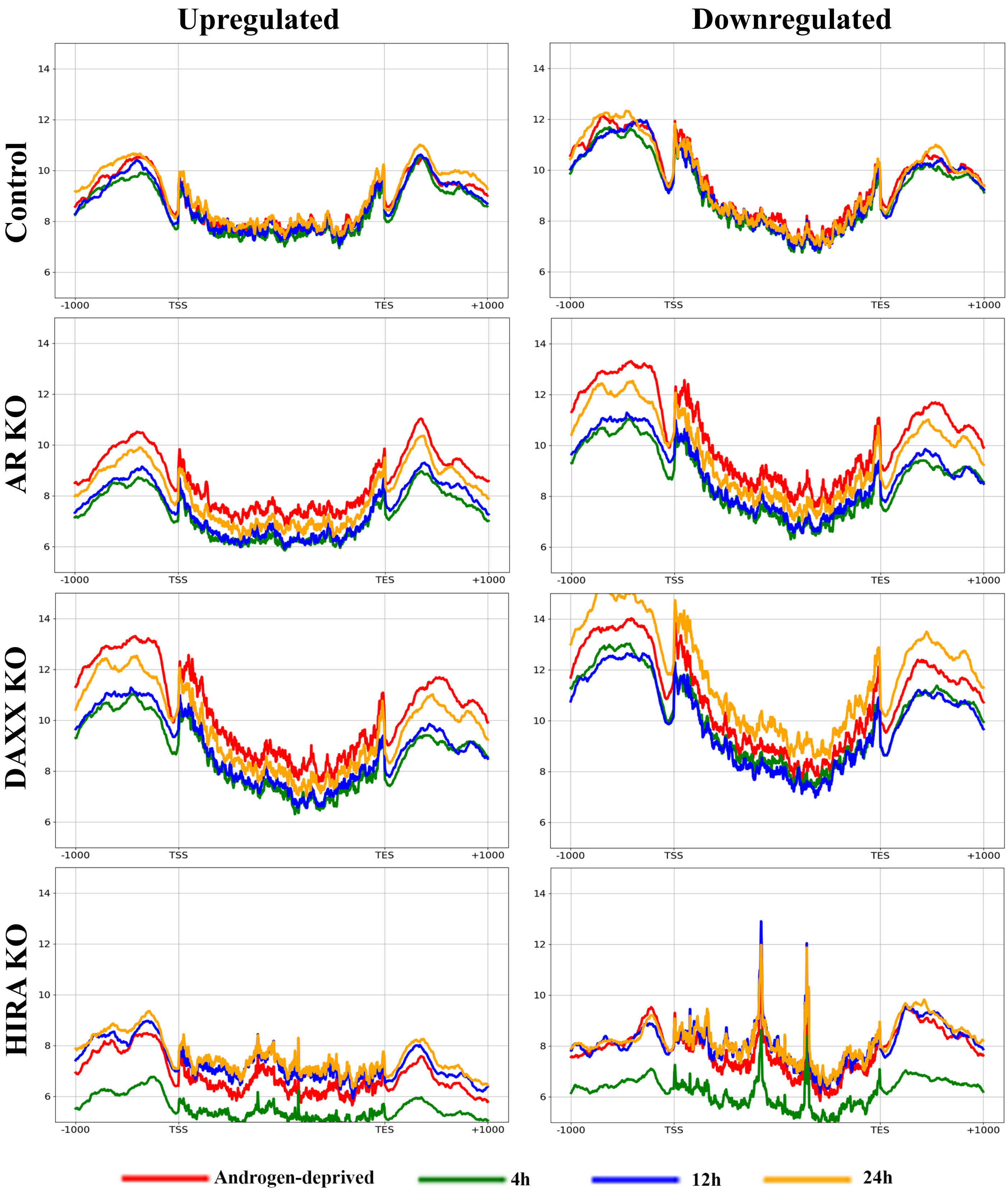

Fig. S4. H3.3 association with androgen-regulated genes; analysis within cell lines. H3.3 at 409 upregulated genes and 328 downregulated at 4h, 12h, 24h postinduction. TSS: transcription start site, TES: transcription end site. Y: arbitrary units.

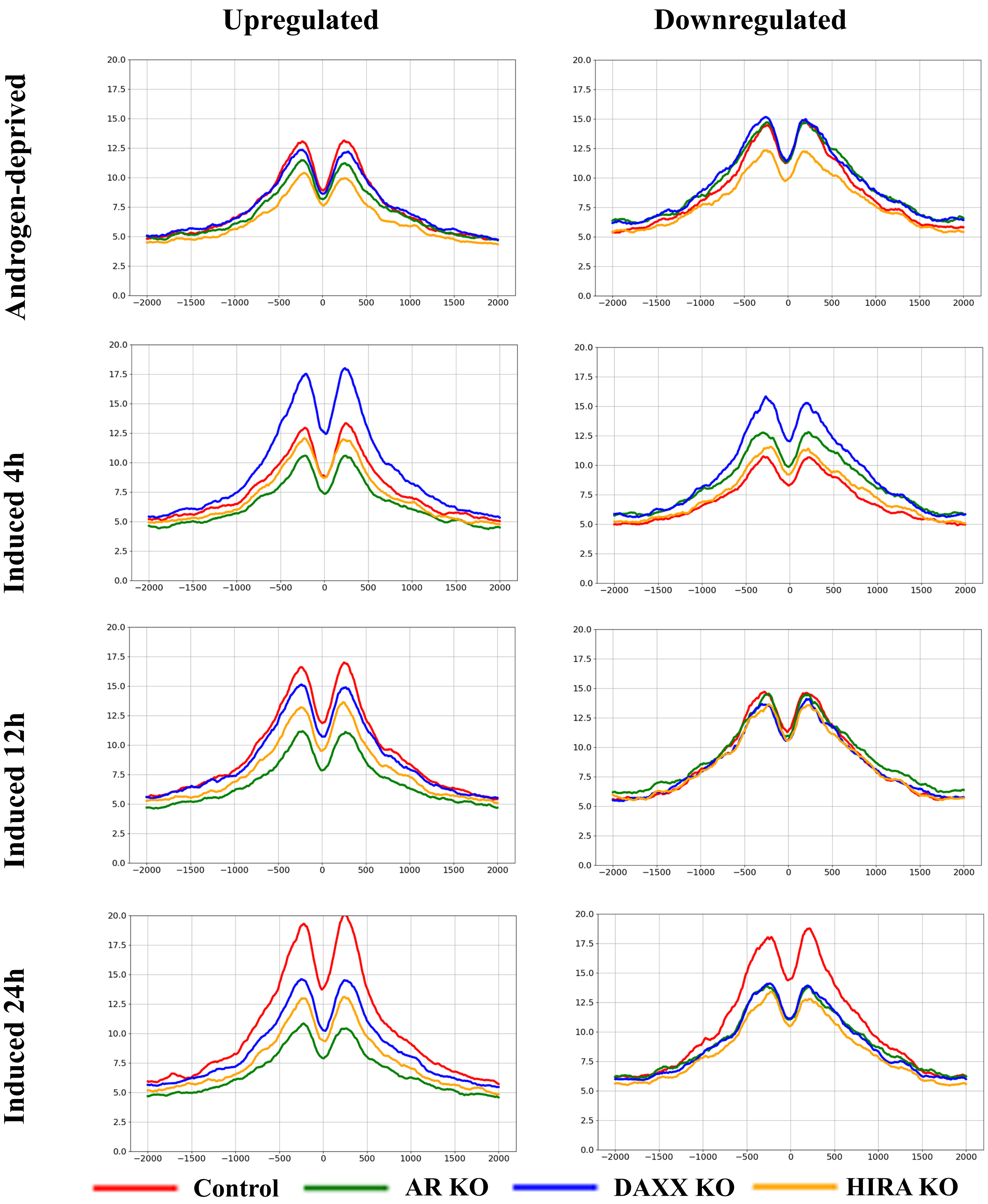

Fig. S5. H3K27Ac association with TSS of androgen-regulated genes; analysis within time points. H3K27Ac at transcription start site (TSS) of 409 upregulated genes and 328 downregulated at 4h, 12h, 24h post induction. Y: arbitrary units.

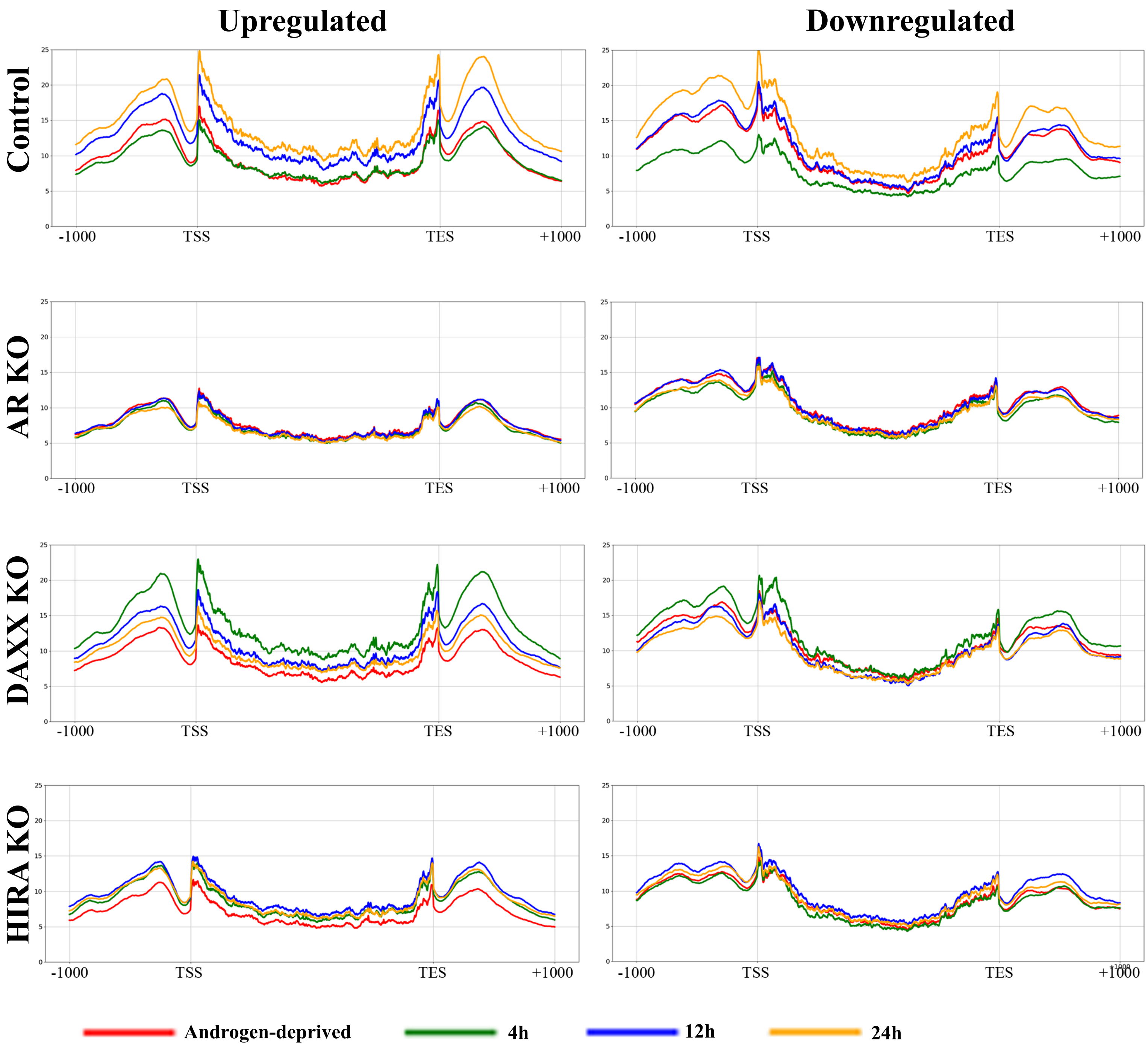

Fig. S6. H3K27Ac association with androgen-regulated genes; analysis within cell lines. H3K27Ac at 409 upregulated genes and 328 downregulated at 4h, 12h, 24h postinduction. TSS: transcription start site, TES: transcription end site. Y: arbitrary units.

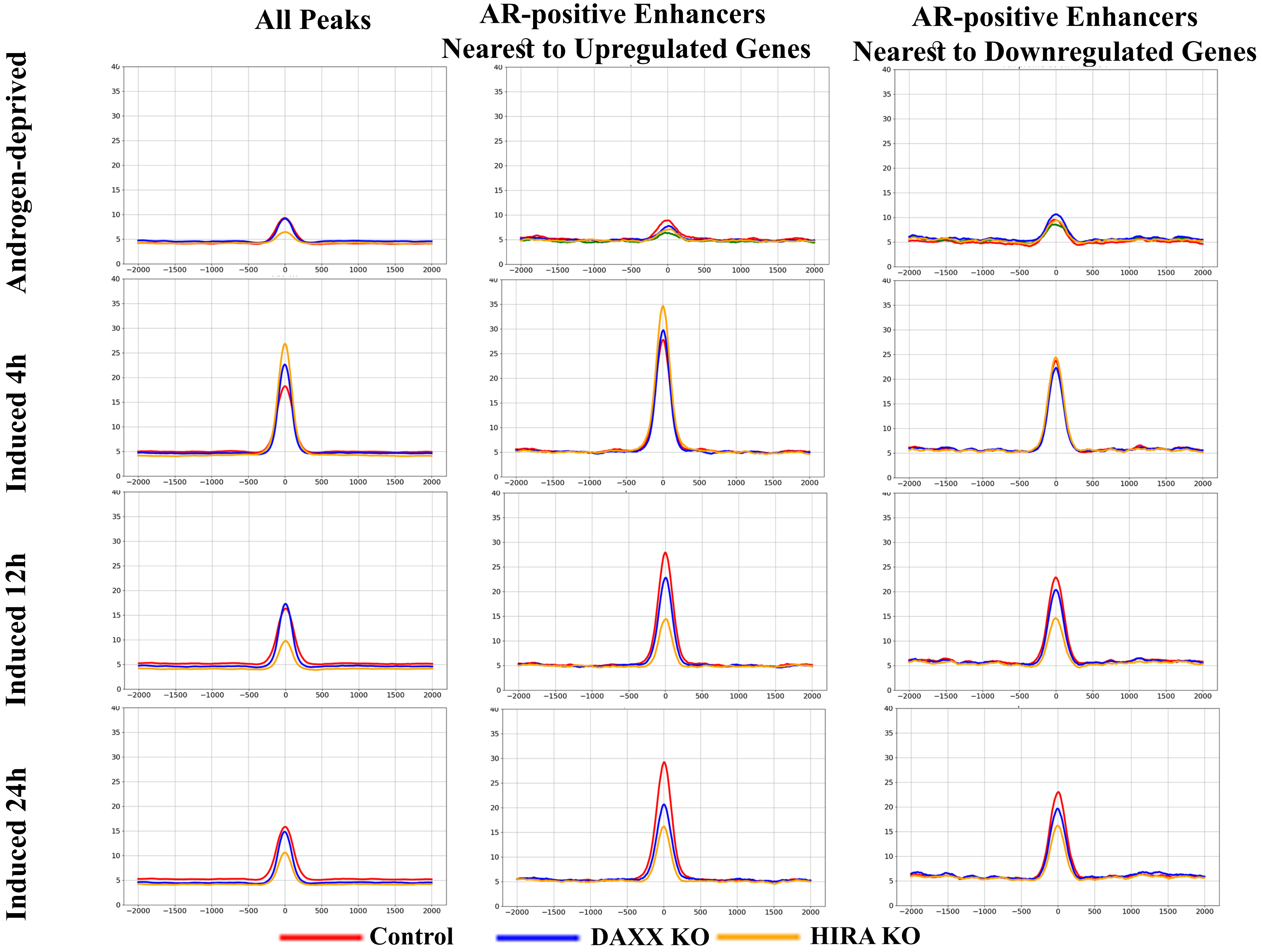

Fig. S7A. AR association with chromatin is regulated by H3.3 chaperone HIRA; analysis within time points.
AR at all peaks (left) and at enhancers nearest to 409 up- (middle) and 328 downregulated (right) genes in androgen-deprived, induced for 4h, 12h, 24h. X: distance from AR-binding site, bp. Y: arbitrary units

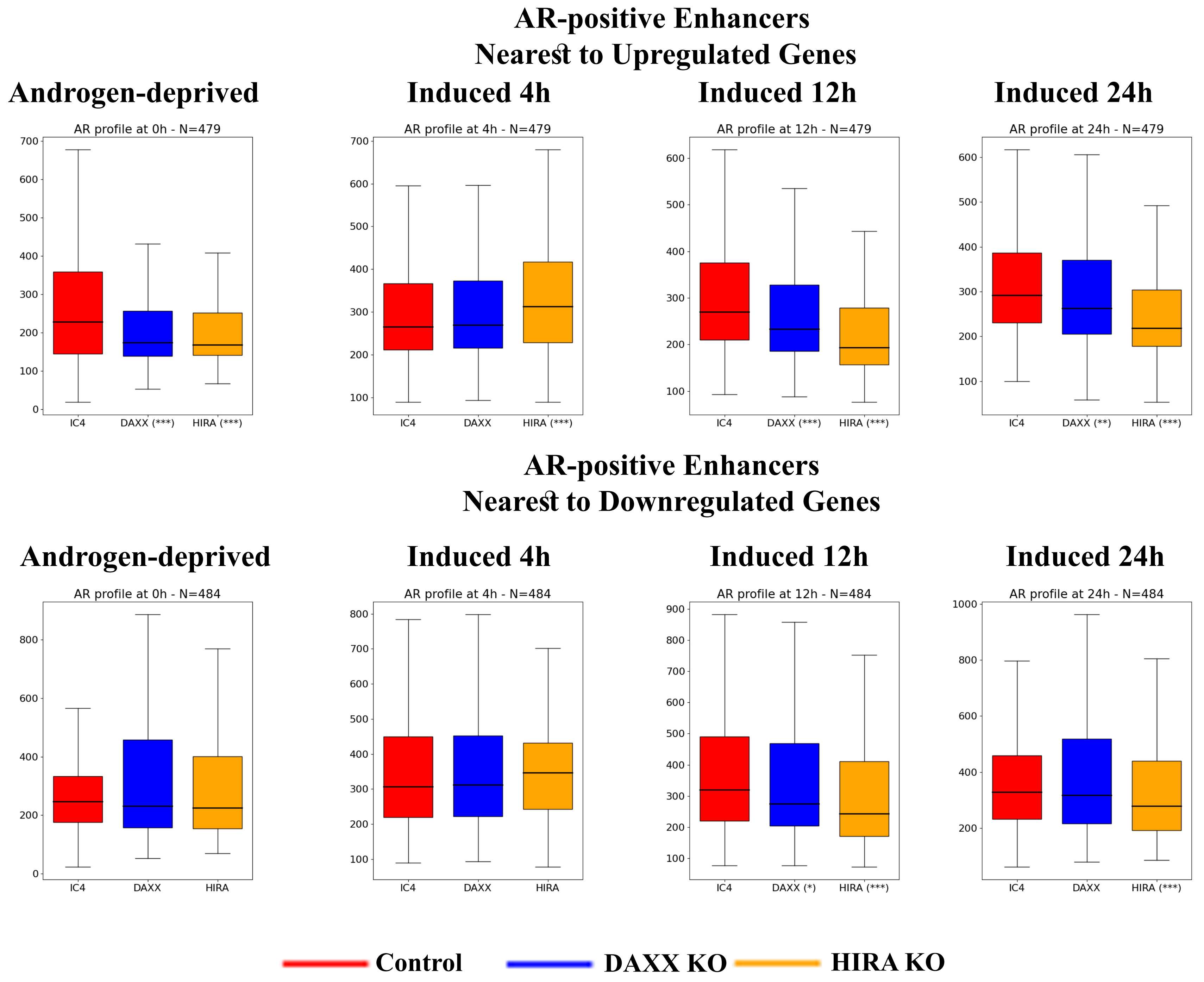

Fig. S7B. AR association with chromatin is regulated by H3.3 chaperone HIRA; analysis within time points
Boxplots of total AR signal around AR peaks in enhancers associated with 409 up- and 328 downregulated genes, androgen-deprived and induced for 4h, 12h, 24h. Y: arbitary units

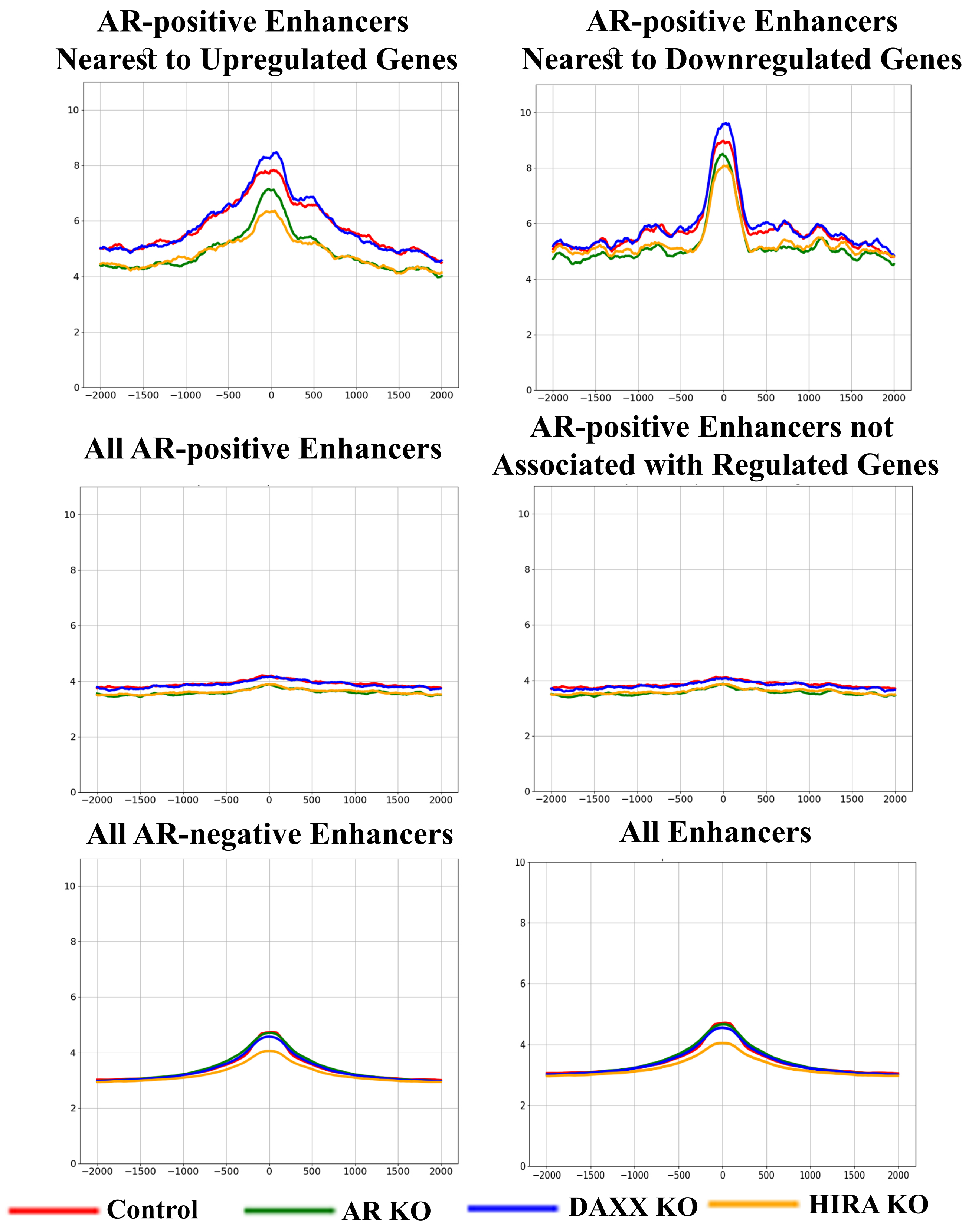

Fig. S8. H3K4me1 profiling at enhancers.
H3K4me1 at AR-positive enhancers nearest to 409 up- and 328 downregulated genes (top), all AR-positive enhancers (1980; middle left), AR-posotive enhancers that are not associated with regulated genes (1489, as some are sssociated with both up- and downregulated genes;middle right), all AR-negative enhancers (40644; bottom left) and all enhancers (42624; bottom right) in androgen-deprived conditions.

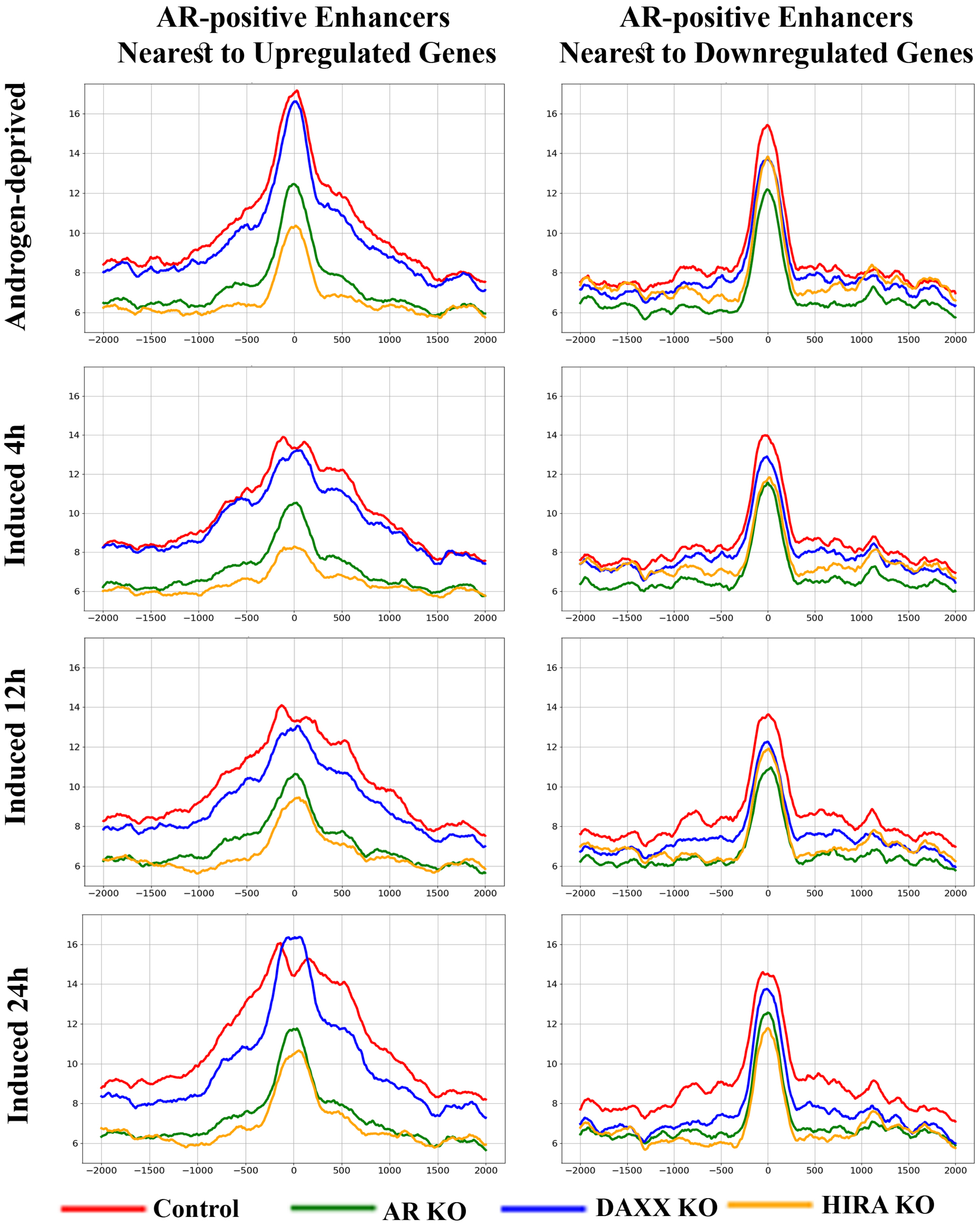

Fig. S9A. Dynamics of H3.3 at enhancers; analysis within time points.
H3.3 at AR-positive enhancers nearest to 409 up- and 328 downregulated genes in androgen-deprived and induced for 4h, 12h, 24h. X: distance from AR-binding site, bp. Y: arbitrary units

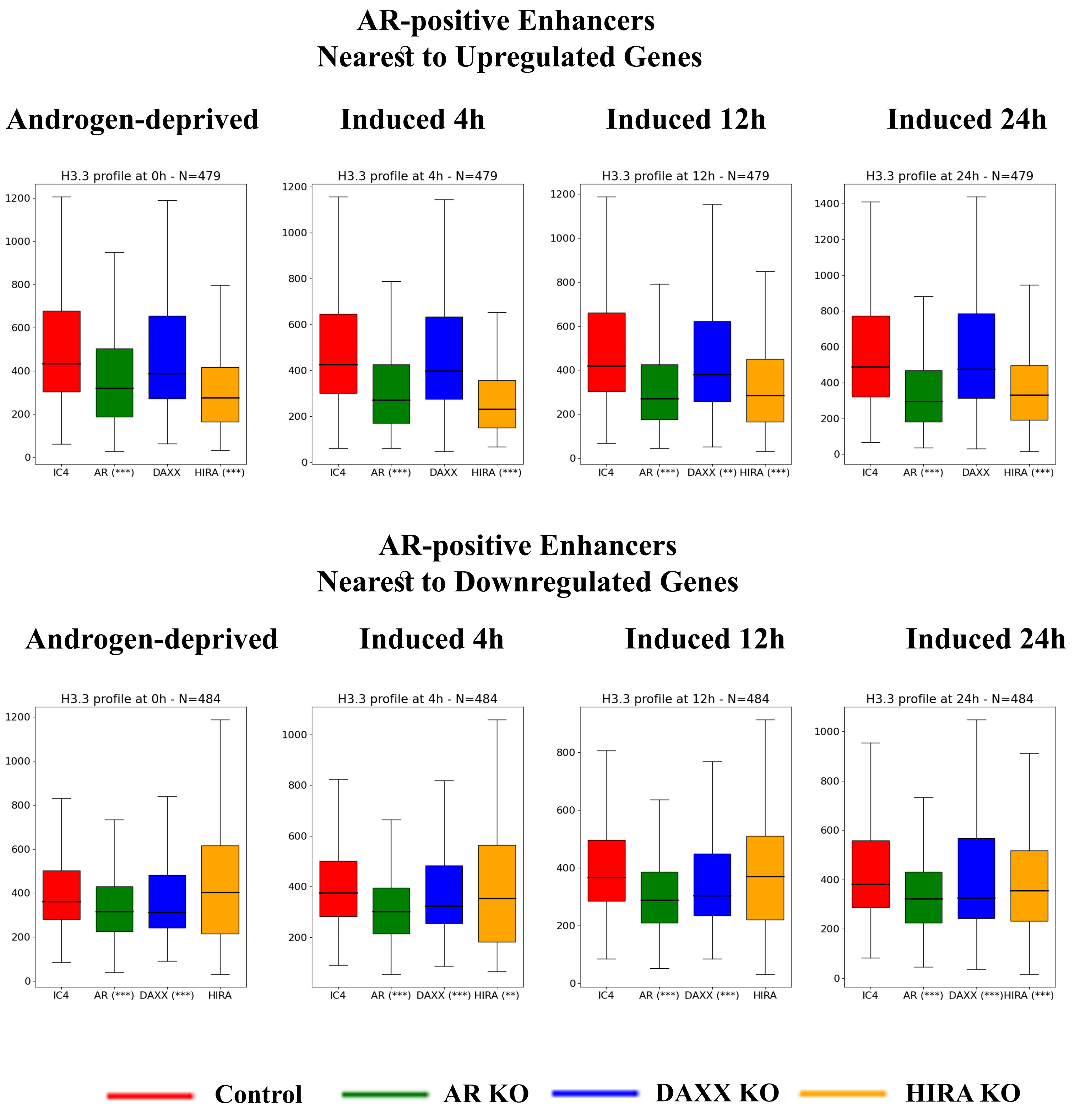

Fig. S9 B. Dynamics of H3.3 at enhancers; analysis within time points.
Boxplot of H3.3 at AR-positive enhancers nearest to 409 up- and 328 downregulated genes in androgen-deprived and induced for 4h, 12h, 24h. Y: arbitary units

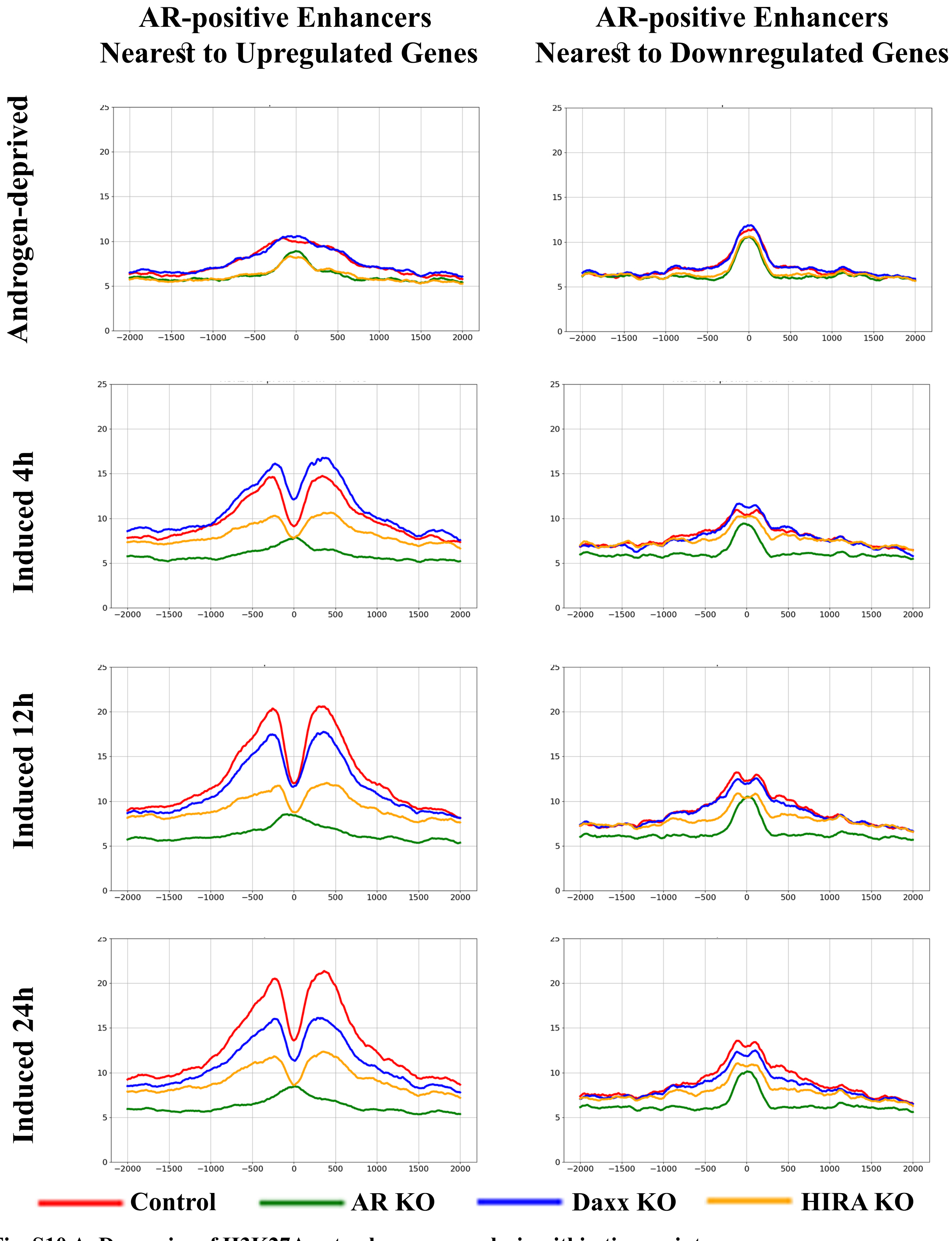

Fig. S10 A. Dynamics of H3K27Ac at enhancers; analysis within time points.
H3K27Ac at AR-positive enhancers nearest to 409 up- and 328 downregulated genes in androgen-deprived and induced for 4h, 12h, 24p. X: distance from AR-binding site, bp. Y: arbitrary units

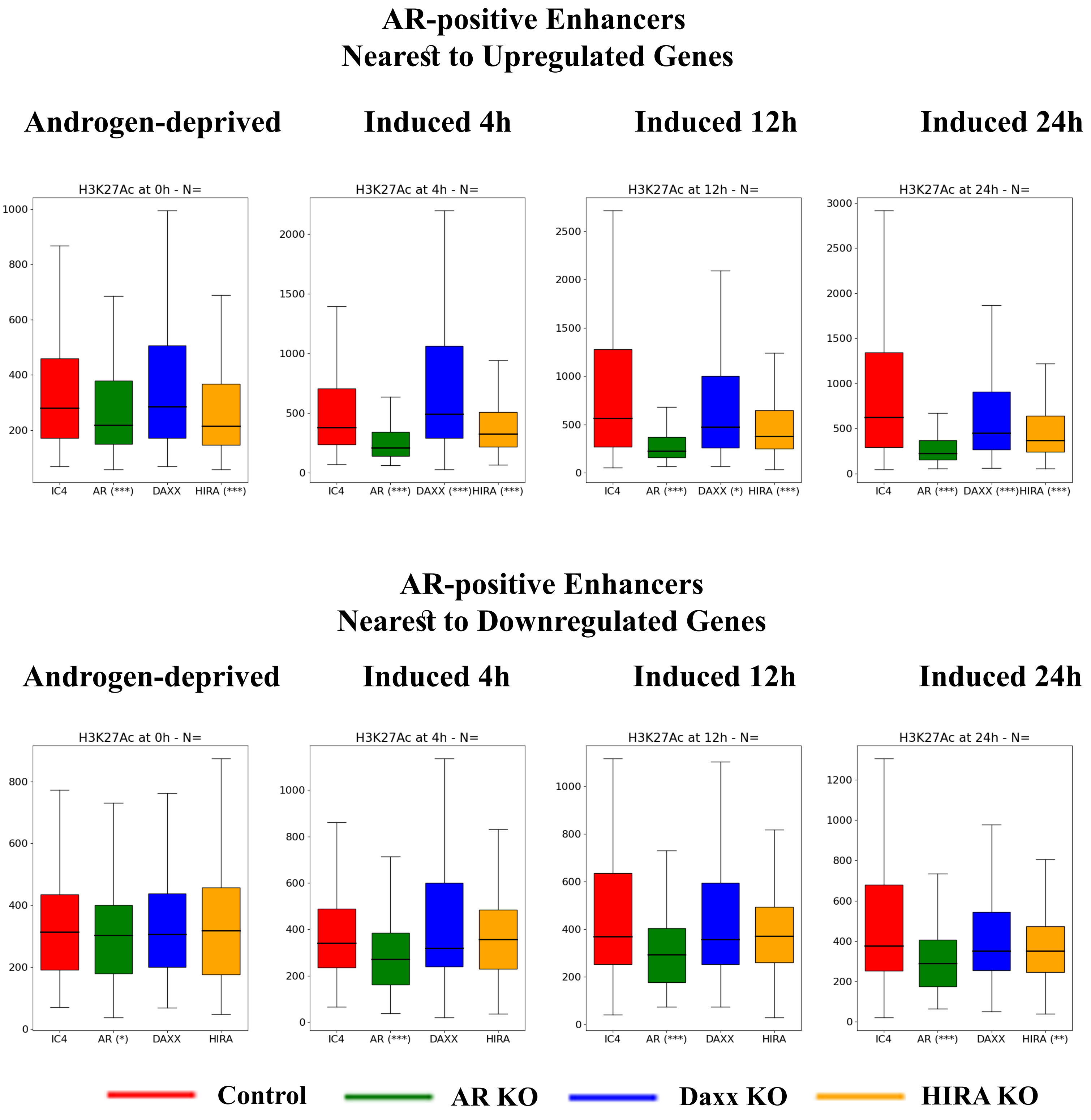

Fig. S10 B. Dynamics of H3K27Ac at enhancers; analysis within time points.
H3K27Ac at AR-positive enhancers nearest to 409 up- and 328 downregulated genes in androgen-deprived and induced for 4h, 12h, 24p. X: distance from AR-binding site, bp. Y: arbitrary units

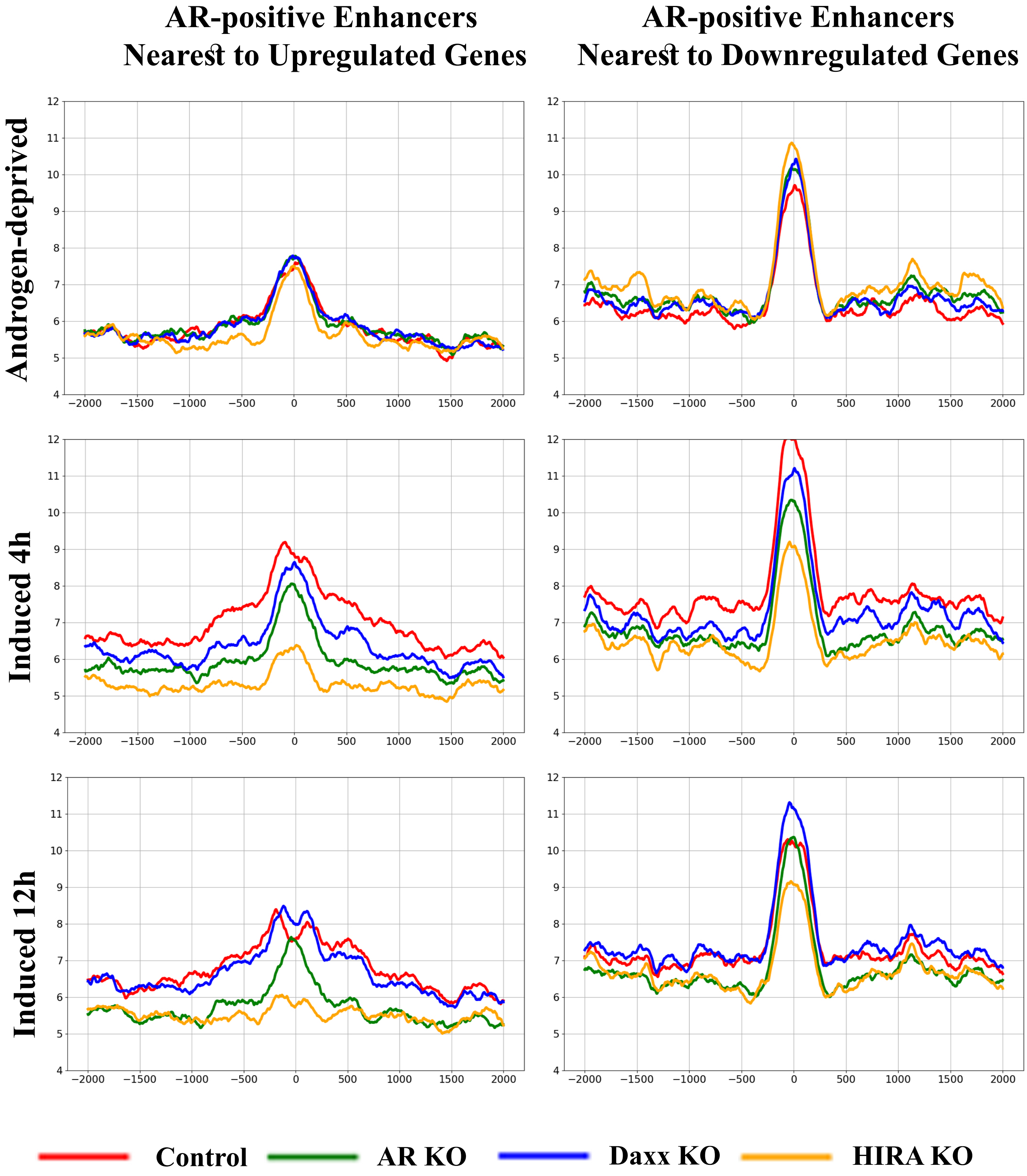

Fig. S11 A. Dynamics of H3.3 S31ph at enhancers/ SE; analysis within time points.
H3.3S31Ph at AR-positive enhancers nearest to 409 up- and 328 downregulated genes in androgen-deprived and induced for 4h, 12h. X: distance from AR-binding site, bp. Y: arbitrary units

AR-positive Enhancers
Nearest to Upregulated Genes

Androgen-deprived | **Induced 4h** | **Induced 12h**

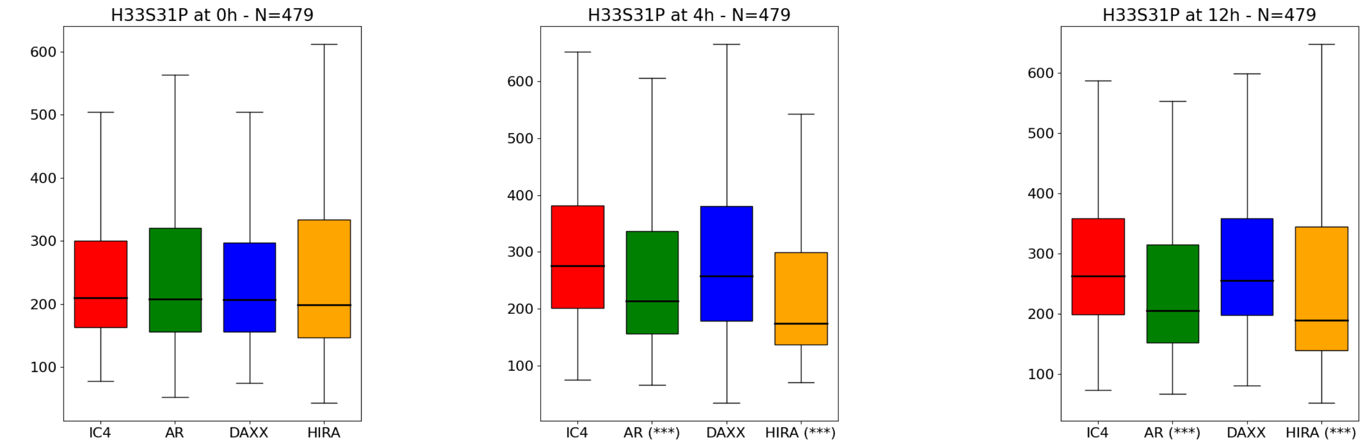

AR-positive Enhancers
Nearest to Downregulated Genes

Androgen-deprived | **Induced 4h** | **Induced 12h**

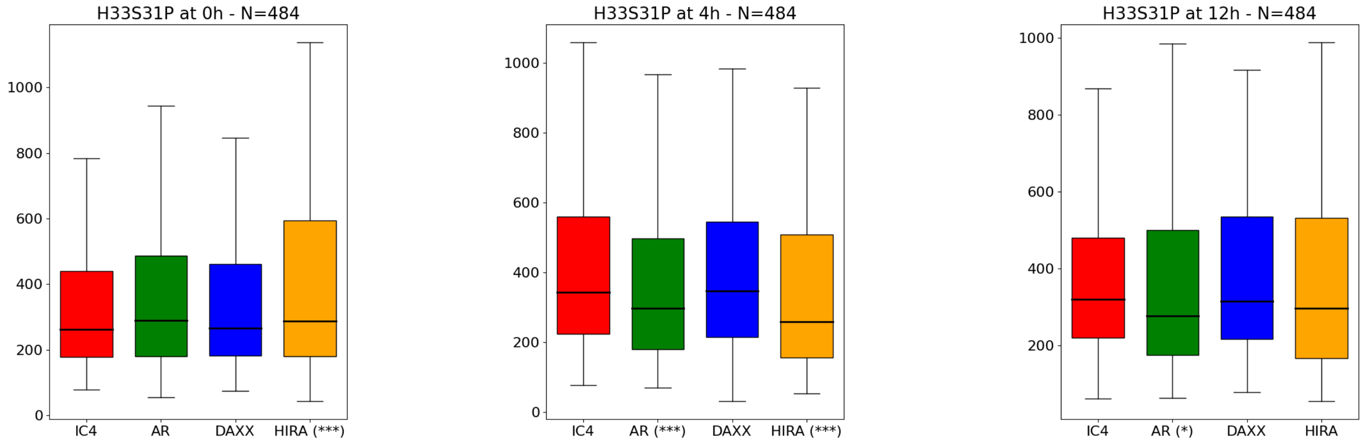

Control **AR KO** **Daxx KO** **HIRA KO**

Fig. S11 B. Dynamics of H3.3 S31ph at enhancers/ SE; analysis within time points. H3.3S31Ph at AR-positive enhancers nearest to 409 up- and 328 downregulated genes in androgen-deprived and induced for 4h, 12h. X: distance from AR-binding site, bp. Y: arbitrary units

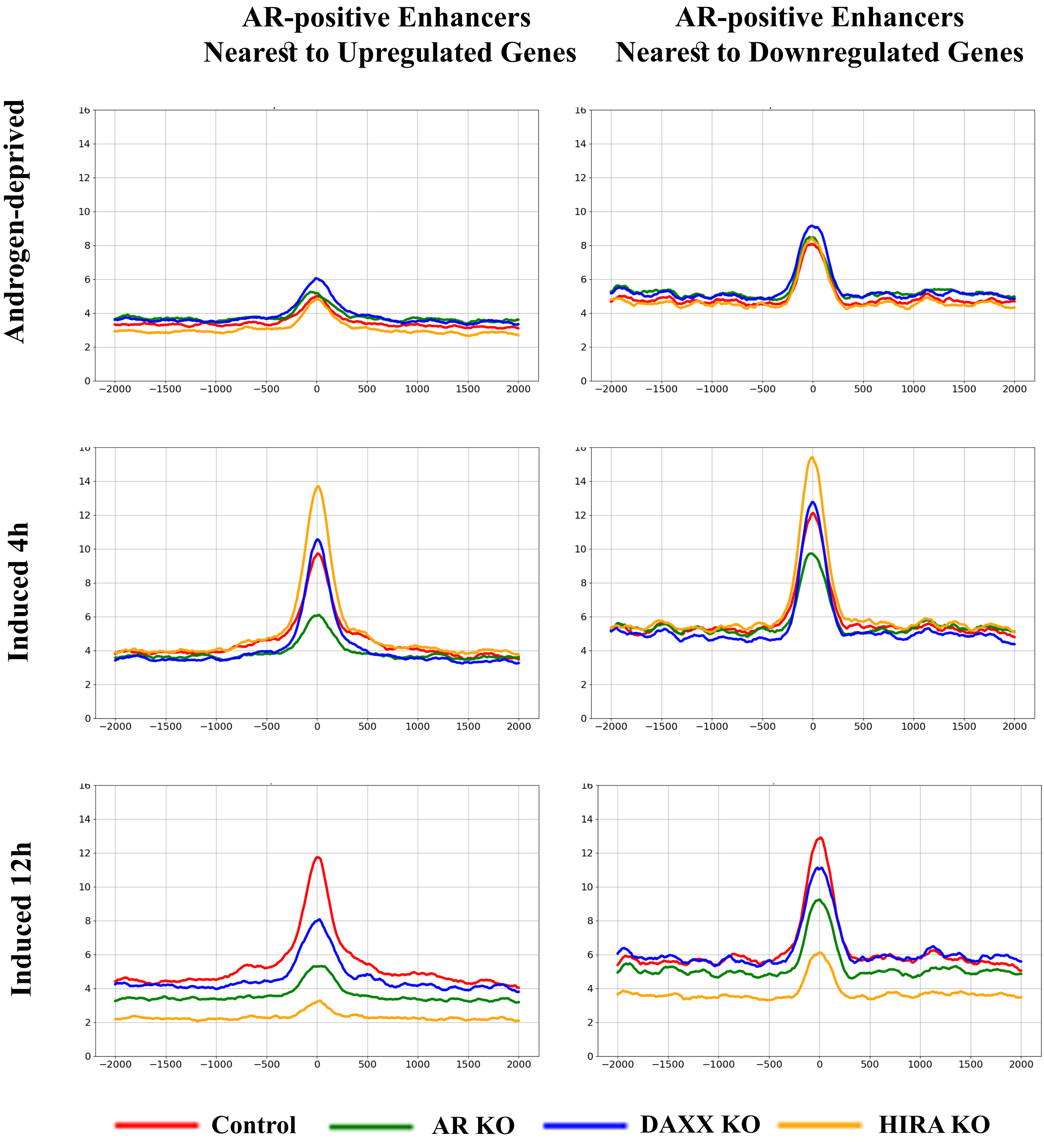

Fig. S12 A. Dynamics of BRD4 at enhancers; analysis within time points.
BRD4 at AR-positive enhancers nearest to 409 up- and 328 downregulated genes in androgen-deprived and induced for 4h, 12h, 24h. X: distance from AR-binding site, bp. Y: arbitrary units

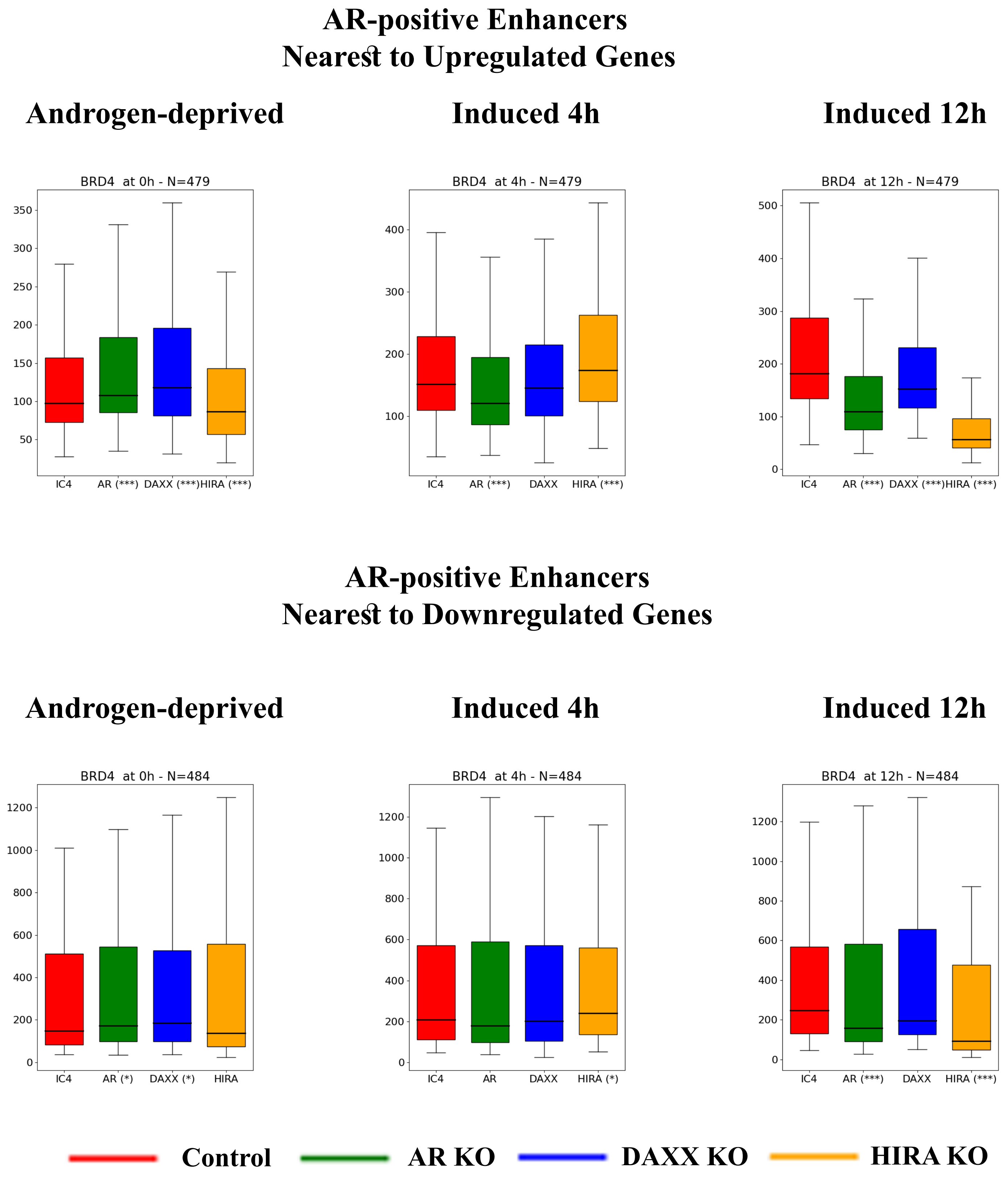

Fig. S12 B. Dynamics of BRD4 at enhancers; analysis within time points.
BRD4 at AR-positive enhancers nearest to 409 up- and 328 downregulated genes in androgen-deprived and induced for 4h, 12h, 24h. X: distance from AR-binding site, bp. Y: arbitrary units

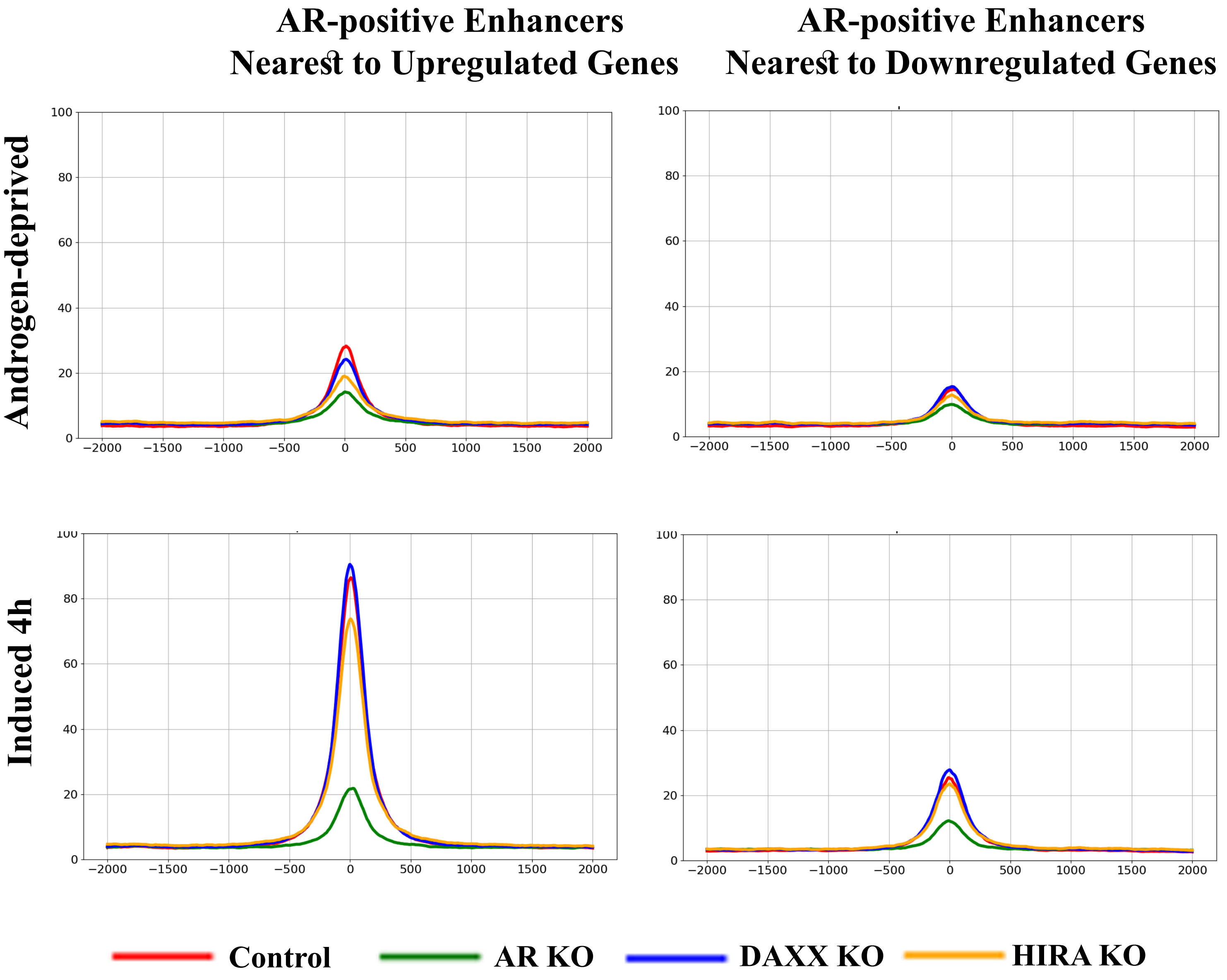

Fig. S13. DNA accessibility analyzed by ATAC-seq at enhancers; analysis within time points. ATAC at AR-positive enhancers nearest to 409 up- and 328 downregulated genes in androgen-deprived and induced for 4h. X: distance from AR-binding site, bp. Y: arbitrary units

Fig. S14A

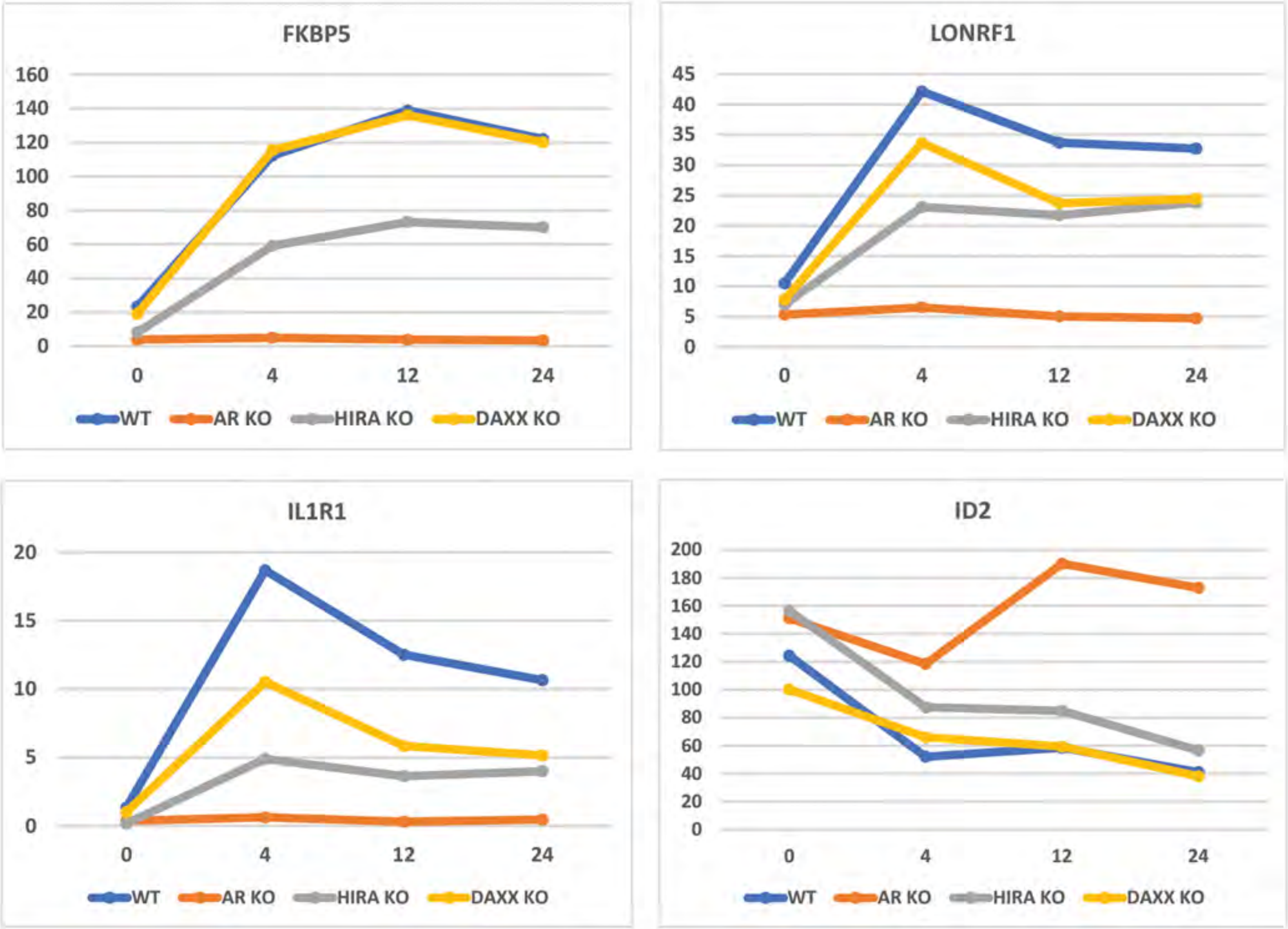

Fig. S14. Examples of epigenetic profiles of enhancers/ SE associated with genes co-regulated by AR and HIRA.
A: Androgen-induced expression of LONRF1, IL1R1, FKBP5 genes is reduced by AR and HIRA KO, and androgen-induced repression of ID2 gene is elevated by AR and HIRAKO (results of RNA-seq analysis).

Fig. S14B

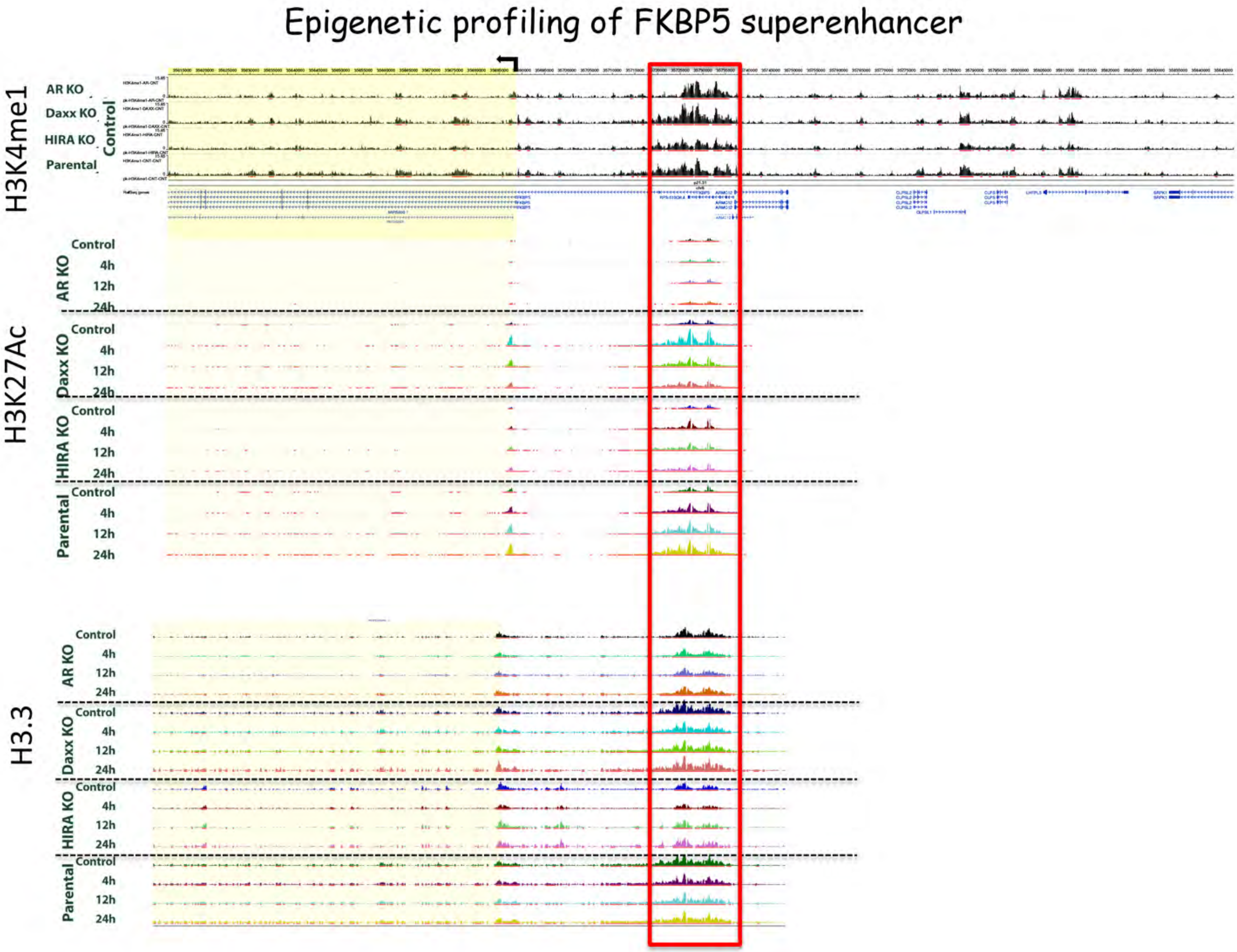

Fig. S14. Examples of epigenetic profiles of enhancers/ SE associated with genes co-regulated by AR and HIRA.
B: H3K4me1 (enhancer), H3K27Ac (active enhancer), H3.3, H3.3S31Ph, AR, BRD4, and CTCF profiling were analyzed by ChIP-seq. DNA accessibility analyzed by ATAC-seq. Arrows: transcription start; red lines outline SE regions.

Fig. S14B

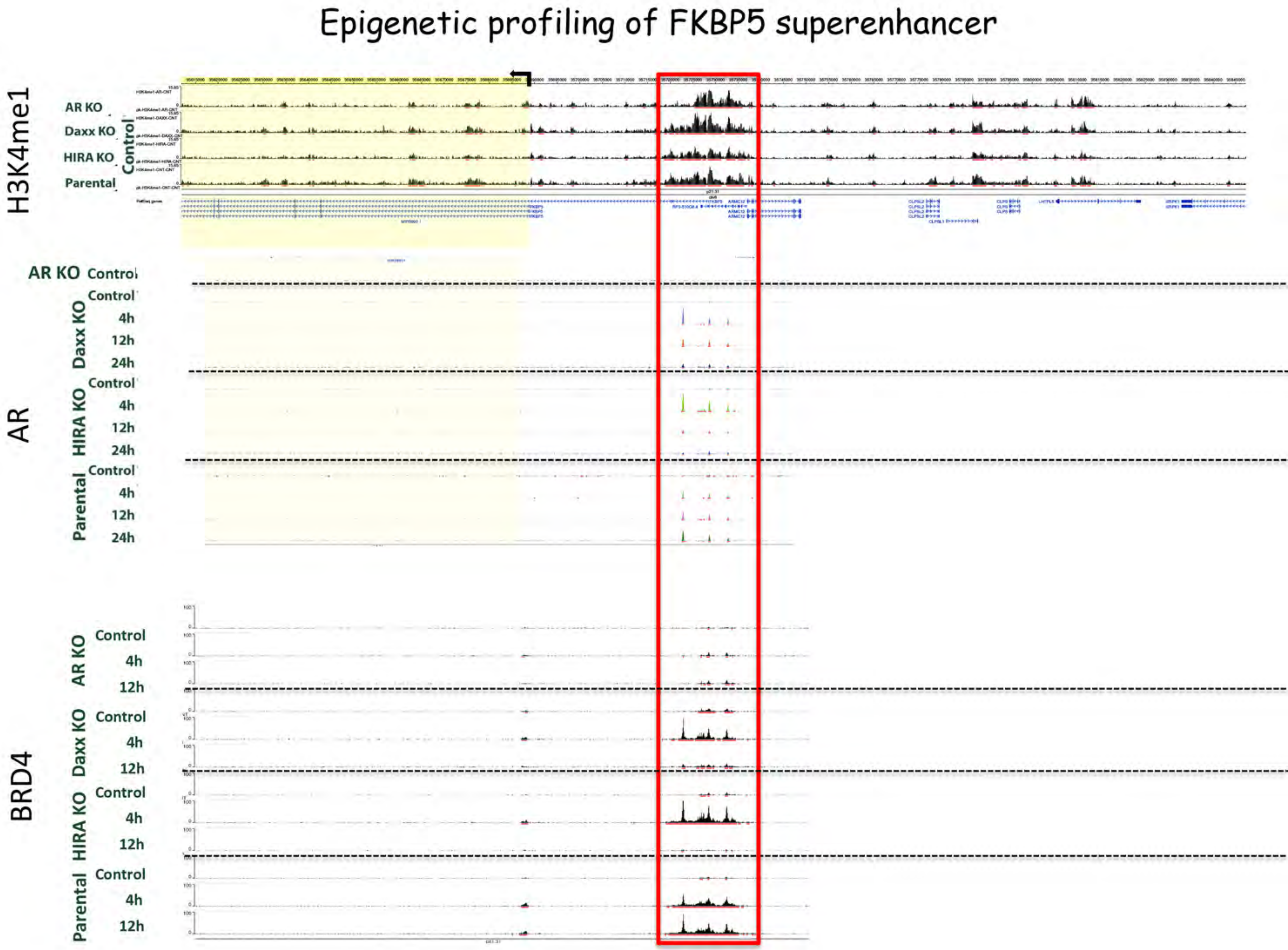
Epigenetic profiling of FKBP5 superenhancer
H3K4me1
AR KO
Daxx KO
HIRA KO
Parental
Control
AR
AR KO Control
Daxx KO
Control
4h
12h
24h
HIRA KO
Control
4h
12h
24h
Parental
Control
4h
12h
24h
BRD4
AR KO
Control
4h
12h
Daxx KO
Control
4h
12h
HIRA KO
Control
4h
12h
Parental
Control
4h
12h

Fig. S14B

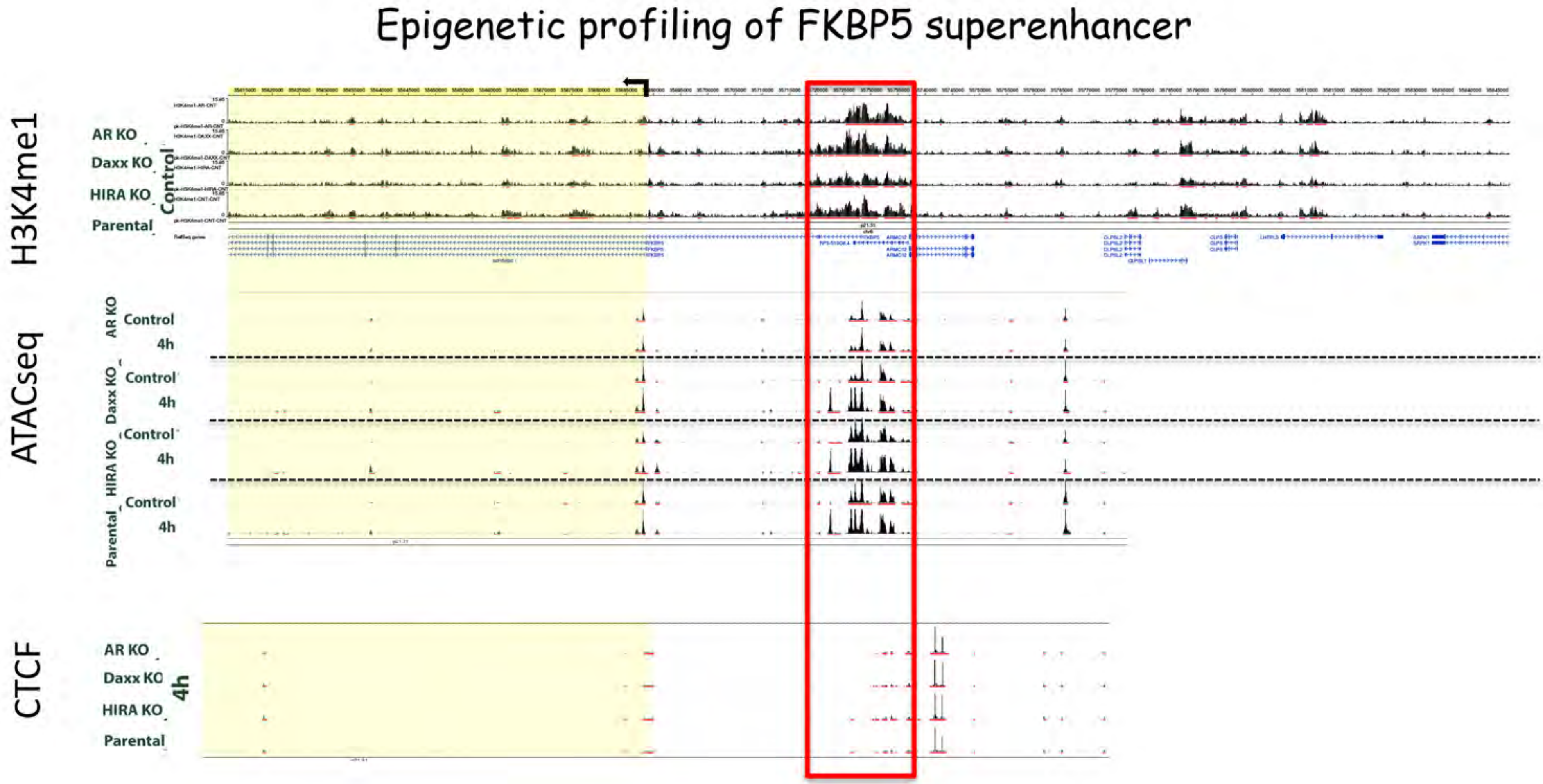
Epigenetic profiling of FKBP5 superenhancer
H3K4me1
AR KO
Daxx KO
HIRA KO
Parental
Control
ATACseq
AR KO
Control
4h
Daxx KO
Control
4h
HIRA KO
Control
4h
Parental
Control
4h
CTCF
AR KO
Daxx KO
HIRA KO
Parental
4h

Fig. S14B

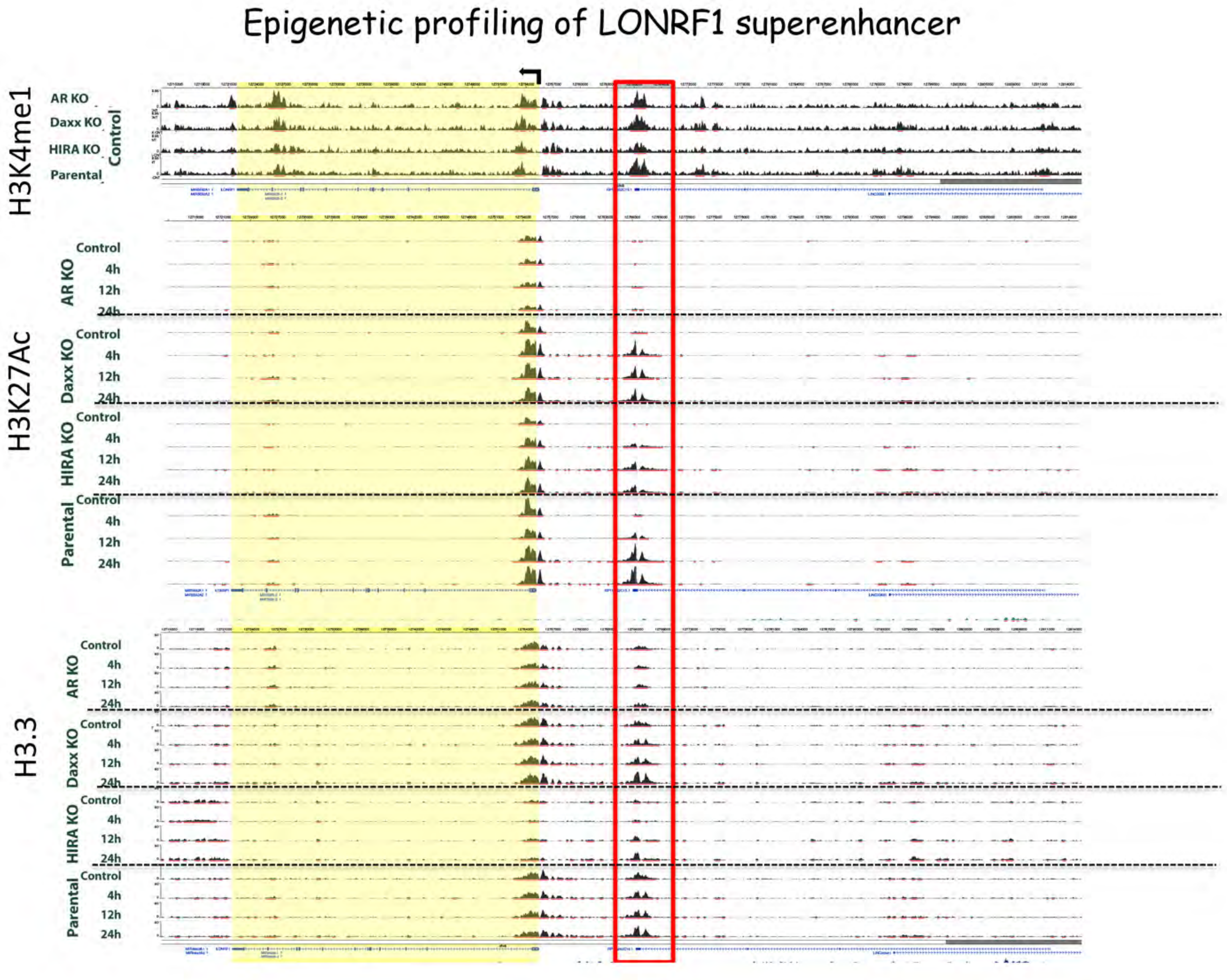
Epigenetic profiling of LONRF1 superenhancer
H3K4me1
AR KO
Daxx KO
HIRA KO
Parental
Control
H3K27Ac
AR KO
Daxx KO
HIRA KO
Parental
Control
4h
12h
24h
H3.3

Fig. S14B

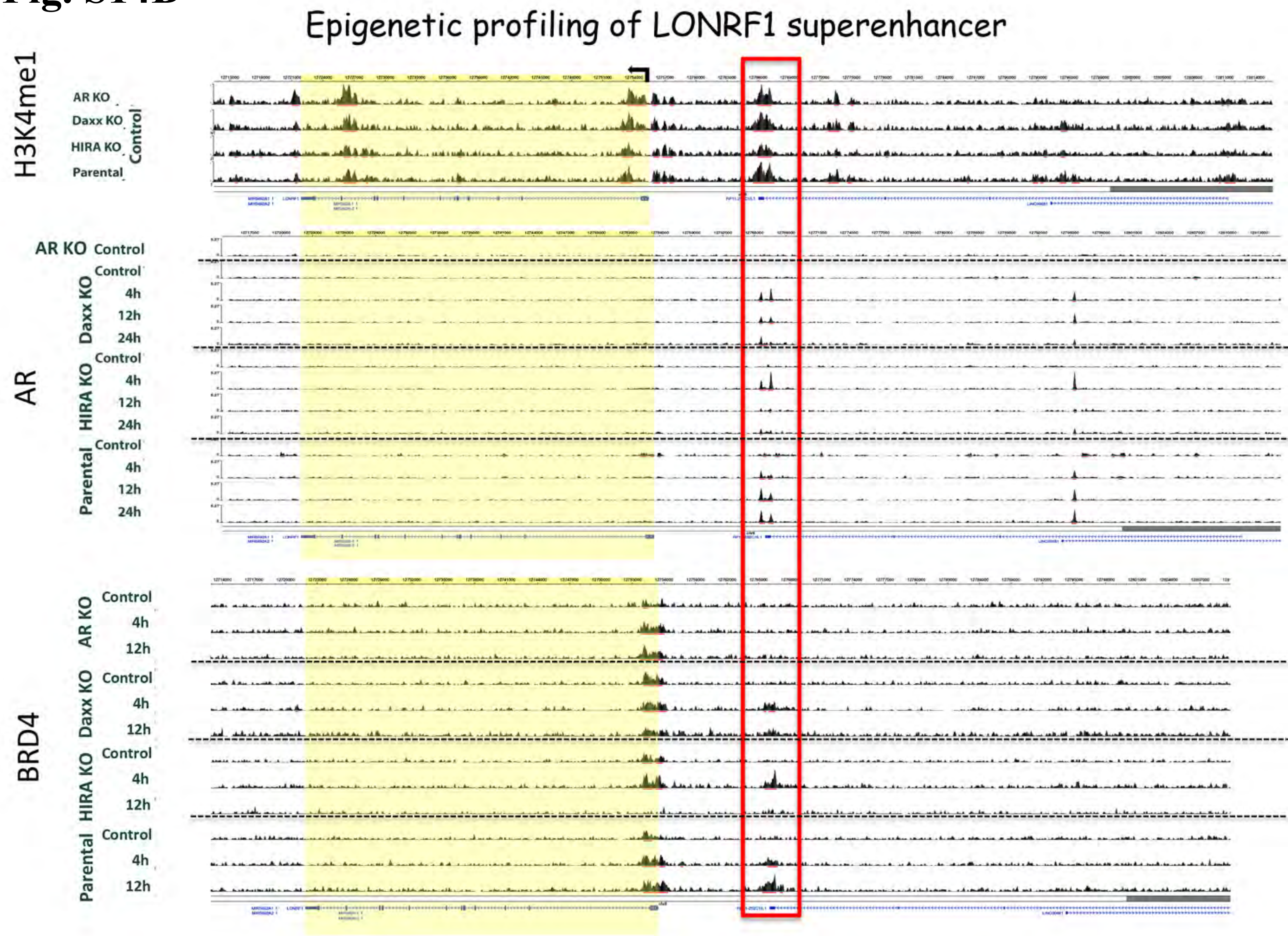
Epigenetic profiling of LONRF1 superenhancer
H3K4me1
AR KO
Daxx KO
HIRA KO
Parental
Control
AR
AR KO Control
Daxx KO
Control
4h
12h
24h
HIRA KO
Control
4h
12h
24h
Parental
Control
4h
12h
24h
BRD4
AR KO
Control
4h
12h
Daxx KO
Control
4h
12h
HIRA KO
Control
4h
12h
Parental
Control
4h
12h

Fig. S14B

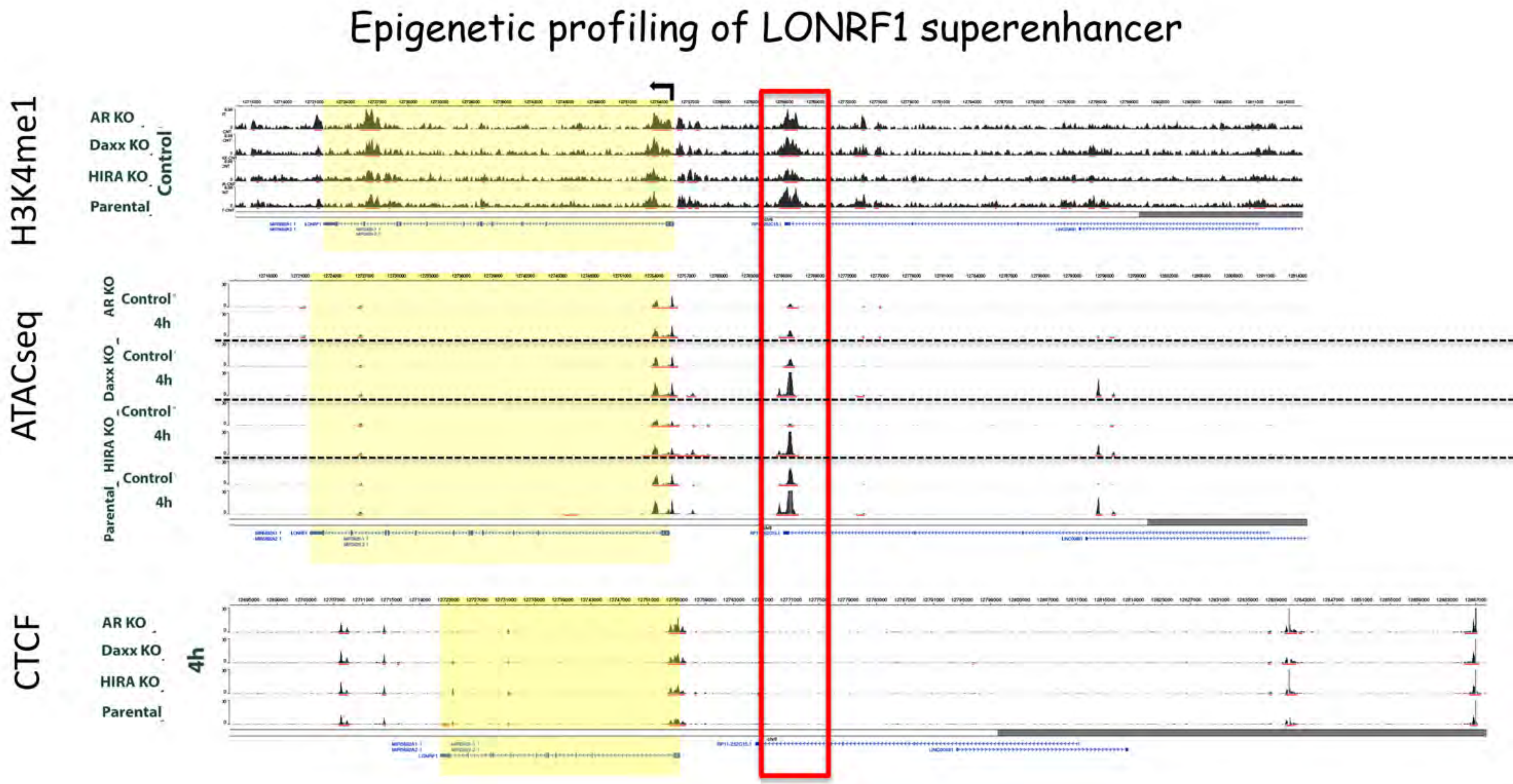
Epigenetic profiling of LONRF1 superenhancer
H3K4me1
AR KO
Daxx KO
HIRA KO
Parental
Control
ATACseq
AR KO
Control
4h
Daxx KO
Control
4h
HIRA KO
Control
4h
Parental
Control
4h
CTCF
AR KO
Daxx KO
HIRA KO
Parental
4h

Fig. S14B

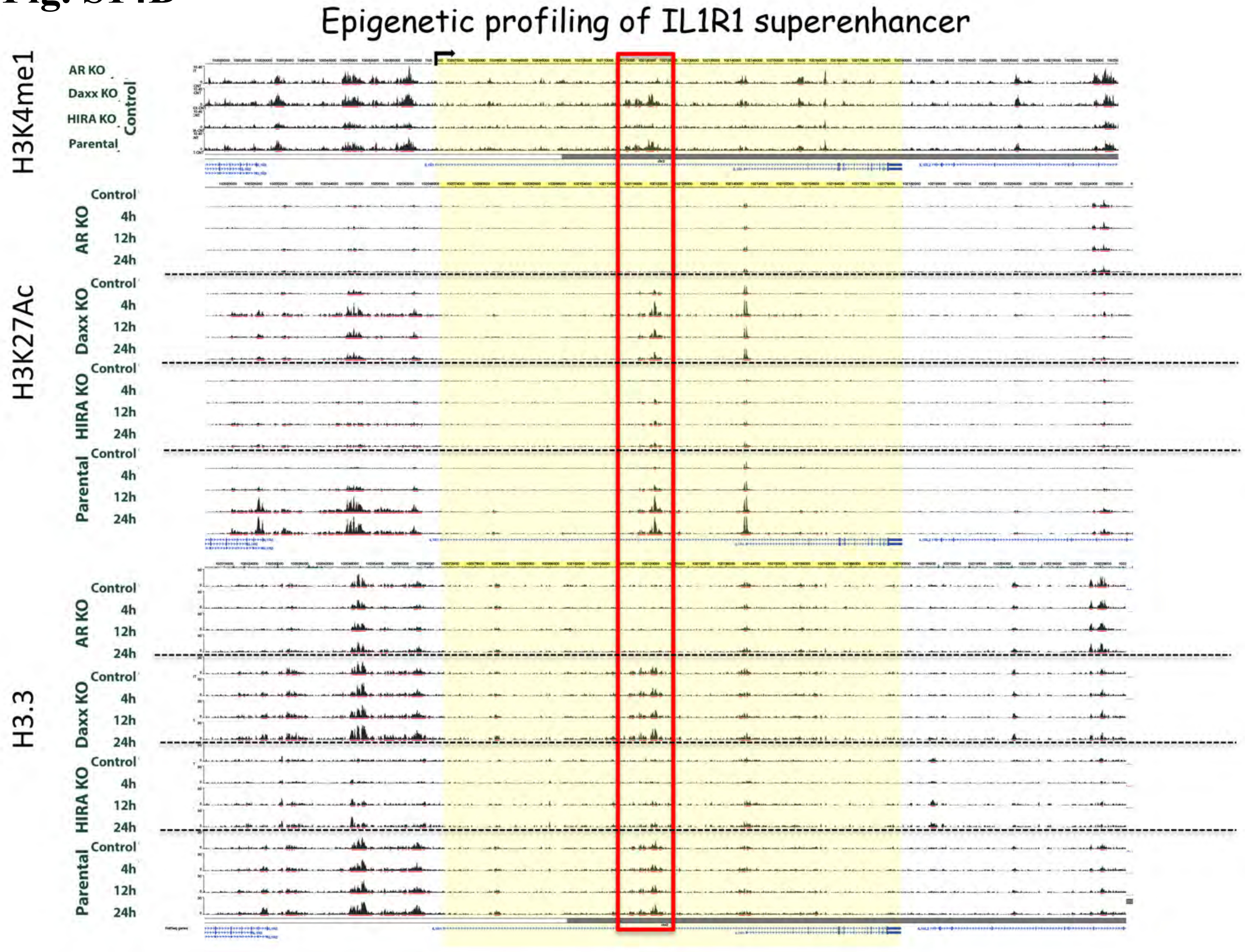

Epigenetic profiling of IL1R1 superenhancer
H3K4me1
AR KO
Daxx KO
HIRA KO
Parental
Control
H3K27Ac
AR KO
Daxx KO
HIRA KO
Parental
Control
4h
12h
24h
H3.3
AR KO
Daxx KO
HIRA KO
Parental
Control
4h
12h
24h

Fig. S14B

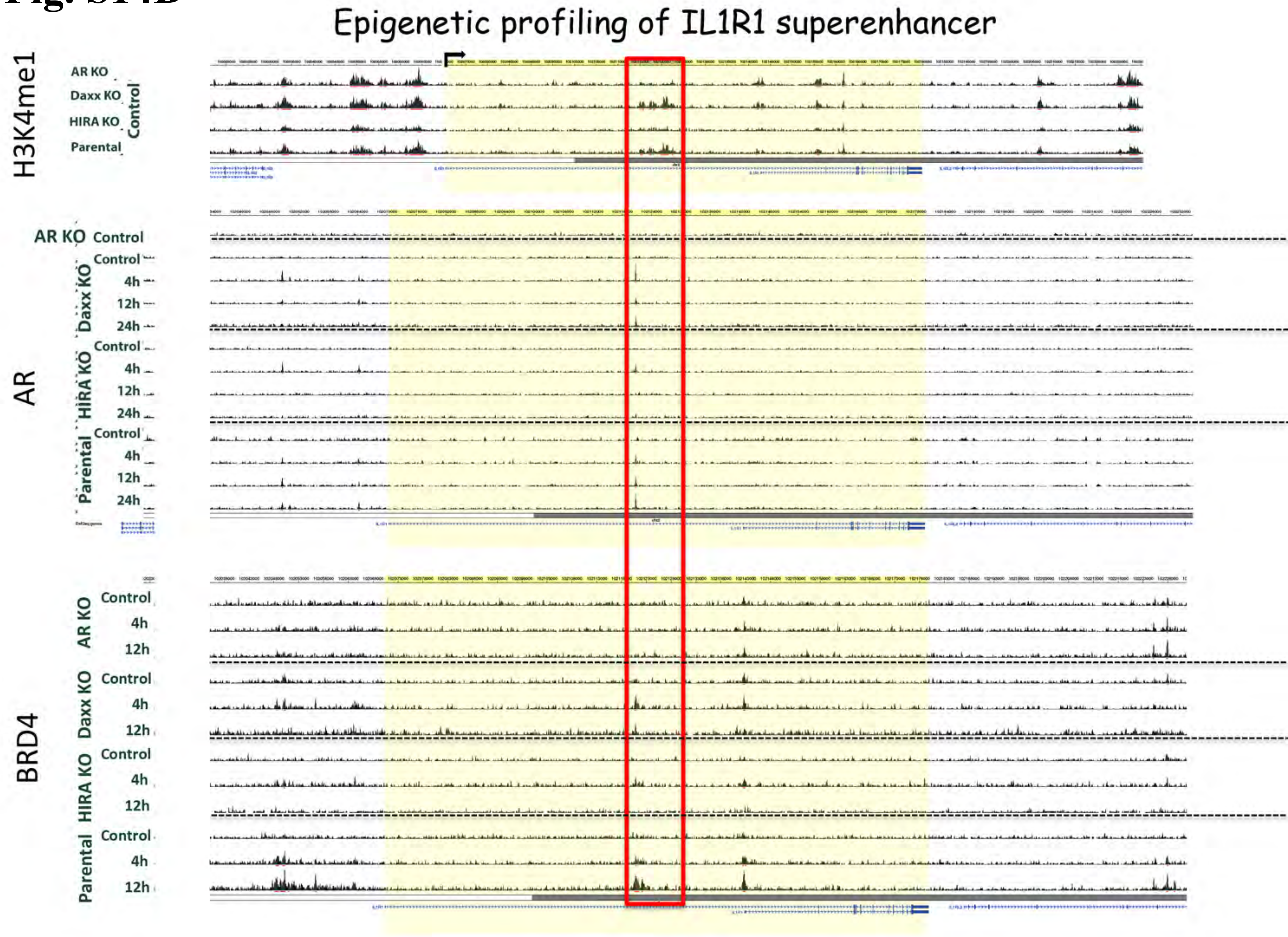

Epigenetic profiling of IL1R1 superenhancer
H3K4me1
AR KO
Daxx KO
HIRA KO
Parental
Control
AR
AR KO Control
Daxx KO
HIRA KO
Parental
Control
4h
12h
24h
BRD4
AR KO
Daxx KO
HIRA KO
Parental
Control
4h
12h

Fig. S14B

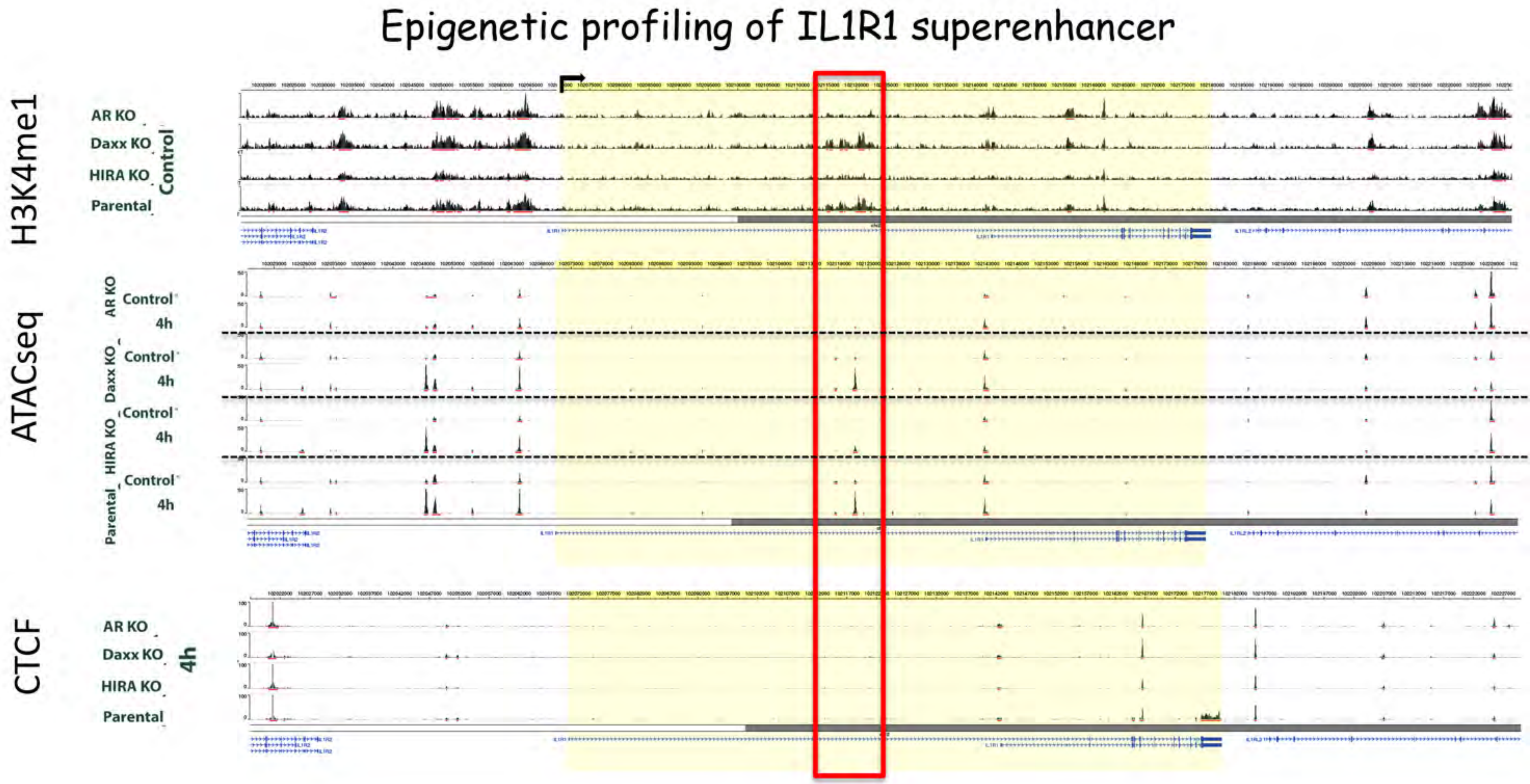
Epigenetic profiling of IL1R1 superenhancer
H3K4me1
AR KO
Daxx KO
HIRA KO
Parental
Control
ATACseq
AR KO
Daxx KO
HIRA KO
Parental
Control
4h
CTCF
AR KO
Daxx KO
HIRA KO
Parental
4h

Fig. S14B

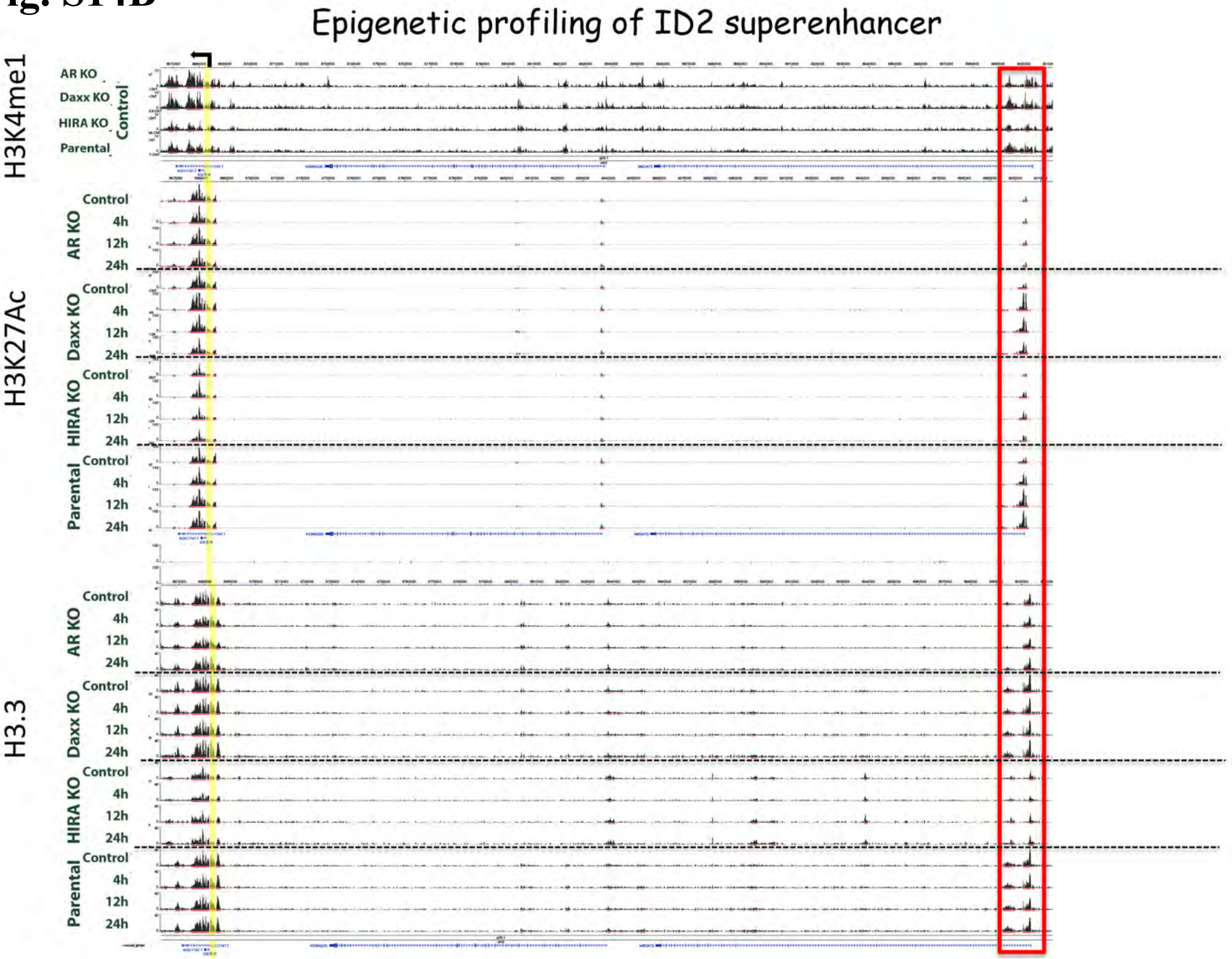
Epigenetic profiling of ID2 superenhancer
H3K4me1
AR KO
Daxx KO
HIRA KO
Parental
Control
H3K27Ac
AR KO
Daxx KO
HIRA KO
Parental
Control
4h
12h
24h
H3.3

Fig. S14B

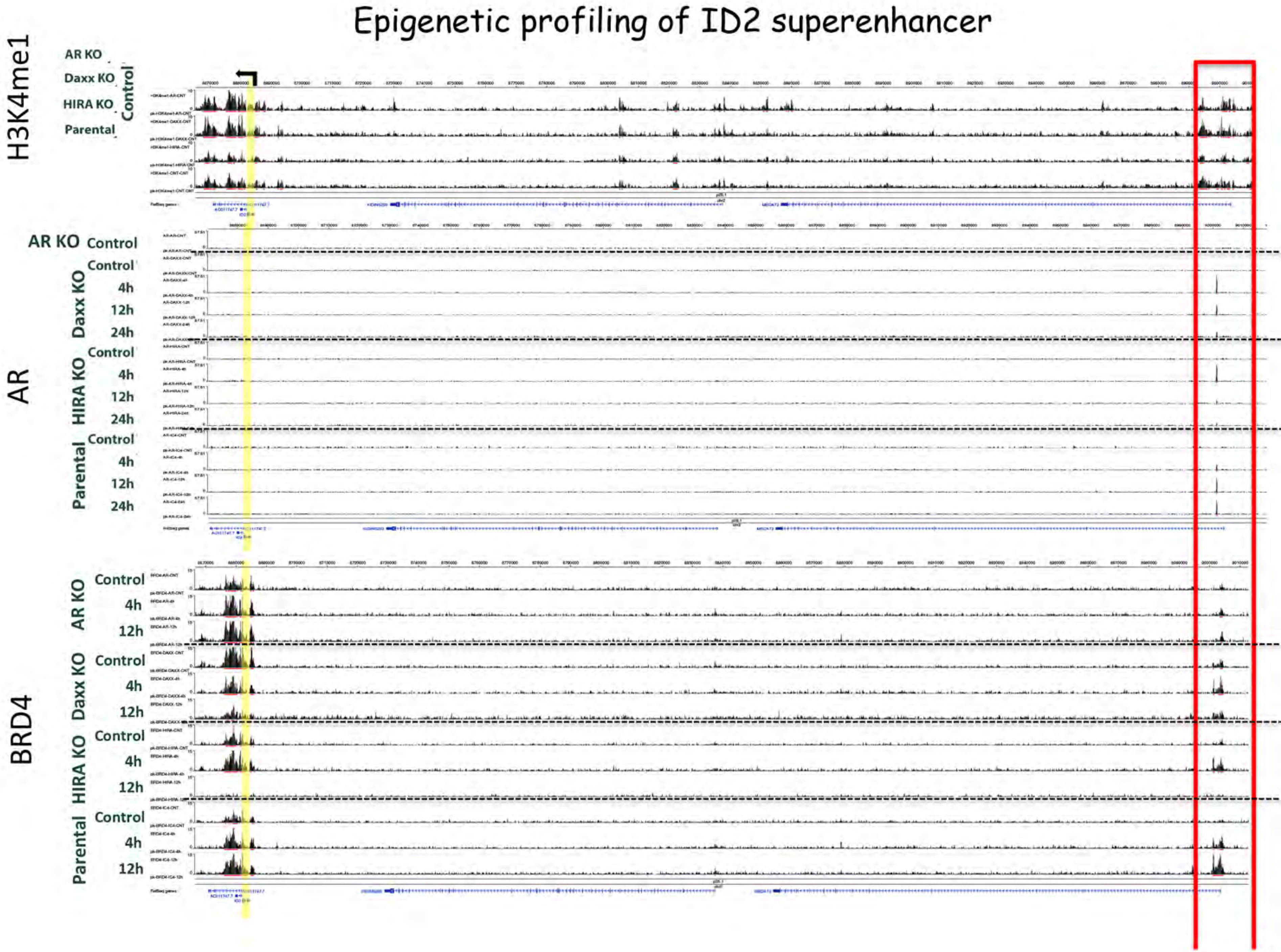
Epigenetic profiling of ID2 superenhancer
H3K4me1
AR KO
Daxx KO
HIRA KO
Parental
Control
AR
AR KO Control
Daxx KO Control
4h
12h
24h
HIRA KO Control
4h
12h
24h
Parental Control
4h
12h
24h
BRD4
AR KO Control
4h
12h
Daxx KO Control
4h
12h
HIRA KO Control
4h
12h
Parental Control
4h
12h

Fig. S14B

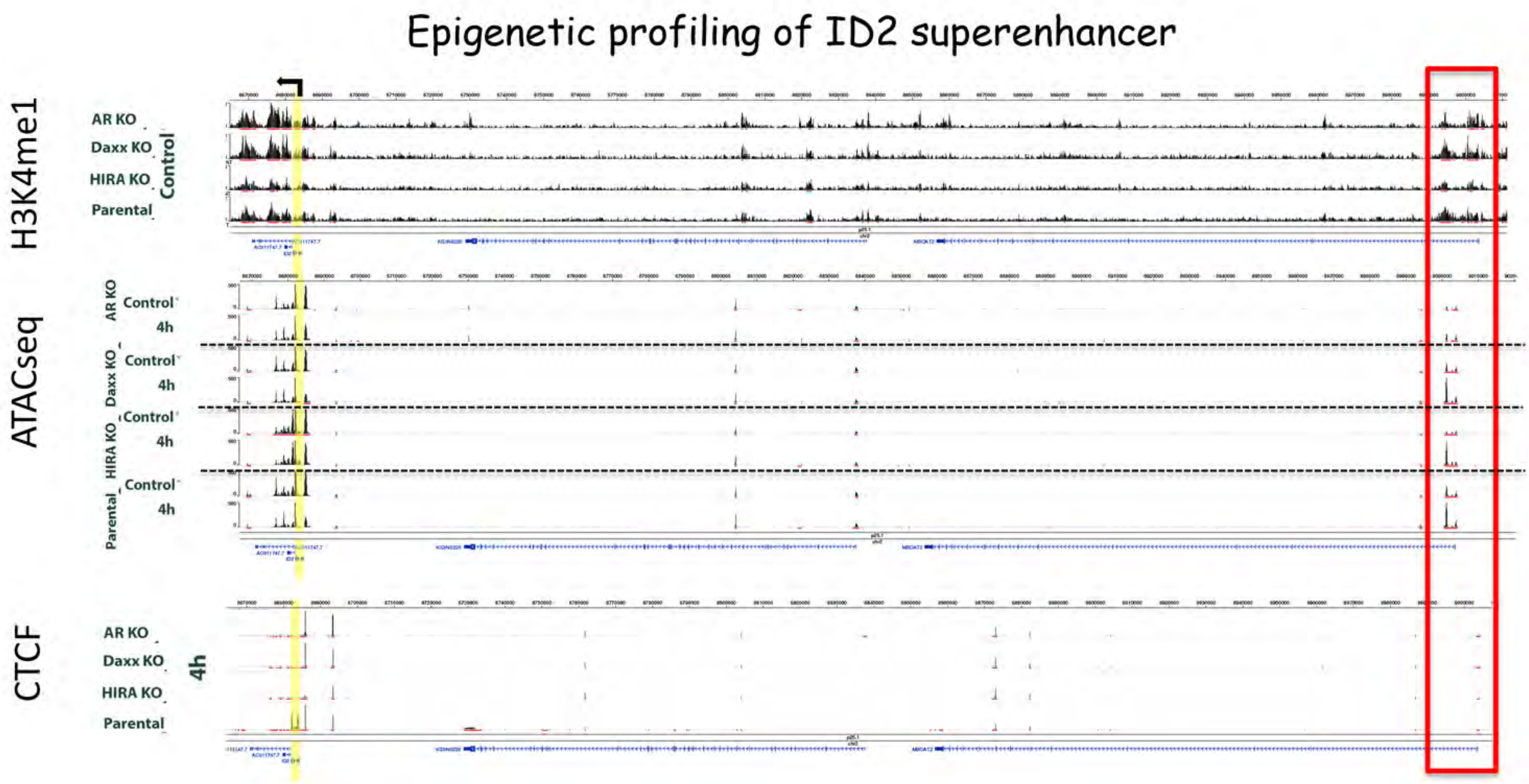
Epigenetic profiling of ID2 superenhancer
H3K4me1
AR KO
Daxx KO
HIRA KO
Parental
Control
ATACseq
AR KO
Control
4h
Daxx KO
Control
4h
HIRA KO
Control
4h
Parental
Control
4h
CTCF
AR KO
Daxx KO
HIRA KO
Parental
4h

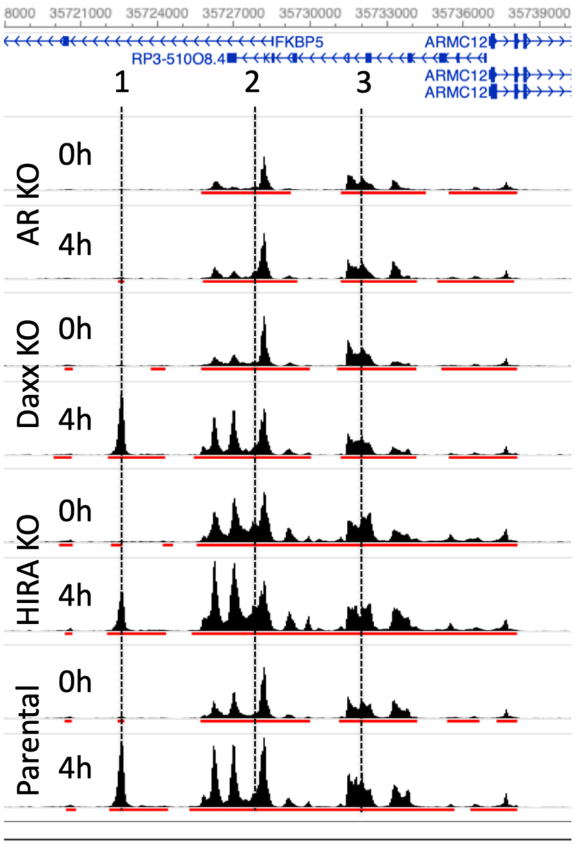

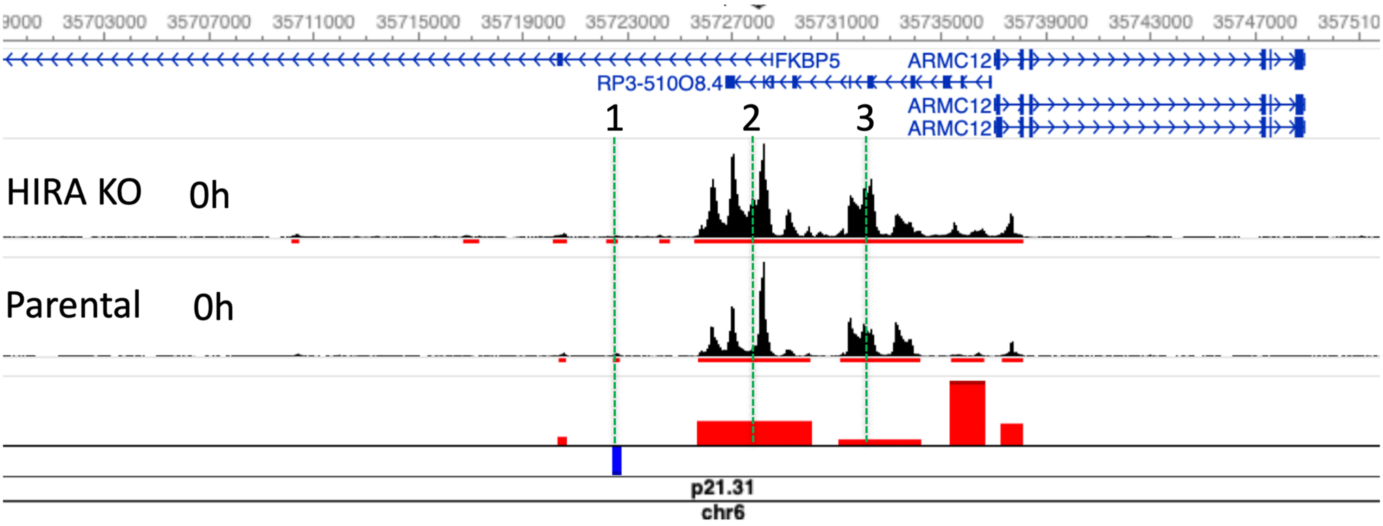

Fig. S15. DNA accessibility profiling at FKBP5 SE.

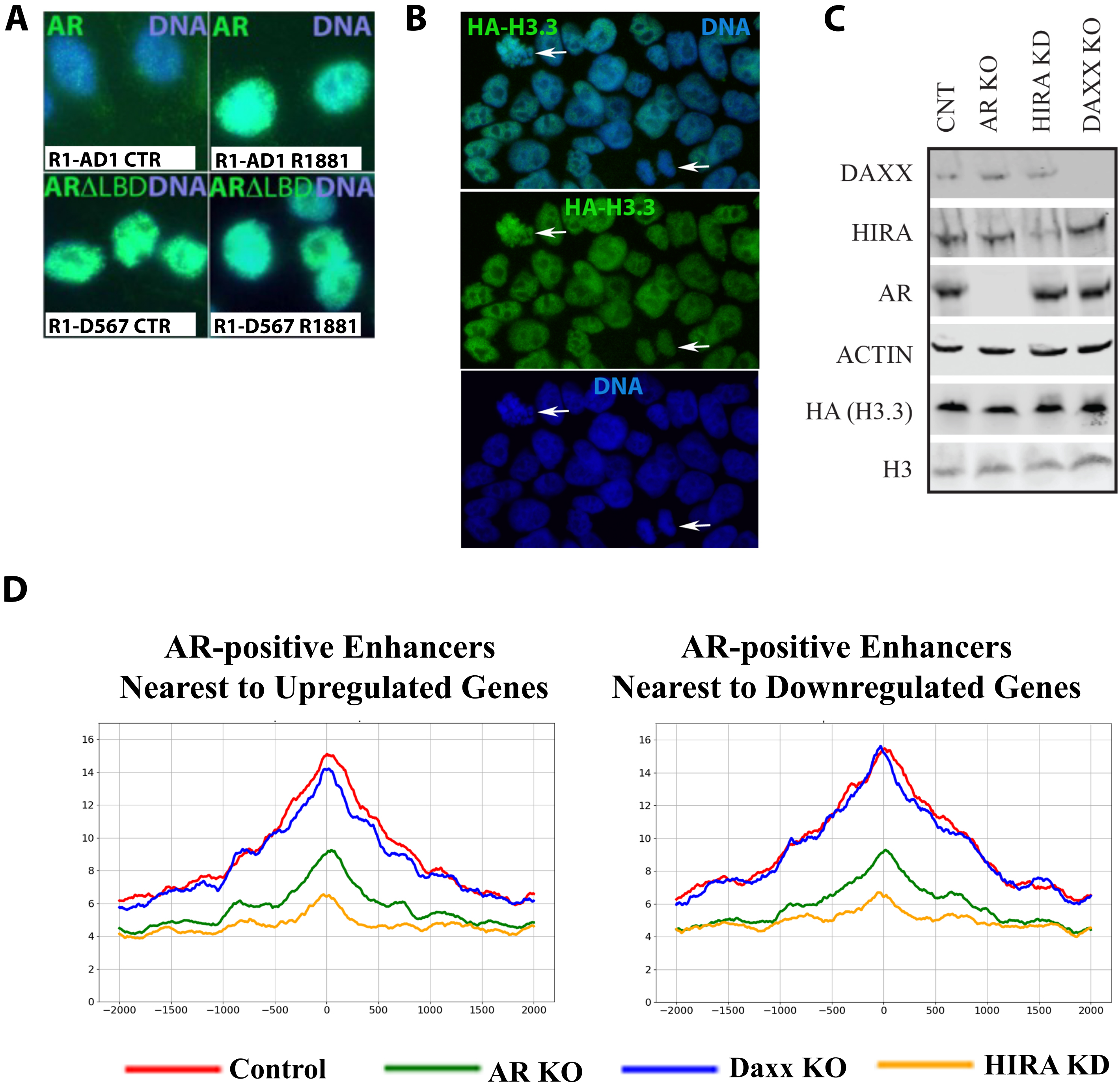

Fig. S16. Characterization of HIRA function in AR-V expressing cells.
A. Intra-cellular localization of AR and AR-DLBD. B. Characterization of FLAG-HA-H3.3 R1-D567 cells. C: KO and knock down (KD) characterization. D. H3.3 profiling at enhancers.X: distance from AR-binding site, bp. Y: arbitrary units

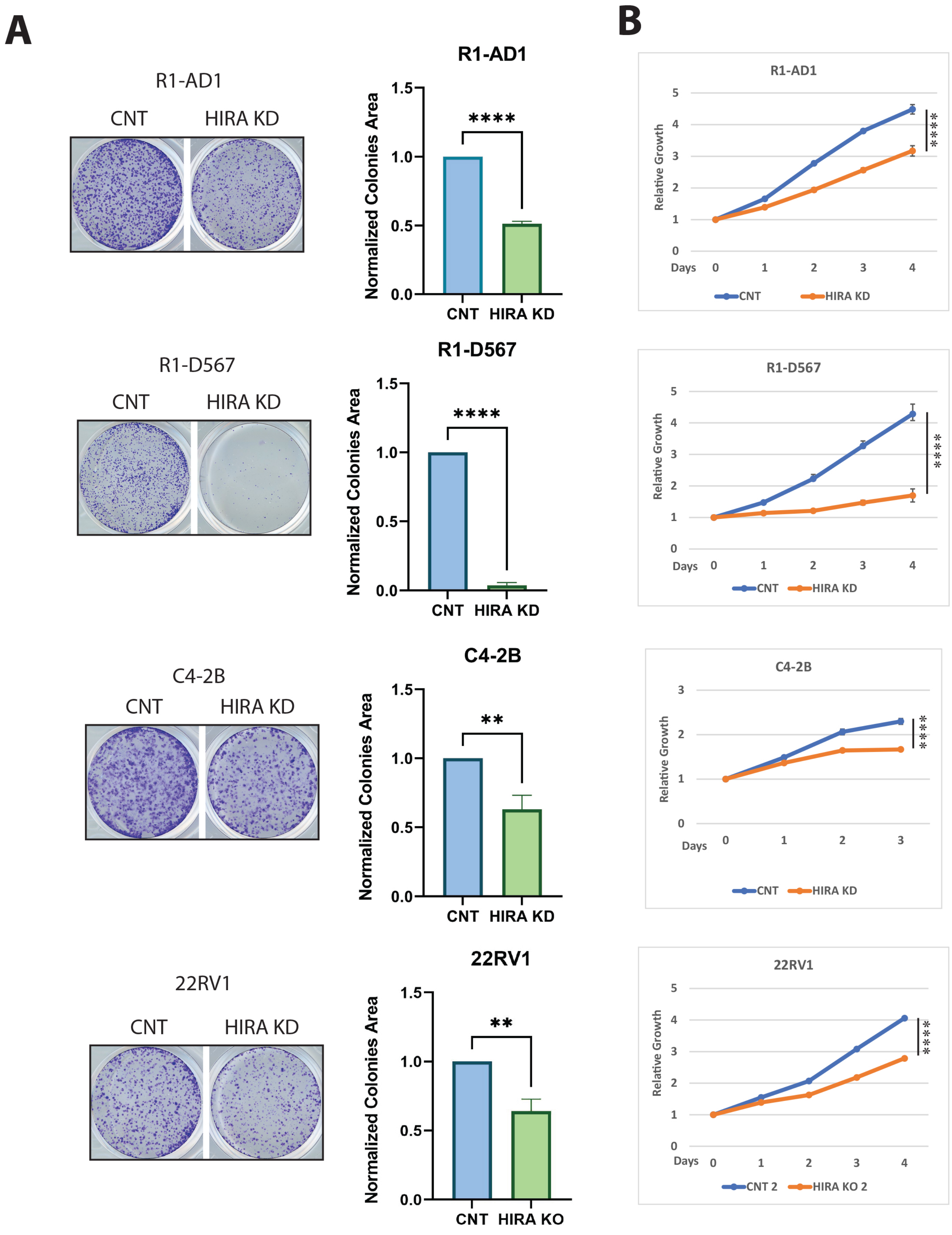

Fig. S17. Knock-down (KD) of HIRA reduces proliferation of PC cells. Effect of HIRA KD tested in R1-AD1 (AR-WT), R1-D567 (AR-DLBD), C4-2B (AR-WT), and 22Rv1 (AR-WT and AR-DLBD). **A:** colony formation assays. Left: representative images; right: normalized colonies area; the height of the bar represents the mean; the error bar represents the SD. **B:** Proliferation of control and HIRA KD PC cells measured by Alamar blue assay. **: $p < 0.01$, **** $p < 0.0001$.

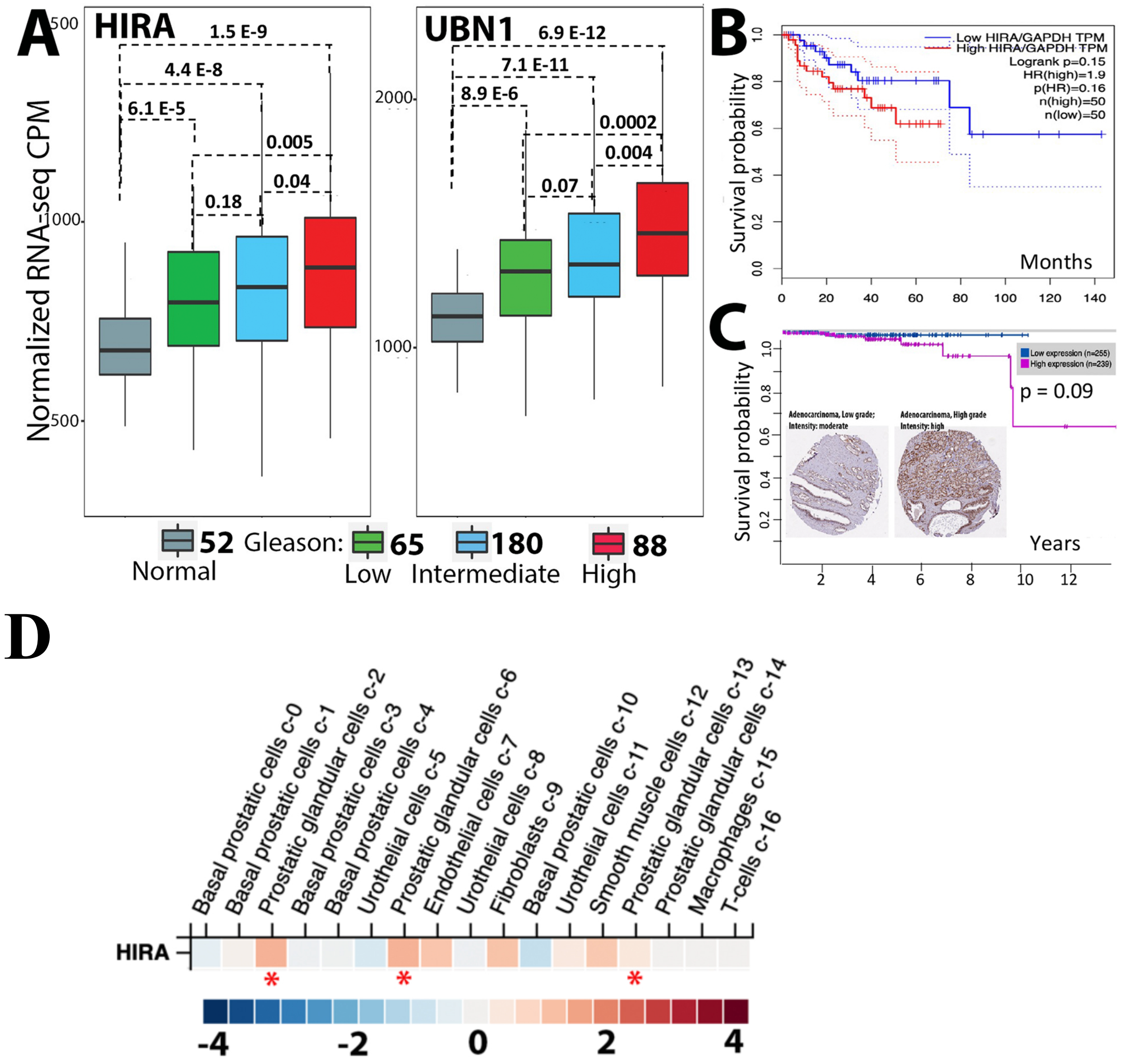

Fig. S18. HIRA complex in PC.

Fig. S1. Production and characterization of R1-AD1 (AR-WT) FLAG-HA-H3.3 knock-in and knockout cells. A: In R1-AD1 cells, CRISPR/Cas9 strategy was used to introduce FLAG-HA tag at 5' end of H3F3A gene by homologous recombination. Single-cell clones were characterized for expression of HA. Microscopy analysis of endogenous tagged FLAG-HA-H3.3 in exponentially grown cells. HA (H3.3): green. DNA: blue. Arrow: mitotic cell confirming chromosome association of FLAG-HA-H3.3. **B:** CRISPR/Cas9 strategy was used to knockout (KO) AR, HIRA, and Daxx in FLAG-HA-H3.3 tagged R1-AD1 cells. Western analysis to confirm KO. Modification of AR, HIRA or Daxx does not affect protein levels of remaining two proteins; HIRA KO reduced levels of H3.3. H3: total histone H3; actin: loading control. **C:** R1-AD1 cells were androgen-deprived (72h) and androgen R1881 stimulated (4h, 12h, 24h); levels of BRD4, H3.3 (HA) and total H3 are shown; actin: loading control.

Fig. S2. Expression analysis (RNA-seq) of R1-AD1 cells, parental (WT), AR KO, Daxx KO and HIRA KO. A: Analysis of genes deregulated by AR KO, Daxx KO, HIRA KO. Venn diagram of genes that are at least two-folds (p-adjusted <0.05) up- and downregulated by 4h of R1881 stimulation (compared with androgen-deprived (72h)) in parental R1-AD1 and in KO cells. **B: Analysis of genes that are deregulated in AR KO and HIRA KO.** Top, Venn diagram; androgen-regulated genes that are affected in AR KO or HIRA KO cells; same parameters as in **A**. Bottom: Heatmaps showing expression of androgen-regulated genes that are affected by both AR KO and HIRA KO identified by KEGG pathway enrichment analysis. Visualization by "pheatmap", http://cran.nexr.com/web/packages/pheatmap/index.html.

Fig. S3. H3.3 association with TSS of androgen-regulated genes; analysis within time points (complementary to Fig. 3). ChIP-seq profiles of H3.3 at TSS of 409 androgen-up- (**left**) and 328 androgen-downregulated (**right**) genes in R1-AD1 parental (Control), AR KO, Daxx KO and HIRA KO cells in the androgen-deprived (0h) and androgen-induced conditions (at 4h, 12h, 24h). 0: TSS.

Fig. S4. H3.3 association with androgen-regulated genes; analysis within cell lines. ChIP-seq profiles of H3.3 at the 409 androgen-up- (**left**) and 328 androgen-downregulated (**right**) genes in R1-AD1 parental (Control), AR KO, Daxx KO and HIRA KO cells in the androgen-deprived (0h) and androgen-induced conditions (at 4h, 12h, 24h). Transcription start sites: TSS; transcription end sites: TES.

Fig. S5. H3K27Ac association with TSS of androgen-regulated genes; analysis within time points (complementary to Fig. 4). ChIP-seq profiles of H3K27Ac at TSS of 409 androgen-up- (**left**) and 328 androgen-downregulated (**right**) genes in R1-AD1 parental (Control), AR KO, Daxx KO and HIRA KO cells in the androgen-deprived (0h) and androgen-induced conditions (at 4h, 12h, 24h). 0: TSS.

Fig. S6. H3K27Ac association with androgen-regulated genes; analysis within cell lines. ChIP-seq profiles of H3K27Ac at the 409 androgen-up- (**left**) and 328 androgen-downregulated (**right**) genes in R1-AD1 parental (Control), AR KO, Daxx KO and HIRA KO cells in the androgen-deprived (0h) and androgen-induced conditions (at 4h, 12h, 24h). Transcription start sites: TSS; transcription end sites: TES.

Fig. S7. AR association with chromatin is regulated by H3.3 chaperone HIRA; analysis within time points (complementary to Fig. 5). A Left: AR analysis of AR peaks (position "0": AR at 4h in R1-AD1 parental cells) in R1-AD1 parental (Control, red), Daxx KO (blue), and HIRA KO (yellow), in androgen-deprived (72h) and R1881 stimulated for 4h, 12h, 24h. Analysis of AR at enhancers associated with 409 up- (**middle**) and 328 downregulated (**right**) genes. **B:** boxplots of total AR signal around AR peaks in enhancers associated with 409 up- and 328 downregulated genes.

Fig. S8. H3K4me1 profiling at enhancers. H3K4me1 at AR-positive enhancers nearest to 409 up- and 328 downregulated genes (top), all AR-positive enhancers (1980; middle left), AR-positive enhancers that are not associated with regulated genes (1489; middle right), all AR-negative enhancers (40644; bottom left) and all enhancers (42624; bottom right). Analysis in R1-AD1 parental (Control, red), AR KO (green), Daxx KO (blue) and HIRA KO (yellow) in androgen-deprived (72h) conditions.

Fig. S9. Dynamics of H3.3 at enhancers; analysis within time points (complementary to Fig. 6). A: Metaplot and **B:** boxplot of H3.3 at AR peaks (position "0": AR at 4h in R1-AD1 parental cells) at enhancers associated with up- (left) and down-regulated (right) genes in R1-AD1 parental (Control, red), AR KO (green), Daxx KO (blue), and HIRA KO (yellow) in androgen-deprived (72h) and R1881 stimulated for 4h, 12h, 24h.

Fig. S10. Dynamics of H3K27Ac at enhancers; analysis within time points (complementary to Fig. 7). A: Metaplot and **B:** boxplot of H3K27Ac ChIP-seq analysis at AR peaks (position “0”: AR at 4h in R1-AD1 parental cells) at enhancers associated with up- (left) and down-regulated (right) genes in R1-AD1 parental (Control, red), AR KO (green), Daxx KO (blue), and HIRA KO (yellow) in androgen-deprived (72h) and R1881 stimulated for 4h, 12h, 24h.

Fig. S11. Dynamics of H3.3 S31ph at enhancers/ SE; analysis within time points (complementary to Fig. 8). A: Metaplot and **B:** boxplot of H3.3 S31ph ChIP-seq analysis at AR peaks (position “0”: AR at 4h in R1-AD1 parental cells) at enhancers associated with up- (left) and down-regulated (right) genes in R1-AD1 parental (Control, red), AR KO (green), Daxx KO (blue) and HIRA KO (yellow) in androgen-deprived (72h) and R1881 stimulated for 4h, 12h.

Fig. S12. Dynamics of BRD4 at enhancers; analysis within time points (complementary to Fig. 9). A: Metaplot and **B:** boxplot of BRD4 ChIP-seq analysis at AR peaks (position “0”: AR at 4h in R1-AD1 parental cells) at enhancers associated with up- (left) and down-regulated (right) genes in R1-AD1 parental (Control, red), AR KO (green), Daxx KO (blue), and HIRA KO (yellow) in androgen-deprived (72h) and R1881 stimulated for 4h, 12h.

Fig. S13. DNA accessibility analyzed by ATAC-seq at enhancers; analysis within time points (complementary to Fig. 10). Metaplot of ATAC-seq analysis at AR peaks (position “0”: AR at 4h in R1-AD1 parental cells) at enhancers associated with up- (left) and down-regulated (right) genes in R1-AD1 parental (Control, red), AR KO (green), Daxx KO (blue), and HIRA KO (yellow) in androgen-deprived (72h) and R1881 stimulated for 4h.

Fig. S14. Examples of epigenetic profiles of enhancers/ SE associated with genes co-regulated by AR and HIRA. R1-AD1 cells (parental, AR, Daxx, HIRA KO) were androgen-deprived for 72h (Control) and induced with 1 nM of R1881 for 4h, 12h, 24h. **A:** Androgen-induced expression of LONRF1, IL1R1, FKBP5 genes is reduced by AR and HIRA KO, and androgen-induced repression of ID2 gene is elevated by AR and HIRAKO (results of RNA-seq analysis). **B:** H3K4me1 (enhancer), H3K27Ac (active enhancer), H3.3, H3.3S31Ph, AR, BRD4, and CTCF profiling were analyzed by ChIP-seq. DNA accessibility analyzed by ATAC-seq. Arrows: transcription start; red lines outline SE regions.

Fig. S15. DNA accessibility profiling at FKBP5 SE. Top: Analysis of ATAC-seq at ARE-1 (1; half-site ARE, overlaps with FOXA1 binding site), ARE-2 and -3 (2 and 3; canonical AREs) at FKBP5 SE in R1-AD1 parental, AR KO, Daxx KO, HIRA KO at the androgen deprived (0h) and stimulated with 1nM of R1881 for 4h. Androgen stimulation for 4h induced ATAC peak at the ARE-1 (half-site) in parental, Daxx and HIRA KO cells. **Bottom:** Zoom-in comparison of ATAC-seq between parental and HIRA KO in androgen-deprived (72h) conditions. Bottom red bars: peaks increase in HIRA KO compared to parental cells. In androgen-deprived conditions, ARE-2 is more accessible in HIRA KO cells compared with parental cells. Vertical dashed lines: positions of AR binding.

Fig. S16. Characterization of HIRA function in AR-V expressing cells. A. Intra-cellular localization of AR and AR-DLBD. R1-AD1 (AR WT) and R1-D567 (AR-DLBD) cells androgen-deprived or treated with 1nM of R1881 for 2h, stained with anti-AR antibody (green); DNA (nuclei) blue. AR-WT accumulates in nuclei after androgen treatment, while AR-DLBD has nuclear localization in untreated and treated cells. **B. Characterization of FLAG-HA-H3.3 R1-D567 cells.** CRISPR/Cas9 strategy was used to tag endogenous H3.3 in R1-D567 cells using same strategy as in R1-AD1 cells. FLAG-HA was introduced at 5' end of H3F3A gene by homologous recombination. Single-cell clones were characterized for expression of HA. Microscopy analysis of endogenous tagged FLAG-HA-H3.3 in exponentially grown cells. HA-H3.3: green. DNA: blue. Arrow: mitotic cells confirming chromosome association of FLAG-HA-H3.3. **C: KO and knock down (KD) characterization.** CRISPR/Cas9 strategy was used to KO AR and Daxx and shRNA to KD HIRA in R1-567 cells. Western analysis to confirm KO and KD. Modification of AR, HIRA or Daxx does not affect protein levels of remaining two proteins. H3: total histone H3; actin: loading control. **D. H3.3 profiling at enhancers.** ChIP-seq analysis of H3.3 (endogenous HA-H3.3) at AR peaks (position "0") at enhancers associated with 215 up- (left) and 185 down-regulated (right) genes in AR KO compared with R1-D567 parental cells. H3.3 plots in parental cells (Control, red), AR KO (green), Daxx KO (blue), and HIRA KD (yellow) in androgen-deprived (72h) conditions.

Fig. S17. Knock-down (KD) of HIRA reduces proliferation of PC cells. Effect of HIRA KD tested in R1-AD1 (AR-WT), R1-D567 (AR-DLBD), C4-2B (AR-WT), and 22Rv1 (AR-WT and AR-DLBD). **A:** colony formation assays. Left: representative images; right: normalized colonies

area; the height of the bar represents the mean; the error bar represents the SD. **B:** Proliferation of control and HIRA KD PC cells measured by Alamar blue assay. **: $p < 0.01$, **** $p < 0.0001$.

Fig. S18. HIRA complex in PC. A: HIRA and UBN1 expression (TCGA) in normal prostate tissue (gray) and prostate adenocarcinoma by Gleason score: 6 = Low, green; 7 = Intermediate, blue; 8-10 = High, red. HIRA and UBN1 expression are increased in PC compared with normal prostate and within Gleason groups. Numbers in legend: samples in group; p-values by Mann-Whitney U test. **B:** Disease free survival analysis based on HIRA expression normalized on GAPDH. Logrank p test (0.15) compares the survival distributions of high (magenta) and low (blue) expression groups. Hazard Rate (HR, the survive model calculated based on Cox proportional hazards model) >1 (1.9) indicates reduced survival of patients with high expression of HIRA. **C:** Kaplan-Meier plots for PC patients with high (magenta) and low (blue) levels of HIRA protein. High levels of HIRA are associated with negative survival prognoses; p: 0.09. Images: representative HIRA staining sections. Analysis at the Human Protein Atlas. **D:** Levels of HIRA in prostate by single cell RNA analysis. The glandular epithelial prostate cells, that are the main origin of prostate adenocarcinoma, have elevated expression of HIRA (compared with basal prostatic and urothelial cells); analysis at the Human Protein Atlas.

Table S1. Androgen-dependent genes deregulated by AR and HIRA KO (complementary to Figs. 2, S2A). List of 688 genes that are at least two-folds (p-adjusted <0.05) up- or down-regulated by R1881 stimulation in parental R1-AD1 cells and are affected in both AR KO and HIRA KO cells.

Table S2. Pathways deregulation by HIRA and AR KO (complementary to Figs. 2, S2B). List of androgen-regulated genes enriched in pathways identified with KEGG databases that are affected by both AR KO and HIRA KO.

Table S3. The differentially expressed genes after androgen or glucocorticoid treatment. A. Number of differentially expressed genes in response to androgen or glucocorticoid treatment ($|Log2(FC)| > 2$; $p < 0.05$). B. Percentages of differentially expressed genes in each group relatively to the parental cell line (WT, 100%).
